# NeuroML: A Language for Describing Data Driven Models of Neurons and Networks with a High Degree of Biological Detail

**DOI:** 10.1371/journal.pcbi.1000815

**Published:** 2010-06-17

**Authors:** Padraig Gleeson, Sharon Crook, Robert C. Cannon, Michael L. Hines, Guy O. Billings, Matteo Farinella, Thomas M. Morse, Andrew P. Davison, Subhasis Ray, Upinder S. Bhalla, Simon R. Barnes, Yoana D. Dimitrova, R. Angus Silver

**Affiliations:** 1 Department of Neuroscience, Physiology and Pharmacology, University College London, London, United Kingdom; 2 School of Mathematical and Statistical Sciences, School of Life Sciences, and Center for Adaptive Neural Systems, Arizona State University, Tempe, Arizona, United States of America; 3 Textensor Limited, Edinburgh, United Kingdom; 4 Department of Computer Science, Yale University, New Haven, Connecticut, United States of America; 5 Department of Neurobiology, Yale University School of Medicine, New Haven, Connecticut, United States of America; 6 Unité de Neurosciences, Information et Complexité, CNRS, Gif sur Yvette, France; 7 National Centre for Biological Sciences, TIFR, UAS-GKVK Campus, Bangalore, India; University College London, United Kingdom

## Abstract

Biologically detailed single neuron and network models are important for understanding how ion channels, synapses and anatomical connectivity underlie the complex electrical behavior of the brain. While neuronal simulators such as NEURON, GENESIS, MOOSE, NEST, and PSICS facilitate the development of these data-driven neuronal models, the specialized languages they employ are generally not interoperable, limiting model accessibility and preventing reuse of model components and cross-simulator validation. To overcome these problems we have used an Open Source software approach to develop NeuroML, a neuronal model description language based on XML (Extensible Markup Language). This enables these detailed models and their components to be defined in a standalone form, allowing them to be used across multiple simulators and archived in a standardized format. Here we describe the structure of NeuroML and demonstrate its scope by converting into NeuroML models of a number of different voltage- and ligand-gated conductances, models of electrical coupling, synaptic transmission and short-term plasticity, together with morphologically detailed models of individual neurons. We have also used these NeuroML-based components to develop an highly detailed cortical network model. NeuroML-based model descriptions were validated by demonstrating similar model behavior across five independently developed simulators. Although our results confirm that simulations run on different simulators converge, they reveal limits to model interoperability, by showing that for some models convergence only occurs at high levels of spatial and temporal discretisation, when the computational overhead is high. Our development of NeuroML as a common description language for biophysically detailed neuronal and network models enables interoperability across multiple simulation environments, thereby improving model transparency, accessibility and reuse in computational neuroscience.

## Author Summary

Computer modeling is becoming an increasingly valuable tool in the study of the complex interactions underlying the behavior of the brain. Software applications have been developed which make it easier to create models of neural networks as well as detailed models which replicate the electrical activity of individual neurons. The code formats used by each of these applications are generally incompatible however, making it difficult to exchange models and ideas between researchers. Here we present the structure of a neuronal model description language, NeuroML. This provides a way to express these complex models in a common format based on the underlying physiology, allowing them to be mapped to multiple applications. We have tested this language by converting published neuronal models to NeuroML format and comparing their behavior on a number of commonly used simulators. Creating a common, accessible model description format will expose more of the model details to the wider neuroscience community, thus increasing their quality and reliability, as for other Open Source software. NeuroML will also allow a greater “ecosystem” of tools to be developed for building, simulating and analyzing these complex neuronal systems.

## Introduction

Understanding how high level brain function arises from low level mechanisms such as ion channels, synaptic transmission, neuronal integration and complex three dimensional (3D) network connectivity requires detailed computational models with biologically realistic features that are able to link different levels of description and measurement. Models with detailed neuronal morphologies, Hodgkin-Huxley type voltage-gated membrane conductances, and phenomenological synaptic inputs have been used to explore the determinates of action potential firing patterns and information processing in single neurons [1]–[10]. This compartmental neuronal modeling approach [11], which arose from the pioneering work of Rall [12], has also been used to investigate the cellular basis of network behavior in various brain regions in both health and disease. This includes investigation of synchronous activity [13], [14], oscillations [15]–[17], sensory representation [18], [19], locomotion [20] and memory [21] together with the causes of epileptiform activity [15], [22], [23]. Unfortunately, the diverse software that has been used to construct these models together with their specialized nature has restricted the wider use of such models within neuroscience.

A number of dedicated software packages are available for creating and simulating neuronal and network models [24] including NEURON [25], GENESIS [26], MOOSE [27], NEST [28] and PSICS (http://www.psics.org). While dedicated simulators aid the creation of complex models, the multitude of simulator specific programming languages restricts accessibility. Moreover, reproducing a model based on the detailed description in the associated paper is often difficult. This splintering of the available technology has also hindered the sharing and reuse of model components and the development of new tools for detailed computational modeling. This situation contrasts with the field of systems biology [29] which has benefited from the emergence of Extensible Markup Language (XML) based standards for describing biochemical network interactions (e.g. SBML [30], CellML [31]) and curated databases of models [32], allowing greater interoperability and validation of model behavior across multiple simulators. For this reason, model sharing together with greater accessibility and interoperability of neuronal models have been identified as key areas of focus by several recent reports on neuroinformatics [33]–[35]. However, the task of developing simulator-independent standards for describing the myriad of mechanisms and anatomical structures in the brain is considerably more complex than formalizing reaction schemes in systems biology.

The concept of a Neural Open Markup Language (NeuroML, http://www.neuroml.org) for neuronal model description was first proposed by Goddard et al. [36], who extended previous work by Gardner et al. [37]. Building on the ideas in this initial work, we have designed, developed and implemented a structure for NeuroML that can describe models of neuronal systems at various scales in a simulator independent manner. Models of neuronal systems can vary greatly in the amount of biological detail incorporated [6]. The latest version of NeuroML (v1.8.1) focuses on expressing detailed neuronal models which can include complex neuronal morphologies [38], descriptions of voltage- and ligand-gated conductances, synaptic mechanisms and the positions of cells and synaptic connections in a 3D network structure. Here we provide an overview of the structure of the language, illustrate its functionality by expressing a number of complex cell and network models in NeuroML and demonstrate the interoperability and model portability it enables by reproducing model behavior on multiple independently developed simulators.

## Results

### Structure of NeuroML language and technologies used

**Figure 1:**
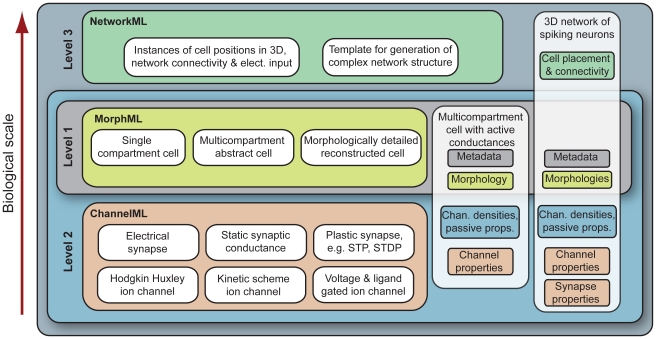
Relationship between the three Levels of NeuroML and MorphML, ChannelML and NetworkML. Level 1 incorporates MorphML, which allows descriptions of cell structure ranging from single compartment cells to detailed cells based on morphological reconstructions. Metadata describing the provenance of the data (authors, citations, etc.) can be used at this and subsequent Levels. Level 2 builds on Level 1 to specify the passive properties and the location and densities of active conductances on the cell, and includes ChannelML, for description of the membrane processes that generate the electrophysiological behavior of cells. Level 3 contains NetworkML, allowing networks of these neuronal models and their synaptic connections to be described. MorphML, ChannelML and NetworkML can be used in isolation to describe model components, while a Level X file can include any elements from that and any lower Level.

The three Level structure of NeuroML partitions model descriptions into the anatomical structure and the various physiological mechanisms that underlie the electrical behavior of neurons and networks and reflects the manner in which they are commonly implemented in neuronal simulators (Figure 1). Level 1 of NeuroML allows description of the neuronal morphology (in MorphML [38]) and relevant background data (metadata) associated with the model. Level 2 of NeuroML builds on this in two ways: it can be used to extend Level 1 cell descriptions to include passive and active electrical properties and it includes ChannelML, which describes voltage-gated membrane conductances together with static and plastic synaptic conductance processes. Descriptions of neural networks are specified in Level 3. This Level includes NetworkML, which specifies the 3D locations of neurons, connections between populations, and external electrical inputs. This modular structure, together with the use of distinct schemas (i.e. MorphML, ChannelML and NetworkML) is designed to enable the exchange and reuse of the individual components between a wide variety of software applications. Descriptions in higher Levels of NeuroML can build on components from lower Levels (Figure 1, Materials and Methods). A full description of the model elements and the files used to specify them is provided in Supporting Text S1.

**Figure 2:**
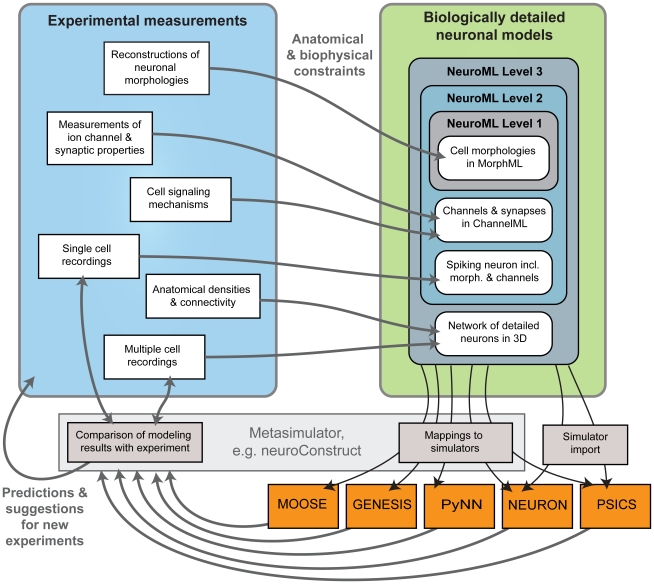
Relationship between experimental data and model components expressed in NeuroML. Experimental neuroscience data is measured at different scales describing subcellular, cellular and network properties and NeuroML provides a framework to describe models developed using this data at all of these levels. Once models are defined in NeuroML they can either be directly imported into a simulator or translated via a metasimulator like neuroConstruct. Optimization of such data-driven models involves an iterative process of experimentation, creation of models, comparison with data and refinement of models, and suggestions for new experiments based on modeling results.

To achieve a high degree of biological detail, data-driven compartmental models utilize data from neuronal reconstructions, measured properties of membrane and synaptic conductances, single and multiple cell electrophysiological recordings and density and connectivity data. The relationship between each of these data types and the various Levels and modular components of NeuroML is illustrated in Figure 2. Models in NeuroML format can be directly imported into applications or automatically mapped onto them using a metasimulator (e.g. neuroConstruct [39]) and simulation results can be used to make predictions that can be tested experimentally.

**Figure 3:**
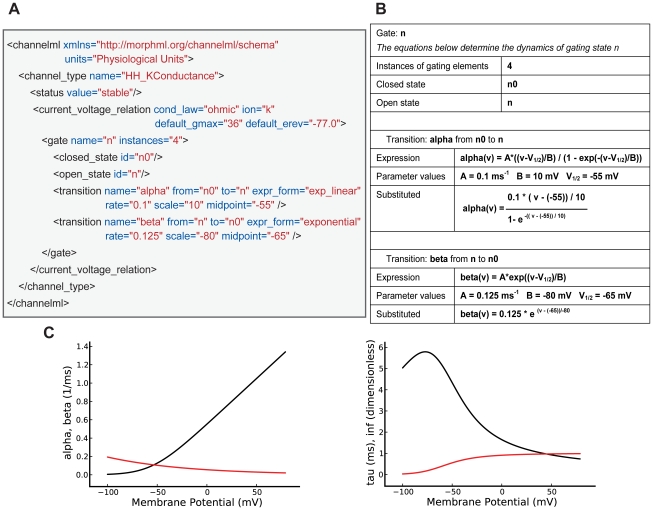
XML structure of a ChannelML file and mappings to text and graphs. (A) A ChannelML file containing a Hodgkin-Huxley type K^+^ conductance model, with four instances of a gating mechanism with open and closed states, and the rates of transitions between them. Supporting Text S1 contains a description of each of the elements contained in this file, and section 10.2 of that document outlines in more detail the equations behind a channel model expressed in ChannelML. (B) A section of a HTML page automatically generated from the ChannelML using an XML Stylesheet (XSL) file. (C) Top: plots of the forward (alpha, black) and reverse (beta, red) transition rates. Bottom: the time constant (tau) of the transition (black) and steady state of the gating variable (inf, red). These views of the contents of the ChannelML file can be generated automatically (e.g. by neuroConstruct) for any valid file.

NeuroML is an Open Source project (http://sourceforge.net/projects/neuroml) and the specifications are based on XML [40], a widely used language for exchanging structured information between computer applications, which has been used previously in other standardization initiatives e.g. SBML [30], CellML [31], BrainML [41] and MathML [42]. Figure 3 shows an example of a ChannelML file with the set of parameters required to fully describe an instance of a voltage-gated K^+^ channel in the Hodgkin-Huxley formalism (See Supporting Text S1 for a description of the current and conductance which would result from this type of channel model). This XML document is a text file containing structured data (Figure 3A), which can be parsed with freely available software libraries (i.e. with minimal effort for application developers) and can be easily transformed into a human-readable form (Figure 3B, Materials and Methods). Moreover, the properties of the specified model can be readily visualized in graphical form (Figure 3C).

Rather than requiring the restructuring of neuronal simulators to a common internal model based on NeuroML, our approach to enhance interoperability and transparency identifies the useful elements that can be exchanged between computational neuroscience tools (morphologies, channels, synapses, network structure etc.) and develops the means to import and export these in a standardized format. This approach allows researchers to develop new neuronal and network models using the application of their choice for maximum flexibility, and then convert these models to NeuroML format for cross simulator validation, increased accessibility and storage. This also means that the models are run using a simulator's own internal data structures, so there is no loss of execution performance compared to creating the models from scratch in the simulator's own script.

NeuroML differs from the model description approaches taken by SBML and CellML, which can describe a variety of models of dynamical systems in biology using low level concepts such as compartments, variables and reaction rates. In contrast NeuroML incorporates many higher level concepts such as Hodgkin-Huxley models of ion channels, synaptic conductance waveforms, synaptic plasticity models, 3D dendritic and axonal structures and 3D network connectivity, because the neuronal models it describes cover many levels of description from ion channels to whole networks. Indeed it is intended for describing models containing the established neurophysiological entities most commonly used when modeling biologically detailed neural systems. While this limits the scope of biological models that can be expressed in this format, it ensures that a wide range of detailed neuronal models in use today can be specified in a dedicated language and facilitates mapping of the models to widely used simulation tools.

**Figure 4:**
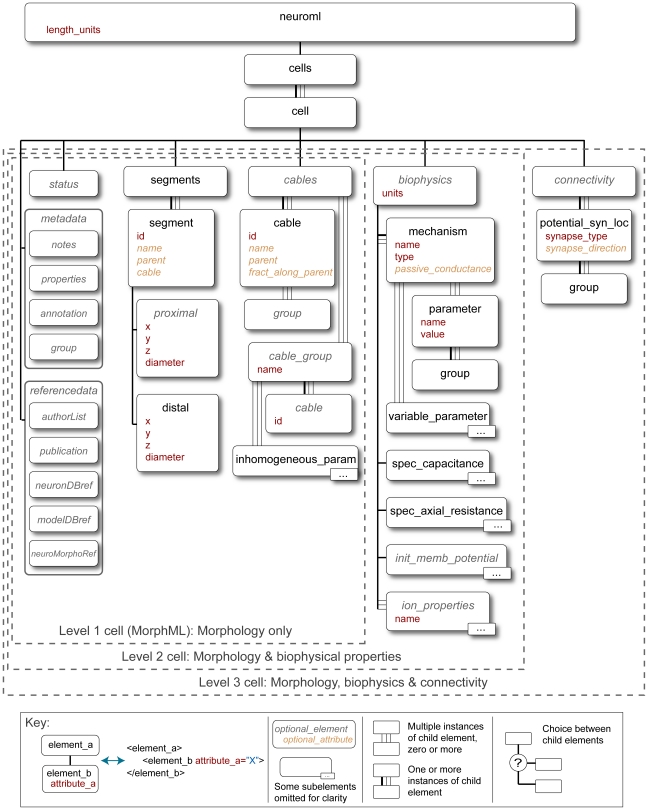
Elements for representing cells in NeuroML Levels 1-3. The main element for expressing a branching neuronal structure in NeuroML is *cell* which is used for all Levels in NeuroML. The core of the cell description is a set of *segment* elements which describe the 3D shape of the cell. These can be grouped into *cables* which represent unbranched neurites of the cell. Metadata present in the cell description can contain details of the creators of the cell model, or the data on which it was based (e.g. a neuronal reconstruction from NeuroMorpho.org). Addition of the *biophysics* element allows a Level 2 conductance based spiking cell model to be described, and the *connectivity* element can be used for the allowed synaptic connectivity of a Level 3 cell (e.g. to be used when connecting the cell in a network). A detailed description of each of these elements can be found in Supporting Text S1. Only the elements in Level 1 which are normally used in compartmental cell modeling are shown in the figure. Other elements such as *freePoints*, *features* etc. could be present in a Level 1 file from a camera lucida reconstruction [38].

The modular nature of the NeuroML language allows modelers to use only the components relevant for their system. This is enabled by using a number of XML Schema (XSD) files (see Materials and Methods) for each part of the language. The structure of the elements used to specify each component of the language is depicted in Figures 4–6. In the following sections we discuss each of the 3 Levels in more detail.

**Figure 5 pcbi-1000815-g005:**
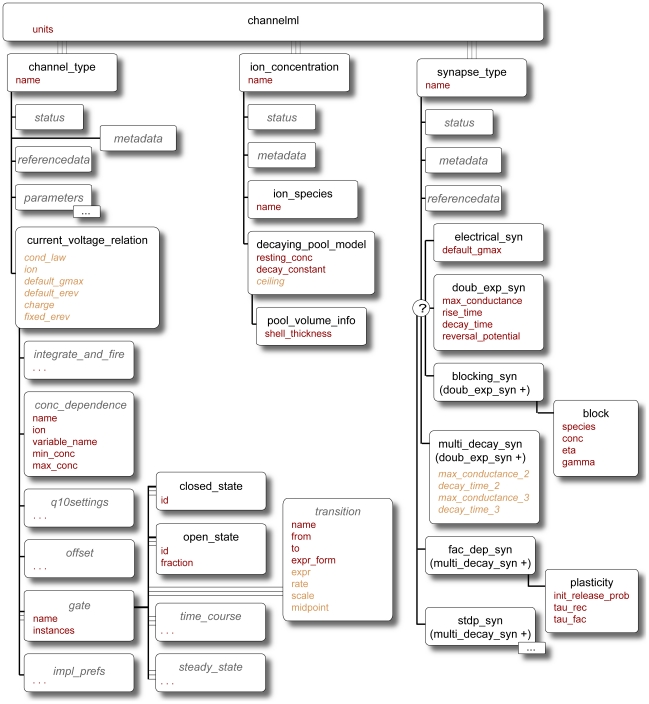
Elements in ChannelML. ChannelML allows expression of models of voltage (and ligand) gated conductances which are dispersed across the cell membrane (in *channel_type* element), conductances which are concentrated at synaptic contacts (in *synapse_type* element) and basic models of time varying internal ion concentrations (in *ion_concentration* element). Distributed conductance descriptions contain a number of *gate* elements, which describe the transitions between conducting and non conducting states of the channels underlying the conductances. A number of synaptic conductance models are allowed including simple double exponential waveforms, AMPA and NMDA receptor mediated synapses, Short Term Plasticity (STP) models, Spike Timing Dependent Plasticity (STDP) models, and electrical synapses. The *ion_concentration* element can be used for the simple models of exponentially decaying Ca^2+^ pools often used in detailed cell models. A detailed description of each of these elements can be found in Supporting Text S1.

**Figure 6:**
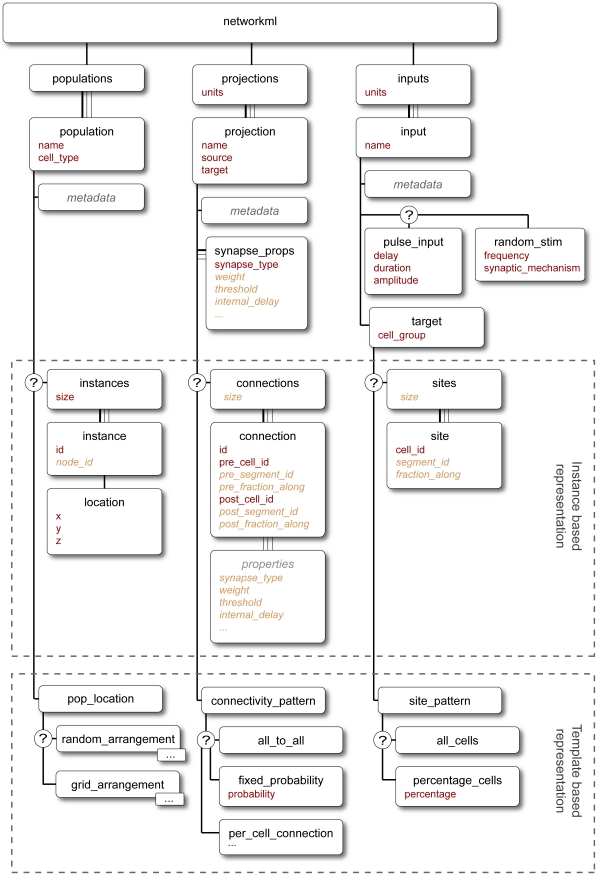
Elements in NetworkML. The core elements for expressing networks are *population* for homogenous groups of cells positioned in 3D, *projections* for synaptic contacts between (or within) populations and *inputs* for electrical stimulation to the network. The networks can either be expressed as lists of precise positions, connections and input locations (instance based representation) or as templates for generating these lists (template based representation). A detailed description of each of these elements can be found in Supporting Text S1.

### NeuroML Level 1

The first Level of the NeuroML language has two main purposes: to define neuronal morphologies (MorphML) and metadata, which provides additional information about model components at this and subsequent levels. Cells are described by lists of *segment* elements, with each element containing the 3D location and shape of each segment. Details of the mapping between elements in MorphML and the data structures of other applications that use morphology formats such as Neurolucida, NEURON and GENESIS have previously been described [38], and the elements permitted for a cell description at this and subsequent Levels is shown in Figure 4 (a detailed description of each of these elements is given in Supporting Text S1). Manual reconstruction of complex neuronal morphologies is a difficult and time consuming task and human errors can be difficult to detect. Once converted to MorphML, the morphology files can be automatically checked for discontinuities and isolated elements. MorphML also allows description of other anatomical information, which may have been recorded during cell reconstruction, such as histological features, reference points, and outlines of perceived boundaries [38].

NeuroML Level 1 also allows metadata, which is important for tracking the provenance of the model components and for providing background information on the model. A number of elements are included to provide structured information on the original authors of the model, translators of the model to NeuroML format, publications, and references to entries in databases such as ModelDB [43] and NeuroMorpho.org [44], as well as text based comments. The concept of model stability (the *status* element) is also included to allow a record of any known limitations of the model. Two types of unit system are allowed in NeuroML, SI Units and Physiological Units (ms, mV, cm, etc.), and only one of these must be used consistently in relevant elements of a NeuroML file. This facilitates the correct conversion of physical quantities to the unit system of each supported application.

### NeuroML Level 2

The second Level of the NeuroML language describes the electrical properties of the membrane that underlie rapid signaling in the brain. The two main parts of this Level are: an extension of the morphological descriptions from Level 1 that includes details of the passive electrical properties and channel densities on various parts of the cell (Level 2 cell in Figure 4); and ChannelML, which allows descriptions of the individual conductance mechanisms (Figure 5). ChannelML supports two main types of conductances: those that arise from channels distributed over the plasma membrane (*channel_type* element), such as voltage-gated conductances or conductances gated by intracellular ions (e.g. [Ca^2+^] dependent potassium conductances); and conductances arising at synaptic contacts (*synapse_type*). Distributed conductances are normally specified by describing the transition rates between channel states and their voltage dependence (Figure 3; Supporting Text S1). This allows specification of channel gating models with the traditional Hodgkin-Huxley formalism (with multiple instances of identical gates; e.g. Figure 3A) or with more detailed state-based kinetic (Markov) models (of which the HH model is a special case). A wide range of examples of voltage-gated conductances are supported by ChannelML including those underlying fast and persistent Na^+^ currents, delayed rectifier, A- and M-type K^+^ currents, H-currents and L- and T-type Ca^2+^ currents. [Ca^2+^] dependent BK and SK type channels can also be expressed. The commonly used Q_10_ function for temperature dependence of transition rates can be added. While the focus of NeuroML to date has been on more detailed conductance based models, ChannelML also supports a basic integrate-and-fire neuron model. However, more advanced types of reduced model such as exponential integrate and fire or Izhikevich spiking neurons are not yet supported (see Discussion for future plans for support of more abstract neuronal representations).

Both neurotransmitter gated conductances at chemical synapses and gap junction conductances at electrical synapses are supported in ChannelML (Figure 5). Conductance changes at chemical synapses are defined by a time course which can have a number of forms including an exponential rise and up to three decay components. These conductances include both the simple linear ohmic type (for modeling most AMPA and GABA_A_ receptor mediated synapses) and non-linear voltage-dependent components (for modeling the Mg^2+^ block of the NMDA receptor mediated synaptic component). Activity dependent synaptic plasticity is implemented with two mechanisms in ChannelML: a short-term plasticity (STP) mechanism based on a widely used STP model [45] incorporating both depression and facilitation components and a spike timing dependent plasticity (STDP) mechanism based on the model of Song and Abbott [46], but simulator support for STDP is presently limited. NeuroML provides representations of phenomenological models of synaptic plasticity that can reproduce a wide range of behavior including short-term facilitation and depression and Hebbian and anti-Hebbian learning, thus accommodating synaptic plasticity over a wide range of time scales where adequate simulator support exists.

Level 2 also allows the location and density of membrane conductances described in ChannelML to be specified on regions of the cell (e.g. soma, axon, apical dendrites). The passive electrical properties of the cell (e.g. specific axial resistance and specific membrane capacitance) can be defined in a similar manner (using the *biophysics* element; see Figure 4). Moreover, non-uniform channel densities can be implemented using a metric, such as the path distance from soma, and expressing the density in terms of this metric (using the *variable_parameter* element). Although NeuroML Level 2 is required for defining a full spiking neuron model, elements of the models can be defined as standalone descriptions in MorphML and ChannelML, thereby facilitating the exchange and reuse of individual model components.

### NeuroML Level 3

The third Level of NeuroML allows specification of the 3D anatomical structure and synaptic connectivity of a network of neurons, together with the properties of the external input used to drive the network. NeuroML Level 3 has two main purposes: to define NetworkML (Figure 6) and to allow extension of Level 2 cells with specification of regions of the cell membrane (e.g. apical dendrites) to which specific synaptic connections are limited (*connectivity* element; Figure 4). Thus complex networks with different types of excitatory and inhibitory neurons can be defined, including dendritic sub-region specific synaptic connections. There are two possible ways to describe networks in NetworkML: an explicit list of instances of cell positions and synaptic connections (instance based representation); or as an algorithmic template for describing how instances of the network should be generated, for example to place 300 cells randomly in a certain 3D region (template based representation). The instance based representation is quite compact, even for large scale simulations, because a network with 10,000 identical neurons will only have one instance of the cell description and a list of 10,000 locations. To date, this has proven a more useful and portable format. Only a limited range of network templates is currently supported, though these are in the process of being updated for the next version of NeuroML (see Discussion). The instance based representation can also include information on the computational node a cell should be run on (*node_id* attribute) to facilitate execution of large scale networks on parallel computing hardware (Supporting Text S1).

There are three core elements for describing networks in NetworkML: *population* specifies the numbers of cells of a specific type, together with their locations in 3D space; *projection* defines the set of synaptic connections between two populations or within a single population, by identifying the precise location of the synapse on the pre- and postsynaptic neuronal morphology and specifying the type of synapse(s) present; and *input* describes an external electrical input into the network. Inputs can take the form of a current pulse delivered by model electrodes or random synaptic stimulation.

### Simulator support and conversion of NeuroML to textual and graphical formats

The key goals of the NeuroML initiative are to make models and their components exchangeable, simulator independent and accessible to a wide range of researchers. To this end several software applications for compartmental modeling have been extended to support NeuroML. The most extensive support for NeuroML at present is provided by neuroConstruct which is an application for building, visualizing and analyzing networks of compartmental neurons in 3D space [39]. It uses an internal representation for cells that is closely related to NeuroML and can import and export model components in MorphML, ChannelML and NetworkML (in XML or a more compact HDF5-based binary format) or a complete description of a network model in a single Level 3 file. There is support for plotting channel properties (e.g. voltage dependence of rates; Figure 3C) and analyzing properties of neuronal morphologies and networks. Simulator specific scripts for NEURON, GENESIS, MOOSE, PSICS and PyNN can also be automatically generated from NeuroML files and executed, and simulation results can be reloaded for visualization and analysis (Figure 2).

NEURON allows native import and export of cells in both Level 1 and 2 NeuroML formats [47]. This allows cell models created in NEURON native scripts to be exported in a standardized format. All channel types currently in ChannelML can be converted to NEURON due to the flexible nature of the NMODL language [48]. GENESIS 2 [26] does not natively support NeuroML, but a mapping to this format is provided via neuroConstruct. MOOSE (Multiscale Object-Oriented Simulation Environment) [27] has been developed as part of the GENESIS 3 initiative, but is based on a complete reimplementation of the core of GENESIS. Scripts specifically for MOOSE can be generated by neuroConstruct and are for the most part identical to GENESIS 2 scripts, and native support for NeuroML in MOOSE is in development. NeuroML mappings have also been created for the recently developed PSICS simulator, and scripts for running single cell models on this simulator can be generated through neuroConstruct. There is also some native support in PSICS for importing MorphML and ChannelML. PyNN [49] is a Python package for creating network models for multiple simulators (including NEST [28] and NEURON), and support for mappings to and from NeuroML has recently been added. Table 1 summarizes the current support in each of the aforementioned tools for various types of models which can be expressed in NeuroML. In addition to the applications mentioned here, native support for various parts of NeuroML is currently in development in software applications not associated with the authors of this paper, including CX3D [50] and PCSIM [51]. NeuroML support is in development for Neurospaces [52], also being developed as part of the GENESIS 3 initiative. The latest details of software support for NeuroML can be found at http://www.neuroml.org/tool_support.

**Table 1:** Summary of supported NeuroML features in applications.

	NEURON	GENESIS	MOOSE	PSICS	neuroConstruct	PyNN*
Single compartment cells	X	X	X	X	X	X
Multi compartment cells	X	X	X	X	X	
Integrate & fire mechanisms	X				X	X
HH channels	X	X	X	X	X	
Kinetic scheme channels	X			X	X	
Voltage & ligand gated channel, e.g. BK, SK	X	X	X		X	
Networks	X	X	X		X	X
Static synapses	X	X	X		X	X
Plastic synapses	X				X	X
Gap junctions	X	X	X		X	

The latest support for NeuroML in these and other computational neuroscience tools can be found at http://www.neuroml.org/tool_support.

*Simulator mappings of PyNN which have been tested to date: NEURON, NEST.

To help researchers convert their existing models to NeuroML, we have generated a number of sample documents on the NeuroML website (http://www.neuroml.org/examples), which can be viewed in the original XML or converted to more readable formats (e.g. Figure 3B). There is also a software application for validating NeuroML files to check that they are compliant. MorphML cells and NetworkML files can be converted for visualizing in 3D in a web browser using an X3D compatible plug-in. Moreover, MorphML and ChannelML files can be converted online to a number of simulator formats including NEURON, GENESIS/MOOSE and PSICS, using the XML Stylesheet (XSL) based mapping files which have been developed for each simulator (Materials and Methods). In order to test the mappings from NeuroML to these simulators and other tools, we have converted a number of existing, published models to NeuroML.

### Validation of NeuroML

**Figure 7:**
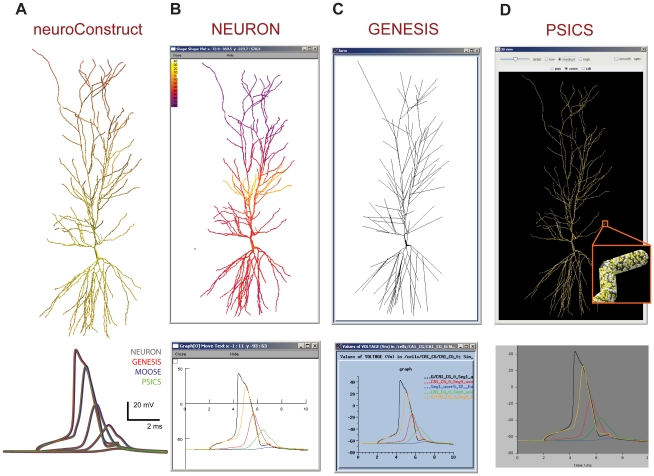
CA1 pyramidal cell model with non-uniform active conductances (based on Migliore et al.[2]). (A) Top: cell morphology visualized in neuroConstruct with color scale showing the density of h-type (HCN) channels (yellow lower, red higher). Bottom: voltage traces (in response to a current pulse input at the soma) at 5 different locations in the cell after execution on NEURON (gray), GENESIS (red), MOOSE (blue) and PSICS (green). (B) Voltage map of same cell executed on the NEURON simulator (top) and membrane potential traces (bottom) for the axon (black), soma (yellow) and 3 locations (green, blue, red) at increasing distances along the dendritic tree. (C) Recompartmentalized morphology visualized and run in GENESIS (top) with membrane potential traces (bottom, colors as for panel (B)). (D) Cell morphology visualized in PSICS using the ICING application (http://psics.org/icing, top). Inset shows a small section of dendrite and the locations of the individual ion channels. Membrane potential traces obtained with PSICS below, with colors as for panel (B). MOOSE does not have a native graphical interface at present. The simulation time step in all cases was 0.002 ms, and spatial discretisation is described in Materials and Methods.

To test that NeuroML descriptions of cell morphology and conductances can produce similar results across supported simulators, we converted a morphologically detailed model of a CA1 pyramidal cell [2] with 6 active conductances from the original NEURON format into NeuroML and compared the model behavior on NEURON, GENESIS, MOOSE and PSICS. This model was chosen because it contains three conductances that are non-uniformly distributed over the dendritic tree. The behavior of the ChannelML representation of the 6 conductances was first verified using a single compartment cell (Supporting Figure S1). The detailed 3D cell and its response to a brief current injection in the soma are shown in Figure 7. The time courses of the membrane potential at various points along the cell was directly compared for the four simulators (Figure 7A). Despite important differences in the way each simulator handles the simulation of the cell anatomy and channels (e.g. the morphology was mapped to a reduced number of compartments on GENESIS/MOOSE, and the numbers of ion channels and their individual positions were explicitly calculated in PSICS; Materials and Methods), the physiologically measurable output of the cell was very similar across all simulators tested (Figure 7B–D) confirming the simulator-independence of the NeuroML model description on short timescales and for a realistic neuronal morphology.

**Figure 8:**
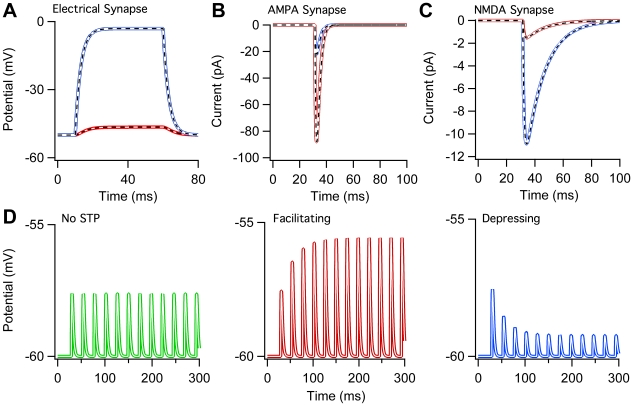
Models of electrical and chemical synapses implemented in NeuroML. (A) Voltage traces from a pair of gap junction coupled model cells (300 pS) during 0.19 nA current pulse injected into one of the cells. Blue indicates cell receiving current pulse and red shows gap junction coupled cell simulated in GENESIS. White overlapping dashes indicate the same model in NEURON. Black overlapping dashes indicate the same model in MOOSE. (B) Simulated EPSCs for a single compartment cell receiving synaptic input through an AMPA receptor only synapse at a membrane potential of −80 mV (red) and −20 mV (blue) in GENESIS. Again, the dashed lines indicate the equivalent NEURON (white) and MOOSE (black) simulations. (C) As B but for a single compartment cell receiving synaptic input through an NMDA receptor only synapse. (D) Short–term plasticity (STP) model [45]: membrane potential of a postsynaptic cell receiving a regular presynaptic spike train for a synaptic connection exhibiting no STP (green, left), facilitation (red, middle) and depression (blue, right) implemented on the NEST (colored) and NEURON (white overlap) simulators.

To test the synaptic models defined in NeuroML, we compared the behavior of a number of supported models between simulators. The ChannelML implementation of an electrical synapse was tested by comparing simulations run on GENESIS, MOOSE and NEURON. The voltage responses in a pair of passive model neurons connected by a gap junction to a step current injected into one of the cells gave rise to identical results in these simulators (Figure 8A). Neurotransmission at excitatory chemical synapses is mediated predominantly by glutamate in the mammalian brain. Glutamate typically activates AMPA receptors (Figure 8B), which have a simple ohmic conductance and NMDA receptors, which exhibit a nonlinear voltage dependent conductance due to Mg^2+^ block (Figure 8C). In all cases the results from NEURON, GENESIS and MOOSE match for simulations derived from the ChannelML description. The ChannelML implementation of a synaptic Short Term Plasticity (STP) model [45] was also compared using NEST and NEURON. Altering the model parameters to favor short-term depression or facilitation gave identical results (Figure 8D) using both simulators.

**Figure 9:**
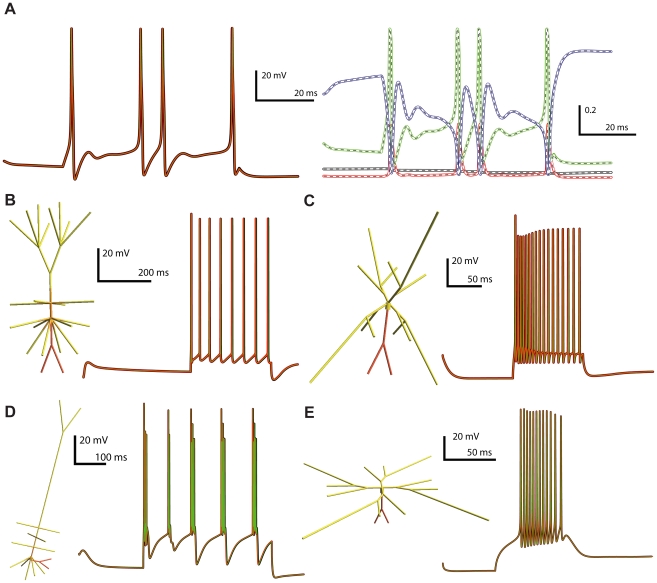
Comparison of the behavior of NeuroML-based cortical and thalamic cell models run on NEURON, GENESIS and MOOSE simulators. (A) Single compartment cell model containing all 22 active conductances present in the detailed cell models (Supporting Table S2), together with a passive conductance and a decaying calcium pool. Left plot shows the membrane potential response to a 80 pA current injection on NEURON (black), GENESIS (red) and MOOSE (green). Right plot shows the behavior on NEURON of the activation variables for the anomalous rectifier (thick black line), L-type Ca^2+^ (red) and persistent Na^+^ conductances (green) and the inactivation variable of the fast Na^+^ conductance (blue). White curve overlays show the corresponding GENESIS traces, and dashed lines show MOOSE traces. (B–E) 3D representations of four cell models from Traub et al. [15] implemented in NeuroML, color indicates the density of fast sodium conductances on the cell membrane (red: high - yellow: low). Graphs show somatic membrane potential during current injections for: (B) regular spiking (RS) Layer 2/3 pyramidal cell; (C) superficial low threshold spiking (LTS) interneuron; (D) intrinsically bursting (IB) Layer 5 pyramidal cell; (E) nucleus reticularis thalami (nRT) cell (trace colors as for left panel of A). See Supporting Figure S3 for further details of these and the 6 other electrically distinct thalamic and cortical cell models converted to NeuroML.

To test the support for network representations in NeuroML, we converted the elements of the thalamocortical column network model developed by Traub et al. [15] to NeuroML, as this is one of the most advanced multi-cellular network models published to date. The electrical behavior of the model arises from 22 voltage- and ligand-gated Na^+^, K^+^ and Ca^2+^ conductances together with both electrical and chemical synapses, which were all converted to ChannelML and tested (Figure 9A, Materials and Methods). Each of the 14 cell types present was converted to NeuroML, using the Level 2 cell export function of NEURON and import function of neuroConstruct (Supporting Figure S2). Supporting Tables S1 and S2 list the cell and channel types respectively. The different complements of the channels and different morphologies gave rise to a variety of behaviors including regular spiking, fast spiking and bursting behavior (Figure 9B–E). The NeuroML implementation produced qualitatively similar spiking behavior for simulations run in NEURON, GENESIS and MOOSE in the 10 electrophysiologically distinct cells during sustained firing over hundreds of milliseconds to seconds (Supporting Figure S3). However, differences in the timing of spikes was evident in some of the cells, unless the spatial and temporal discretisation of the cell was increased substantially. Two observations confirmed that the main cause of divergence in spike times arose from the use of symmetrical compartments (where axial resistance is split and numerical integration takes place at the center of the compartment) and asymmetrical compartments (axial resistance is located at one end of the compartment). Firstly, the spike times of a single compartment cell with all the channel conductances included were indistinguishable on NEURON, GENESIS and MOOSE (Figure 9A), confirming the ChannelML implementations allowed equivalent behavior on all 3 simulators. Secondly, when the spatial discretisation of the cell models was increased, all simulators tended toward the same spike times (Supporting Figure S4), with GENESIS (for which asymmetrical compartments had to be used, see Materials and Methods) generally requiring a finer discretisation. These results show that the way models are implemented on different simulators can have a significant impact on their behavior. Moreover, true interoperability, as measured through model convergence, may only occur at the limits of spatial and temporal discretisation.

**Figure 10:**
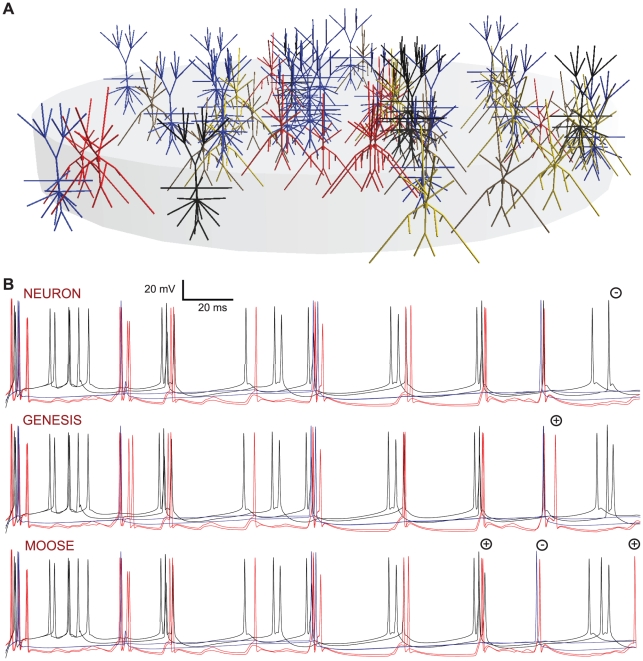
Comparison of the behavior of a NeuroML-based Layer 2/3 network model with 5 cell types connected with both electrical and chemical synaptic connections run on NEURON, GENESIS and MOOSE simulators. The network is based on the larger network described in Cunningham et al. [16], and uses five of the cortical cell models converted to NeuroML from Traub et al. [15]. (A) 20 regular spiking pyramidal cells (RS, blue), 6 fast rhythmic bursting pyramidal cells (FRB, black), 10 low threshold spiking interneurons (LTS, red), 10 axo-axonic interneurons (yellow) and 10 basket cells (brown) placed at random in a cylindrical region. The network contained electrical connections between the cells within each population, along with 4300 excitatory connections of 10 types within and between populations and 3800 inhibitory connections of 12 types (Supporting Table S4), but these are not shown. (B) Somatic membrane potential traces from 2 each of RS, FRB and LTS cells (with colors as in (A)) for simulations run on NEURON (top), GENESIS (middle) and MOOSE (bottom). Simulation time step was 0.001 ms.

Once all the channel, synaptic and cellular components of the model were converted to NeuroML and tested, we used neuroConstruct [39] to build a 56 cell Layer 2/3 network that matched as closely as possible a previous larger scale model which uses these cells [16]. This consisted of regular spiking and fast rhythmic bursting pyramidal cells and low threshold spiking, axo-axonic and basket type interneurons (Figure 10A, Materials and Methods). As specified in the original model, excitatory and inhibitory synaptic conductances were located on specific dendritic and somatic segments and electrical synapses were included within cell populations. This network model was not tuned against any new experimental data and is primarily intended as a test case for comparison of network behavior across simulators. The spike times of the neuronal populations were similar across the 3 simulators over the first 200 ms of the simulation, when a small simulation timestep and fine spatial discretisation was used (Figure 10B). At longer times, some spikes became shifted and others appeared or disappeared depending on the simulator. This divergence in model behavior occurred earlier in the simulation run and was much more pronounced when a more typical time step and coarser discretisation was used (Supporting Figure S5), suggesting that in practice, the precise spike times, and even the occurrence of some spikes produced by complex network models, will depend on the simulator implementation. A complete description of this network model including cell structure, channels, synapses, and lists of cell locations and connections can be represented in a single Level 3 NeuroML file.

## Discussion

### Summary

We have developed, implemented and tested NeuroML, a simulator-independent neuronal model description language for defining data-driven models of neurons and networks with a high degree of biological detail. This XML based language has a modular structure and the current version is sufficiently advanced to allow the description of the complex branching structures of dendritic trees and axonal projections, their biophysical properties, voltage- and calcium-gated ion channels, chemical synapses with short-term synaptic plasticity, electrical synapses, and both large and small scale network structure. The implementation and interoperability of models expressed in NeuroML were tested and the functionality illustrated by expressing existing single neuron and network models of different brain regions in this format and by demonstrating equivalent model behavior on different simulators.

### Model interoperability, validation and reuse

Providing a structured, declarative framework for describing detailed neuronal models that is independent of any particular simulator implementation has a number of important benefits. Firstly, the behavioral properties of a model specified in NeuroML can be compared across simulators. This is important for testing the validity of results from a model, since all conclusions should be simulator-independent. Model comparison also aids bug identification, tests the robustness of a particular model implementation, highlights performance bottlenecks and promotes collaboration between different simulator communities. Secondly, describing model components with structured schemas written in XML facilitates machine automated validation of particular components (e.g. the integrity of a complex neuronal morphology defined in MorphML). Thirdly, the modular structure of NeuroML, and the standalone nature of many of the mechanisms, facilitates reuse of model components. This speeds up the construction of models and allows models with increasing biological detail to be built from previously developed components. This is important because detailed conductance-based neuronal models are labor intensive to develop, often taking years to go from initial experiments to published model. Enabling interoperability will accelerate the rate of progress by allowing investigators to use and extend previous work, rather than ‘reinventing the wheel’ each time they want to build a new model. Such models will also provide a ready-made resource for developing and testing new software tools in this area.

Model components defined in NeuroML can be automatically transformed into textual and graphical formats familiar to neurophysiologists (Figure 3B, C). This allows access to the mechanisms and parameters underlying the model for researchers unfamiliar with simulator scripting languages. Moreover, NeuroML compliant tools with user friendly graphical user interfaces, such as neuroConstruct, allow neuronal and network models to be visualized, modified and run without the need to write code. This increased accessibility and transparency also allows critical evaluation by a wider range of neuroscientists including both theoreticians and experimentalists. Publicly exposing the details of a model implementation will discourage poor practices, improving the quality and robustness of models. By providing a common language for simulators and tools to interact, NeuroML can help reduce the barriers between computational and experimental neuroscience, thereby encouraging wider use of such detailed models.

### Practical aspects of using NeuroML and limits to interoperability

Translation of an existing model to NeuroML can be achieved using the export function of one of the supporting applications, but this normally requires a detailed knowledge of the scripting language of at least one simulator. As tool support for the language increases, the goal is that the handling of XML will happen “behind the scenes”, as is the case in many SBML compliant applications. At the moment however some manual editing is usually required, especially for ChannelML files. Import and export of NeuroML for supporting simulators is currently “lossy” because not all simulators use all of the information available (e.g. information that a group of cables represents “the axon” is not retained on import into most simulators). For these reasons, NeuroML should currently be considered less a format for creating a new cell model from scratch and more as a format for the storage of stable models and components that are being made available for wider usage.

Ultimately there are limits to model interoperability. At the coarsest level, not all simulators can run all models because they are often designed for a particular application. For example, NEST has mainly focused on integrate-and-fire neuronal models and PSICS can presently only run single cell models. At a finer level of detail, the way in which a simulator represents a feature of the model may also be fundamentally different. In PSICS the location of individual ion channels is defined explicitly, whereas other simulators simply define conductance densities associated with each electrical compartment. Our results show that a key reason why the spike times of some multicompartmental cell models can diverge between simulators is the different way they treat neuronal morphology and the different locations at which the voltage is computed within each compartment. While increasing spatial discretisation and decreasing time step lead to model convergence (Supporting Figure S4, Figure 10), for some cells this only occurred in computationally inefficient regimes. Such direct comparison of the performance of different simulators will allow the most efficient solution to be identified, potentially improving overall simulator implementations. We have used basic measures of model performance such as spike times to assay convergence of model behavior. However, more sophisticated measures will need to be developed to ensure that the accuracy of the interoperability achieved is sufficient for modeling a particular biological system.

NeuroML has reached a state of maturity where it can be used to specify a wide range of published single neuron and network models. However, there are a number of features of the nervous system that have not been covered in current schemas because our initial priority was to focus on ensuring that NeuroML was backwards compatible with most types of compartmental models developed to date. Also, support for the wide array of simplified abstract neuron models currently in use is limited. Moreover, since a major requirement for NeuroML was interoperability between existing conductance based simulators, functions that only operate on a specific simulator or that would have to be developed de novo, were not the primary concern. Nevertheless, the horizontal, modular design of NeuroML makes it easier to add extensions for new mechanisms that cannot be described by current schemas.

While there is sufficient flexibility within the current language for creating detailed neuronal models of different brain regions with a wide diversity of custom channel types, morphological features and network connectivity, some researchers may wish to build on the basic NeuroML elements to encode features of their models not present in the core language. Options currently available for this include adding application specific information in metadata elements (e.g. annotating models with visualization information or adding a proprietary subclass of an element, such as a voltage dependent gap junction), creating hybrid models partially described in NeuroML and partially in native simulator script (e.g. channels in both ChannelML and NMODL format), and creating a domain specific XML Schema, which includes NeuroML for neuronal elements (e.g. a language for describing brain regions including vasculature which reuses the NeuroML schemas). These options are discussed in more detail at http://www.neuroml.org/NeuroMLValidator/Extending.jsp. These extension mechanisms enable greater flexibility than the current version of NeuroML affords and provides a bridge to new functionality before it can be formally incorporated into a new version of the language.

### Relationship of NeuroML to other standardization initiatives and databases

There are a number of initiatives to standardize model descriptions and data formats in biology. One of the most successful of these is the development of XML-based structured formats for describing models in systems biology [30], [31]. The acceptance of these formats as standards has been promoted by several journals (e.g. Nature Molecular Systems Biology), which encourage that model scripts be made available in SBML or CellML. NeuroML differs from these low level model description languages in that it implicitly contains high level concepts such as neuronal morphologies, synapses and network connections. This is necessary because NeuroML covers models that span many levels of description of the nervous system. While this is ultimately less flexible than CellML and SBML which describe models explicitly in terms of the mathematical expressions for its interacting components, it still allows a wide range of new models to be described including kinetic models of ion channels, synaptic models with distinct kinetic behavior, neuronal models with different ion channel complements and morphologies and networks with different anatomical connectivity.

In neuroscience, where models and data formats are highly diverse, there are a number of initiatives to improve interoperability and accessibility. BrainML (http://www.brainml.org) is also a structured XML-based language, but BrainML is principally designed for exchanging neuroscience data rather than defining neuronal and network models. The recent addition of Python based scripting interfaces to a number of simulators such as NEST [53], NEURON [47], MOOSE [27], and Brian [54] is a promising step towards greater model interoperability and has lead to the development of PyNN [49], a Python package for simulator-independent specification of neuronal network models. Instead of developing a declarative language for describing models as in NeuroML, this approach defines a Python application programming interface (API) to build models in a procedural manner. Scripts in this format can be used on multiple existing simulators. NeuroML is complementary to PyNN and cooperation between these initiatives aims to allow the creation of networks of NeuroML Level 2 cells by PyNN scripts and to extend the template based NetworkML descriptions so that they are compatible with PyNN.

### The future of NeuroML

The future success of NeuroML depends on its adoption by the community and the willingness of simulator developers to make their software NeuroML compliant. NeuroML development is Open Source, allowing all interested parties to contribute to the language. We are presently gathering requirements and specifications from experimentalists, software developers and theoreticians for future developments of NeuroML, which will increase the scope of the language, and permit more complex neuronal models to be specified, and we are actively engaged with standardization initiatives of the International Neuroinformatics Coordinating Facility (INCF). It is envisaged that NeuroML will evolve gradually from the present structure, which reflects a pragmatic functional solution to the interoperability problem, into a more generic flexible format, that links into other standards (e.g. SBML, CellML and MathML) and that is less homologous to implementations in existing simulators. For example, greater support for model components in SBML/CellML would enable more sophisticated synaptic transmission and plasticity models, pharmacological perturbation of cell behavior or creation of more abstract cell models. Backward compatibility of any future changes to NeuroML will be ensured through automated file conversion. We plan to increase the existing set of NeuroML based cell and network models for different brain regions, together with further development of a range of compliant applications for searching for, building, visualizing, simulating and analyzing the models, thereby providing theoreticians and experimentalists with a powerful toolbox for addressing fundamental questions about brain function.

## Materials and Methods

### XML

XML [40] is commonly used for data exchange between software applications and a number of generic tools and technologies have been developed to handle these types of files. The structure of the data an application can expect in an XML file can be defined using an XML Schema (XSD) file, and XML files can be automatically checked for validity against this schema before use. A number of such files are used to define the structure of NeuroML, and the modular nature of the language is enabled by having separate schema files for MorphML, ChannelML, etc. and importing as appropriate for each Level description. A detailed description of the contents of the NeuroML schemas is in Supporting Text S1.

There are a number of methods for transforming XML documents into formats suitable for different applications. One way is to create an XML Stylesheet (XSL) file, which allows transformation of the data in the XML file into another text file format. XSL files have been created for transformation of NeuroML files to HTML (e.g. Figure 3B) and a number of simulator specific formats (as are used to convert example files at http:/www.neuroml.org). This approach has the advantage that scripts in the native language of the simulator can be generated, which doesn't require changing the code of the simulator. Another method is to parse the XML files directly using the Document Object Model (DOM) approach where a treelike structure is built up in application memory of the contents of the file (suitable for smaller files, e.g. ChannelML descriptions) or the Simple API for XML (SAX) approach where the contents of a larger file (e.g. a NetworkML instance based description) is parsed sequentially from start to finish.

### CA1 pyramidal cell

The CA1 Pyramidal cell model (Figure 7) is based on a model used in (Migliore et al., 2005), and the NEURON scripts for this were obtained from the ModelDB repository (accession number 55035). The model was executed in NEURON and the ModelView tool was used to export the cell in NeuroML Level 2. Full export of the cell model including information on all channel densities was not possible, as the densities of a number of channels (those underlying the H current (hd) and proximal (kap) and distal (kad) A-type potassium currents) were adjusted with custom functions in file fig2A.hoc (densities varied as linear functions of distance from the soma). The information on the function to create these densities is lost once the cells are created in NEURON. The values of channel densities of hd, kap and kad at the centre of sections were included in the exported NeuroML file giving an approximation to the linear function. The cell also contained a passive conductance (pas), a delayed rectifier K^+^ (kdr) conductance and Na+ conductances on the axon (nax) and soma/dendrites (na3).

Once the Level 2 file was imported to neuroConstruct (cell CA1_imported in the project mentioned below) a copy of the cell was created (cell CA1), the exported channel densities for hd, kap and kad were removed, and Variable Mechanisms added for these channels specifying the changes in densities in terms of the distance from the soma. These Variable Mechanisms and the Parameterized Groups (e.g. PathLengthOverDendrites) have corresponding elements in NeuroML, so the cells can be imported and exported in NeuroML without loss of information. Also, NeuroML files from other sources can use non-uniform expressions for channel densities that will be preserved on import to neuroConstruct. The channel mechanisms were manually converted to ChannelML using neuroConstruct (see http://www.neuroconstruct.org/docs/importneuron.html). Using a single compartment cell with a passive conductance and each of the channels in turn and comparing the membrane potential and the state variables of the channels allowed validation of the representation of the channels in ChannelML (see Simulation Configuration OneChannelCells for an example). The default simulation configuration generates a single segment cell with all 6 channels and was used to validate the behavior of the channels, as illustrated in Figure S1.

The mappings of cell morphologies between MorphML, neuroConstruct, NEURON and GENESIS formats are discussed in detail in (Crook et al., 2007). In brief, a cell consists of cables (termed sections in neuroConstruct) containing one or more segments which provide the 3D points/diameters as obtained in the cell reconstruction. The CA1 cell in this example had 173 cables containing a total of 2243 segments. The NEURON mapping of the cell is straightforward, the cables/sections are mapped to NEURON sections, with segments providing the pt3d points along the sections. Associated with each section in neuroConstruct there is a number indicating the internal divisions to be used for spatial discretisation, which is mapped to NEURON's nseg variable.

In GENESIS and MOOSE, the basic entities from which cells are composed are cylindrical/spherical compartments with no internal divisions. As opposed to making a one to one mapping from each segment to a compartment, there is a function in neuroConstruct which maps sections (e.g. containing 20 segments with number internal divisions/nseg = 4) on to a set of compartments with an equivalent length, surface area and total axial resistance, based on this spatial discretisation (the example above would be mapped to 4 compartments with the radii chosen appropriately). More details on this conversion process can be found at: http://www.neuroconstruct.org/docs/Glossary_gen.html#Compartmentalisation. The script files for which are generated for MOOSE are almost identical to those for GENESIS, with some minor alterations to cater for differences in message handling, the different use of symmetric/asymmetric compartments between the simulators and the lack of native graphical interface support in MOOSE.

The representation of morphologies in PSICS is based on a list of points defining the cell structure, and there is no inbuilt concept of a cable. A PSICS file containing these points is generated by neuroConstruct from the cell's segment information. Points can have labels associated with them and these are used to associate the points with the groups present in the cell (soma_group, apical_dendrite, etc.), and these can be then used in the distribution of channels to various areas of the cell. While PSICS was designed to be able to investigate effects of the stochastic nature of channels, in this case the channels were modeled deterministically, to allow direct comparison with the noise free simulations of NEURON & GENESIS/MOOSE.

The Simulation Configuration in the neuroConstruct project to reproduce the traces in Figure 7 is called CA1Cell. The cell required a finer spatial discretisation than present in the original model (the sections in neuroConstruct had a total of 3008 internal divisions; these were mapped to NEURON sections with a total nseg of 3008, and mapped to 3090 GENESIS/MOOSE compartments). PSICS decides itself on the cell's compartmentalization based on a maximum value given to it for structural discretisation (in this case 1.3 µm) which resulted in 7821 compartments.

A zipped file containing all of the NeuroML elements used in this model and or a full neuroConstruct project (CA1PyramidalCell.ncx) which can be used to run the cell model on NEURON, GENESIS, MOOSE or PSICS are available at: http://www.neuroml.org/models. The simulations were carried out with NEURON version 6.2, GENESIS v2.3, MOOSE SVN revision 1473, PSICS v1.0.6, and neuroConstruct v1.3.4.

### Synaptic mechanisms

The NeuroML specification for the electrical synapse model was used to generate simulation scripts for NEURON, GENESIS and MOOSE through neuroConstruct. The neuroConstruct project (Ex9_Synapses.ncx, included with the standard download of neuroConstruct, or available here: http://www.neuroConstruct.org/samples) can be used to generate this example and a number of others including networks of multiple spiking neurons connected by gap junctions, for execution on the different simulators. All generated networks can also be exported to valid NeuroML Level 3 files.

The ChannelML implementations of AMPA and NMDA receptors were tested in NEURON, GENESIS and MOOSE using voltage clamp to simulate EPSCs (Figure 8B & C). In both cases a passive compartment having leak conductance of 3.0×10^−9^ mS µm^−2^ and capacitance 1×10^−8^ µF µm^−2^ received synaptic inputs whose strength and relative weight had been adjusted so as to match experimental voltage clamp data [8]. Synaptic input was then applied to the clamped cell at holding potentials of −20mV and −80mV and the clamp current was recorded. NeuroML representations of these synapse models are contained in the neuroConstruct project mentioned above.

To test STP (Short Term Plasticity) (Figure 8D) a spontaneously active presynaptic cell (integrate and fire neuron with leak conductance having a reversal potential just above spiking threshold) was connected to a postsynaptic integrate and fire cell by a conductance based STP synapse. The subthreshold response of the postsynaptic cell was then monitored as it received regular spikes from the pre-synaptic cell. To examine synaptic depression, the facilitation time constant tau_fac was set to 0 while the recovery time constant tau_rec was set to 120ms. To examine synaptic facilitation, tau_rec was set to 0 while tau_fac was set to 300ms. STP was turned off in the control case by setting the facilitation and recovery parameters both to 0. See Supporting Text S1 for a detailed description of the STP mechanism. The NeuroML specification of the model was converted into simulation scripts for PyNN using neuroConstruct. PyNN was then used to run the simulation in both NEURON and NEST, producing the similar traces as shown in Figure 8D. In the neuroConstruct project Ex8_PyNNDemo.ncx (available from http://www.neuroConstruct.org/samples; simulation configuration STPDemo), there are three postsynaptic cells all connected to the presynaptic cell. Each postsynaptic cell connects with one of: control synapse, facilitating synapse, depressing synapse. This allows all examples to executed simultaneously.

These examples were generated with NEURON v6.2, GENESIS v2.3, MOOSE SVN revision 1473, NEST v1.9.8017, neuroConstruct v1.3.4 and PyNN v0.5.0.

### Thalamocortical cell models

The full network model from [15] was originally developed in Fortran for execution in parallel on 14 CPUs. The cell models were converted from this to NEURON format together with the network connectivity. Both the original Fortran and NEURON scripts are available on ModelDB (accession number 45539). A discussion on the conversion of the model from Fortran to NEURON format is available at: http://senselab.med.yale.edu/ModelDB/ShowModel.asp?model=82894&file=\nrntraub\README.

The cells in NEURON format were taken as a starting point for the conversion to NeuroML. Supporting Table S1 lists the cell types present. Each of the cells has a set of voltage and ligand gated ion channels on its membrane, along with a passive conductance and an exponentially decaying pool of calcium. The full list of conductance types are given in Table S2. These channels were manually converted from NMODL format to ChannelML using neuroConstruct. The channels were individually tested to ensure that the ChannelML reproduced the behavior of the original mod file implementation and that the mappings to NEURON, GENESIS and MOOSE matched. To validate the simulator independence of a cell with multiple channels in this format, we tested a single compartment cell with all 22 of these of these active conductances the passive conductance and internal pool of calcium. Figure 9A shows the behavior of the membrane potential and a number of internal channel variables of the cell on NEURON, GENESIS and MOOSE. The timestep for the simulation was 0.005 ms. Note that neither this simple cell, nor any of the more detailed cells, can be run on PSICS as that simulator does not currently support ligand gated channels (e.g. kc, kahp).

The cells were exported from NEURON in NeuroML Level 2 format via ModelView and imported into neuroConstruct. The correct channel densities were included with the Level 2 file but the morphologies were generally of the format as seen in Figure S2A. This is due to there being no 3D positions information associated with the sections when they were created in NEURON (the original cortical column network model was 1D), and the representation below shows the points (in a 2D plane) NEURON automatically generated for the sections based on the connectivity and section lengths. In neuroConstruct a more representative 3D structure for each cell was created by rotating the sections of the imported cells, preserving the length and section groupings, and so keeping the electrical behavior of the cells the same (Figure S2B).

All 14 cell types were converted in this way and each can be visualized using the neuroConstruct project (Thalamocortical.ncx) mentioned below. Each cell has a number of Simulation Configurations associated with it showing the behavior of the cell, usually with a brief hyperpolarizing pulse followed by a depolarizing pulse, generally based on the traces of individual cell behavior in Appendix A of [15]. Traces of each of the electrically distinct cells are shown in Supporting Figure S3. The closeness of the traces between NEURON, GENESIS and MOOSE varied with simulation time step and spatial discretisation. We used a criterion that the same number of spikes should be present in each trace, and that the differences in spike timing should be no more than 0.5% of the simulation run time (e.g. spikes at 200 ms should not differ in timing by more than 1 ms). A smaller than normal timestep was required in all cases for convergence of cell behavior between NEURON, GENESIS and MOOSE. In general this meant a 0.005 ms or lower timestep, whereas 0.02 ms is often used for published models with these simulators. This lower than normal timestep has also been required with simpler cell models (e.g. single compartment granule cell model, Figure 4 in [39]).

A finer spatial discretisation was needed in all cells to minimize the inherent differences in the way the simulators handle morphological representations (NEURON uses cables/sections divided into nseg points for numerical integration, GENESIS can use symmetrical or asymmetrical cylindrical compartments, MOOSE uses symmetrical cylindrical compartments, see below). The spatial discretisation used in the simulations can be changed in neuroConstruct (by visualizing the cell, clicking on any segment, clicking the button Edit, and selecting Remesh in the drop down box, to choose a discretisation of each section which is constrained by a maximum electrotonic length, see http://www.neuroconstruct.org/docs/Glossary_gen.html#Electrotonic%20length). This value for the number of internal divisions for spatial discretisation in neuroConstruct is mapped to nseg in NEURON sections, and, as mentioned in the section above on the CA1 cell, a number of compartments are generated in GENESIS for these sections based on this value, thus setting the spatial discretisation in that simulator.

In GENESIS, there are two options for simulating compartments: symmetric compartments where the axial resistance is divided in two at each end of the compartment and the voltage is effectively calculated at the centre of the compartment, and asymmetrical compartments, where all of the axial resistance is at one side and which are slightly more efficient to use in simulations. Ideally for matching the behavior of NEURON and GENESIS, symmetrical compartments should be used, as NEURON also calculates the voltage at the centers of nseg regions of the section. However, in GENESIS v2.3 there is a known bug which doesn't allow use of the hsolve numerical integration method with symmetrical compartments when there are compartments with more than 2 child compartments (e.g. soma of pyramidal cell in Figure 9B). Use of hsolve is required for simulations as this is much faster than the basic Exponential Euler method. That method would require a much smaller dt (∼0.00001 ms) for convergence of the simulation. Therefore, in the simulations in Supporting Figure S3 (and in the network simulations in Figure 10), asymmetric compartments, together with hsolve are used. This necessitated a much finer spatial discretisation of the cells (leading to a longer run time for simulations). Plots showing the convergence of spike times for 3 different cells are shown in Figure S4, where it is clear that the symmetrical compartment simulations of NEURON and MOOSE converge with coarser discretisation than GENESIS simulations with asymmetric compartments.

The network used to illustrate a full Level 3 model in NeuroML is a scaled down version of the network model in [16]. Whereas that network contained 1440 cells, this version contained 56 cells. This was mainly due to the need for much finer spatial discretisation in the cells, a smaller timestep and the requirement that the simulator will run on a single processor in all three simulators. This network can be generated using simulation configuration CunninghamEtAl04_small in the associated neuroConstruct project (Thalamocortical.ncx). The names of the cell groups/populations are listed in Supporting Table S3. Synaptic mechanisms were added to the project based on the data in the supplementary information in [16]. Network connections were created through the neuroConstruct GUI based on the numbers of connections per cell in the original model. The total number of connections between each pair of cell groups in the model are listed in Supporting Table S4. The magnitude of the gap junction conductance was reduced by a factor of 0.7 (to 2.1 nS) to reduce the tendency of the scaled down network to become over synchronized. Input was added to the network in the form of continuous current injection of random amplitude to the FRB cells (0.15–0.25 nA), the LTS interneurons (0–0.2 nA), and the basket and Axoaxonic cells (0–0.02 nA). RS cells also received random pulse inputs of 0.4 ms duration and 0.4 nS amplitude on their axon at a mean rate of 1 Hz. However, for the 250 ms simulation run shown in Figures 10 and S5 no external pulses occurred.

The simulations were carried out with NEURON version 6.2 and GENESIS v2.3, MOOSE SVN revision 1473 and neuroConstruct v1.3.4. A zipped file containing the NeuroML elements used in this model and a neuroConstruct project (Thalamocortical.ncx) which can be used to generate and run the scripts for NEURON, GENESIS and MOOSE are available at: http://www.neuroml.org/models.

## Supporting Information

Text S1 Detailed description of all NeuroML elements
(0.55 MB PDF)

Figure S1 Single compartment cell with 6 channels from CA1 pyramidal cell on NEURON, GENESIS, MOOSE and PSICS
(0.08 MB PDF)

Figure S2 Layer 2/3 Fast Rhythmic Bursting Pyramidal cell as exported from NEURON and converted to 3D in neuroConstruct
(0.10 MB PDF)

Figure S3 Behavior of 10 cell models from Traub et al., 2005 on NEURON, GENESIS and MOOSE
(0.55 MB PDF)

Figure S4 Convergence of model behavior for fine spatial discretisation
(1.56 MB PDF)

Figure S5 Network model behavior with longer timestep and coarser spatial discretisation
(3.51 MB PDF)

Table S1 List of thalamocortical cell models from Traub et al., 2005
(0.01 MB PDF)

Table S2 List of conductances used in thalamocortical cell models from Traub et al., 2005
(0.01 MB PDF)

Table S3 List of cell populations in reduced Layer 2/3 network
(0.01 MB PDF)

Table S4 List of network connections in reduced Layer 2/3 network
(0.01 MB PDF)
