# Supplementary material for: NeuroML: A Language for Describing Data Driven Models of Neurons and Networks with a High Degree of Biological Detail
Source: PLoS Comput Biol. 2010 Jun 17;6(6):e1000815. doi: 10.1371/journal.pcbi.1000815 (PMC2887454; doi:10.1371/journal.pcbi.1000815)
Supplement: Text S1 — Detailed description of all NeuroML elements (0.55 MB PDF) [file pcbi.1000815.s001.pdf]

Supporting Text S1

NeuroML: Detailed description of language elements

Table of Contents

1. Overview 1
2. Namespaces and root elements 2
3. Note on formatting 2
4. Level 1: Metadata 3
5. Level 1: Cell descriptions (MorphML) 6
6. Level 2: Cell descriptions (MorphML & Biophysics) 9
7. Level 2: Channel & synaptic mechanisms (ChannelML) 11
8. Level 3: Cell descriptions (MorphML & Biophysics & Connectivity) 20
9. Level 3: Cell positions & connectivity (NetworkML) 21
10. Example NeuroML files 26
    - 10.1 Biophysical cell model 26
    - 10.2 Na^+ conductance in ChannelML 28
    - 10.3 Double exponential conductance waveform synapse 30
    - 10.4 Simple Network 31
11. References 33

1. Overview

This document describes in detail the main XML elements which are needed at the various Levels for describing specific neuronal entities in the NeuroML neuronal model description language. The elements are defined in XSD (XML Stylesheet Definition) Schema files. The W3C standard for XSD files is here: http://www.w3.org/XML/Schema, and a very good introduction to XML Schemas can be found here: http://www.w3schools.com/schema.

> Note: This document refers to v1.8.1 of the NeuroML specifications. It is intended that the document will also be updated as the specifications evolve (while the main NeuroML paper remains the primary description of the scope, goals and overall direction of the project). Check at http://www.NeuroML.org/specifications for the latest version of this detailed description.

The following links will always reference the latest release of the specifications:

MorphML: http://www.neuroml.org/NeuroMLValidator/Latest.jsp?spec=MorphML
ChannelML: http://www.neuroml.org/NeuroMLValidator/Latest.jsp?spec=ChannelML
NetworkML: http://www.neuroml.org/NeuroMLValidator/Latest.jsp?spec=NetworkML

Note also that for the very latest information on a particular element, the most recent version of the XSD Schema documents from the Subversion repository should be consulted. See https://sourceforge.net/svn/?group_id=136437.

The language is divided into 3 Levels. We will discuss these individually in detail. The corresponding XSD files for the Levels are as follows (the X is replaced by the version of the file, e.g. Metadata_v1.8.1.xsd):

Level	Filename	Purpose	Root XML element for valid files and default namespace
Level 1	**Metadata_vX.xsd**	Defines Metadata for use at all Levels	***- No standalone metadata files -***<br>http://morphml.org/metadata/schema
	**MorphML_vX.xsd**	Defines MorphML standalone morphology files, and histological features	**<morphml>**<br>http://morphml.org/morphml/schema
	**NeuroML_Level1_vX.xsd**	Links MorphML and Metadata for NeuroML Level 1	**<neuroml>**<br>http://morphml.org/neuroml/schema
Level 2	**ChannelML_vX.xsd**	Defines channel and synaptic mechanisms	**<channelml>**<br>http://morphml.org/channelml/schema
	**Biophysics_vX.xsd**	Defines biophysical properties of cells	***- No standalone biophysics files -***<br>http://morphml.org/biophysics/schema
	**NeuroML_Level2_vX.xsd**	Defines Level 2 cells and channels	**<neuroml>**<br>http://morphml.org/neuroml/schema
Level 3	**NetworkML_vX.xsd**	Defines positions, connections and electrical inputs for networks of cells	**<networkml>**<br>http://morphml.org/networkml/schema
	**NeuroML_Level3_vX.xsd**	Defines Level 3 cells and networks of those cells	**<neuroml>**<br>http://morphml.org/neuroml/schema

Table 1: NeuroML Schema files, root elements and namespaces

2. Namespaces and root elements

Namespaces are used in XML documents to allow the safe combination of elements from different sources in a single document. A general overview of namespaces in XML can be found here: http://www.w3schools.com/xml/xml_namespaces.asp.

The root element of an XML file will normally define the default namespace for the whole file, e.g. **<channelml xmlns="http://morphml.org/channelml/schema" … >** indicates that the elements in the file are from the ChannelML namespace by default. Elements from other namespaces can be included if the namespace qualifier is defined (at the root or any other element): **xmlns:meta="http://morphml.org/metadata/schema"** allows addition of elements in the metadata namespace: **<meta:notes>…</ meta:notes>**.

See http://www.neuroml.org/NeuroMLValidator/Samples.jsp for examples of NeuroML files at each Level. There are also a number of examples of complete NeuroML files presented in Section 10.

Note that **morphml.org** is used in the namespaces for historical reasons (since changing the namespace would make older files invalid), and will be replaced by **neuroml.org** in future versions of NeuroML.

3. Note on formatting

In this document words in **bold** will normally reference elements as they appear in NeuroML files, e.g. **segment**, **channel_type**. When the names start with a capital letter, e.g. **ChannelType**, this refers to the name used in the XSD Schema files for defining the type (e.g. simple type, complex type, etc.).

XSD Schema file:

```
<xs:element name="channelml" type="ChannelML">
       ...
</xs:element>

<xs:complexType name="ChannelML">
       ...
    <xs:element name="channel_type" type="ChannelType" minOccurs="0" maxOccurs="unbounded">
       ...
</xs:complexType>

<xs:complexType name="ChannelType">
       ...
</xs:complexType>
```

NeuroML file:

```
<channelml ... >
       ...
       <channel_type ... >
       ...
       </channel_type>

</channelml>
```

Attributes which are required are in **red bold text**, attributes which are optional are in **orange bold text** and values of attributes (most of which are defined as an enumeration of the only values permitted for the attribute) are in *italic text*. These conventions are used also in Figures 4, 5 and 6 in the main NeuroML paper.

4. Level 1: Metadata

This part of the specification defines a number of common elements which can be used in files at any of the Levels of NeuroML. These include defining useful simple types such as **NonNegativeDouble** and **Point3D** (see below for definitions) but also specifies the structured data associated with a NeuroML file which can be used to trace the source of the model data, authors, references, etc.

Group: **metadata**

This represents not a single XML element, but a group of associated elements (**notes**, **properties**, **annotation**, **group**) which can together be specified as subelements of other NeuroML element. Each of these elements is optional, and so **cell** and **channel_type** (and any of the other elements which have **metadata** as a subgroup) can use one or more of these as appropriate.

Element: **<notes>**

This element allows a simple string of text to be entered as a comment on a cell, channel or network description file, or at many of the subelements of such files. It is the minimum comment which should be added, but more structured data, such as publication references should be placed in the appropriate element.

Element: **<properties>**

This consists of a list of **<property>** elements, each of which has required **tag** and **value** attributes. This can be used for adding data with some structure, which isn't yet part of the specification, e.g. adding a URL referencing an entry in a lab's own database of morphological reconstructions or electrophysiological recordings.

Element: **<annotation>**

The type of this element is **any**, and it can be used to add any set of XML elements to a NeuroML file and for the file still to remain valid. One possible use of this is if an application wants to store metadata relevant only for that application within a NeuroML file, e.g. a graphical tool for constructing channel mechanisms might store information on the shape and screen layout of its depiction of the channel elements within a ChannelML file. This approach has been taken in a number of graphical SBML (Hucka et al., 2003) editors (e.g. CellDesigner) to separate out the "pure" SBML content from the tool's proprietary graphical information.

Element: **<group>**

This element is intended to allow grouping of elements within a file, e.g. groups of cables, and just contains a string naming the group.

Group: **referenceData**

This is another grouped set of elements (**authorList**, **publication**, **neuronDBref**, **modelDBref**, **neuroMorphoRef**), all of which are optional, but which can be used to provide valuable information on the authors of NeuroML files, associated publications and databases in which the original and/or latest version of the model elements can be found.

Element: **<authorList>**

This contains two subelements, **modelAuthor** and **modelTranslator**, and as the names suggest, it allows reference to the creators of the original model (normally in a simulator's native scripting language) and the persons who translated the model to NeuroML. Each of these elements is of type **Person**, an element which includes the subelements **name** (required), **institution**, **email**, **comment** (all optional).

Element: **<publication>**

An element to include information on publications related to the model. The **fullTitle** element should be used for a readable reference to the publication including at least authors, title, year and journal name (which can be used in the HTML view of the file), while the **pubmedRef** element should contain a URL to the document on PubMed. There is also an optional **comment** element to give an explanation on how the publication relates to the model, should it not be made clear elsewhere.

Element: **<neuronDBref>**, **<modelDBref>**, **<neuroMorphoRef>**

These elements allow a NeuroML file to contain references to entries on cellular information in NeuronDB (http://senselab.med.yale.edu/neurondb), models archived in ModelDB (http://senselab.med.yale.edu/modeldb) or a neuronal reconstruction in the NeuroMorpho.org database, respectively. All have subelements allowing a name/reference for the element in the respective repositories (**modelName**), a reference to the relevant web resource (**uri**), and an optional **comment** element to give an explanation on how the entry relates to this model.

Element: **<status>**

This is an important element which can be used to record the current status of the model element. It has a required attribute **value**, which can have values: *stable*, *in_progress* or *known_issues*. It can also have optional subelements: **comment,** for a general comment on the current status; **issue,** to highlight a known issue with the current model which may mean care should be taken before reusing it and **contributor**, which is a **Person** element, giving details on who created the status report. This element is intended to replace the often cryptic comments left in files by those who have used them over the years, and help future users with more time or experience to solve any problems with the file.

An example of a NeuroML file containing a number of items of metadata is available here: http://neuroml.org/view.php?file=ChannelML/NaF2_Chan.xml.

This information can more easily be read in the HTML view of the file: http://neuroml.org/view.php?file=ChannelML/NaF2_Chan.xml&map=HTML.

Simple Types

A number of Simple types are defined for use at all Levels. Those not discussed already are:

**LengthUnits**	The units of the x, y, z coordinates, etc. appearing in the file, usually. Allowed values: *micrometer, millimeter, meter.* Note, micron is still permitted, but *micrometer* is preferred.
**NonNegativeDouble**	A double precision floating point value, greater than or equal to zero.
**Percentage**	A double precision floating point value between 0 and 100 inclusive.
**PositiveDouble**	A double precision floating point value, greater than zero.
**SegmentIdInCell**	Id of an individual segment in a cell (integer valued, 0 or greater).
**Units**	Unit system to use for all values for the element (and all children) for which it is an attribute. Allowed values: *SI Units*, *Physiological Units* (milliseconds, millivolts, centimeters, etc). See below for a table of physical quantities in these unit systems.
**VolumeUnits**	Units of volume. Allowed values: *cubic_millimeter, millilitre, litre.*
**YesNo**	Allowed string values: *yes* or *no.*
**ZeroToOne**	A double precision floating point value between 0 and 1 inclusive.

Table 2: Simple types in Metadata

The table below lists a number of physical quantities in the two unit systems supported by NeuroML: *SI Units*, and *Physiological Units.*

	SI Units	Physiological Units
Time	**s**	**ms**
Voltage	**V**	**mV**
Length	**m**	**cm**
Current	**A**	**µA**
Specific capacitance	**F/m^2**	**µF/cm^2**
Resistance (e.g. membrane resistance)	**ohm**	**Kohm**
Conductance	**S**	**mS**
Conductance density	**S/m^2**	**mS/cm^2**
Specific Axial Resistance	**ohm m**	**Kohm cm**
Concentration	**mol/m^3**	**mol/cm^3**

Table 3: Units of various quantities in SI and physiological units

Complex Types

The following are some complex types which have not been discussed above:

**GroupDetail**	Can be used to give a string description and a list of properties of groups which are used in multiple locations in a file.
**Manifold**	A surface defined by a set of points. Extension of **Points**.
**NonSpatialGrid**	Specifies a grid of up to 3 dimensions, without any explicit 3D location information.
**Point**	A 3D point with optional diameter.
**Point3D**	A 3D point with no diameter.
**Points**	A list of **Point** elements.
**Polygon**	A closed structure represented by a list of **Point** elements where the first point connects with the last point.
**Polyhedron**	A 3D surface which can be used to represent a cell body or other histological structure. Consists of a list of **Polygon** elements.
**PropertyDetail**	Can be used to give a description of a property which is used in multiple locations in a file.
**RectangularBox**	A Rectangular Box specified by a corner point and a width, height and depth. Can be used for positioning cell populations in 3D.
**Sphere**	A spherical structure such as a cell body or other histological feature.

Table 4: Complex types in Metadata

5. Level 1: Cell descriptions (MorphML)

A discussion on the structure of MorphML and the mapping of its elements to morphology formats used in Neurolucida, NEURON (Carnevale and Hines, 2006), GENESIS (Bower and Beeman, 1997), etc. is contained in (Crook et al., 2007).

Descriptions of cellular structures are at the heart of NeuroML. Level 1 focuses on the anatomical details of individual cells, as opposed to the electrophysiological properties or synaptic connectivity. Cells in MorphML are not only intended for use in multicompartmental neuronal modeling, and the format can be used by a range of applications which handle neuronal morphology data including those for visualization, reconstruction of cells from images, or generation of realistic cellular structures.

As mentioned, a Level 1 cell description could have as root element **morphml** or **neuroml**. Both **morphml** and **neuroml** elements have the required attribute **length_units**, which is normally set to *micrometer*, and this sets the units for the x, y, z positions and radii of the segments.

See main NeuroML paper Figure 4 for an overview of the elements needed for Levels 1-3 cells.

Element: **<cells>**

This element contains a set of **cell** elements. As is the case for channels and synapses, it is good practice to separate out individual cells into one file each, as is generally the case for well written and modularized models implemented in NEURON or GENESIS for example.

Element: **<cell>**

The required **name** attribute is used to specify a unique string identifier for this cell. An optional **status** element can be added giving details of the model's current condition. The **cell** element

can have any of the elements in the **metadata** and **referencedata** groups as subelements, and the **notes** element for giving a brief description of the cell should be considered as a minimum. The permitted subelements are: **segments**, **cables**, **cellBody**, **spines**, **freePoints**, of which only **segments** is required.

> An example of a cell in MorphML containing the **segments** and **cables** elements is available online here: http://neuroml.org/view.php?file=MorphML/CablesIncluded.xml. A mapping of this cell to a NEURON cell template is here: http://neuroml.org/view.php?file=MorphML/CablesIncluded.xml&map=NEURON and a GENESIS readcell file for creating the equivalent set of compartments is here: http://neuroml.org/view.php?file=MorphML/CablesIncluded.xml&map=GENESIS.
>
> A example of a single segment/cable cell is shown in Section 10.1.

Element: **<segments>**

The **segments** element contains the set of one or more **segment** elements.

Element: **<segment>**

The **segment** element represents the fundamental building block of complex neuronal morphologies (e.g. the piece between two points in a digital reconstruction, or a compartment in a compartmental simulator like GENESIS). Each **segment** in a cell will have a unique nonnegative integer **id**, a **parent** attribute for the segment it is electrically connected to (optional attribute, but only left out in the case of the root segment), an optional string attribute **name**, and an optional reference to the **id** of the **cable** of which it is part.
The **segment** elements can have subelements **proximal** and **distal**, representing the closest and furthest points respectively from the soma or the segment which represents the root of the tree (i.e. has no parent). Each has attributes **x**, **y**, **z**, **diameter** to specify the 3D location and the diameter of the endpoints (the segment does not have to be cylindrical). The **proximal** element is optional and its absence indicates that the proximal point of the segment should be taken from the parent **segment**. If the 3D locations of the **proximal** and **distal** points are equal, the segment can be assumed to be spherical. The surface area of cylindrical segments (e.g. for use at Level 2 when calculating total conductance from a conductance density) is calculated from the curved surface only (a spherical segment where **proximal** = **distal** would be calculated as the surface of the sphere). A morphology file specifying only segments can be valid, however the **cables** element would normally be present too if the neurites are to be grouped into soma, basal/apical dendrites, etc.

Element: **<cables>**

The **cables** element contains the set of one or more **cable** elements.

Element: **<cable>**

A **cable** represents an unbranched section of a cell morphology, e.g. a dendritic section or the soma. While cables can be used at subsequent Levels to define the electrical properties of cells, at Level 1 they can best be thought of as a convenient way to create sets of connected segments for the purposes of defining interesting groups, e.g. dendrites, axons. One or more **segment** elements will go to create a cable, so defining the 3D path of the cable. The **cable** concept is roughly equivalent to the section object in NEURON (Crook et al., 2007). As the cable is unbranched, the segments which form this cable should only have **distal** points specified (apart possibly from the first segment), and should have the expected parent child relationships for such a linked set. The unique nonnegative integer attribute **id** is required, and a string attribute **name** is optional. A **parent** attribute for the parent cable is optional, but this is superfluous, as the parent cable can be worked out from the segments (this may be removed in future versions). An optional attribute **fract_along_parent** can be specified when the **cable** is electrically connected to a point which is not at the distal point of the parent cable. More details

on this element and the mapping of segments/cables to other formats can be found in (Crook et al., 2007).

Sets of **cable** elements can be related and this can be specified by including a **group** subelement in the **cable**. Apart from allowing cables to be grouped anatomically (apical or basal dendrites, etc.) this facilitates applying conductances and biophysical properties etc. at higher Levels. neuroConstruct (http://www.neuroConstruct.org, (Gleeson et al., 2007)) uses the **group** names: *soma_group*, *dendrite_group* and *axon_group* to distinguish the main types of neuronal cables, a cable belonging to only one of these groups, but this is not part of the formal specification at present. Note: an alternative way to define groups of cables is to use the **cablegroup** element.

Element: **<cablegroup>**

While groups of cables can be defined by listing within a **cable** element the groups relevant for that cable, another option is to first list all the **cable** elements in **cables** (defining the **id** and any properties of the cable) and afterwards define a number of **cablegroup** elements, each with a required attribute **name**, and each containing a list of **cable** subelements with an attribute **id** referring back to the original **cable**. This second option for defining cables is the more natural way to define such groupings in some applications (such as NEURON). A parsing application should support both options and would normally map the grouping to its own preferred format on import of a MorphML file. This freedom of different ways to define groups of segments may be limited in future versions.

> An example showing the **cablegroup** element in a cell exported from NEURON is available here: http://neuroml.org/view.php?file=ChannelML/PyramidalCell.xml.

There is an optional subelement **inhomogeneous_param** which can be included here to define a parameterization over the cable group.

Element: **<inhomogeneous_param>**

This element can be used to define a parameter which varies over a specific cable group (e.g. the path length from the soma over the dendrites). This can be used by other elements for a quantity which varies as a function of this parameter (e.g. channel density which varies as a function of this path length, see **variable_parameter** in Section 6). The **name** attribute gives a unique reference to this parameterization, and the **variable** attribute names the variable (e.g. p) to be used in subsequent expressions involving this parameter. A required subelement is **metric**, which can contain string with values: *Path Length from root*, *3D radial position* or *3D path length from line*. Two optional elements are **proximal** (if present the value of the parameter is translated to its attribute **translationStart**), and **distal** (if present, the parameter is normalised so that it has the value **normalizationEnd** at the distal point).

> An example showing the usage of the **inhomogeneous_value** and **variable_parameter** elements in a cell with non uniform electrical properties is available here: http://neuroml.org/view.php?file=ChannelML/InhomogeneousBiophys.xml.

Element: **<cellBody>**

This is used for a detailed anatomical representation of the soma. It consists of a **Polygon**, **Polyhedron** or **Sphere**. This element is not normally used in cells which will be part of a simulation of cable properties. A representation of the soma in **segment** elements should be used to allow use of the cell in neuronal simulations.

Element: **<spines>**

Spines on dendritic segments can be specified with this element. It is based on the **segment** element with **proximal** and **distal** points, but also has optional length, volume and shape

elements. Note normally spines are not present in morphological reconstructions, and instead a spine correction factor is applied to the membrane properties. If spines are to be explicitly included in a compartmental simulation, it may be better to use a normal **segment** element and include the spines in their own group, for maximum compatibility with current simulators.

Element: **<freePoints>**

This element (which may be associated with a particular **cell** element or be a subelement of **features** under **morphml**) contains a list of elements of type **Point** which identifies interesting features. It can be present in a file which has been created from a morphological reconstruction, but the data would not normally be used directly in neuronal simulations involving cells. These points could form the basis of the end points in **segment** elements in a subsequent cleaned up version of the reconstruction.

Element: **<features>**

This can be a subelement of a **morphml** element and can contain a number of items which have been recorded in a neuronal reconstruction, but not be associated with a specific cell. The subelements here can include **path** and **freePoints**, both of which can be used for sets of connected points (**path** elements can be linked), **manifold**, **polygon**, **polyhedron** and **sphere** for interesting geometrical features. These elements would not normally be used directly in neuronal simulations, but could form the basis, e.g. of the end points in **segment** elements in a subsequently cleaned up version of the reconstruction.

6. Level 2: Cell descriptions (MorphML & Biophysics)

A key extension of the Level 1 description of cells is the specification of the passive and active electrophysiological properties of the cell, which can be used in simulations of the cell's electrical behavior. Addition of the element **biophysics** makes the cell Level 2 compliant (see Figure 4 in the main NeuroML paper). Note: an application which is only interested in the anatomical aspects of a cell (e.g. a visualization package) could potentially load Level 2 files also, and just ignore the **biophysics** element.

The addition of details of the passive electrical properties of the cell and locations of voltage dependent conductances enable various strategies for creating simulations of the electrical activity of the cell. The cable modeling approach of NEURON and the compartmental modeling of GENESIS/MOOSE are just two ways the data in a NeuroML Level 2 cell could be used in a simulation. While the current version of NeuroML is heavily influenced by these simulators, other simulators can use the data contained in these elements in a manner particular to them, e.g. PSICS calculates the locations of individual ion channels on the cell membrane, divides the cell up into a number of compartments (based on a maximum structural discretization, not constrained by cables/branch points) and simulates these.

> A good introduction to the cable theory of dendrites is contained in chapter 2 of (Koch and Segev, 1998), and chapter 3 gives a background on how such structures might be represented by compartmental models. Chapter 5 of the GENESIS book (Bower and Beeman, 1997) also gives an introduction to cable modeling and how these can be represented as compartments in that simulator (the chapters of that book are available at http://www.genesis-sim.org/GENESIS/iBoG/iBoGpdf).
>
> NEURON's use of the section object for cable modeling is explained in chapter 5 of the NEURON Book (Carnevale and Hines, 2006).
>
> The PSICS simulator's handling of detailed neuronal morphologies is explained here: http://www.psics.org/formats/morphology.html and its handling of cellular electrical properties is outlined here: http://www.psics.org/formats/properties.html.

Element: <biophysics>

This element contains subelements describing the passive and active electrical properties of the cell. It has required attribute **units** (with value *SI Units* or *Physiological Units*), which will set the unit system for all physiological parameters of the subelements, e.g. maximum conductance density of channels. The element contains at least one **mechanism** subelement, one each of **spec_capacitance**, **spec_axial_resistance**, and zero or one each of **init_memb_potential** and **ion_props.**

Element: <mechanism>

The term mechanism refers to a dynamical property of the cell membrane whose behavior depends on the state of the membrane (e.g. voltage) and in turn can influence properties of the membrane (e.g. through an ionic current flow). Types of mechanism currently supported are voltage (and/or ligand) gated conductances on the cell membranes or (intercellular) ionic concentrations (e.g. a pool of calcium just below the membrane surface which decays to a steady concentration over time). The attribute **name** is required, and should refer to the name of a channel (specified in a **channel_type** element) or ionic concentration (**ion_concentration** element) in a ChannelML file (or potentially in the same NeuroML Level 2+ file) where the behavior of the mechanism is described (e.g. the voltage dependent changes to the conductance of the channel). The **type** attribute has a value *Channel Mechanism* or *Ion Concentration*. Note that there is no separate element for specifying a specific membrane resistance/leak conductance (as there is for specific capacitance and specific axial resistance); a **mechanism** element would be used for this conductance also. The optional **passive_conductance** attribute takes a Boolean value and if *true* indicates that this represents a passive (leak, non selective) conductance and is completely described by its conductance density and reversal potential (i.e. doesn't require a separate ChannelML description). Note that the surface area of cylindrical segments on which these mechanisms are placed is calculated from the curved surface only (a spherical segment where **proximal** = **distal** would be calculated as the surface of the sphere).

The **mechanism** element can have one or more subelements of each of **parameter** or **variable_parameter**.

> Section 10.1 shows an example of a cell with multiple **mechanism** elements (along with **spec_capacitance** and **spec_axial_resistance** elements) and gives an outline of how the information in these elements can be used to calculate the time varying membrane potential of a cell model. A further example of a cell using these elements is available http://neuroml.org/view.php?file=ChannelML/MossyCellBiophys.xml.

Element: <parameter>

This describes the value of a parameter related to a **mechanism** which is of fixed value across a group of cables. This element has **name** and **value** attributes. A number of names of parameters are considered particularly relevant: *gmax* for the maximal conductance density of a channel mechanism; and *erev* (or just *e*), the reversal potential of a channel, although a channel can have any named parameter associated with it. A parameter will have one or more **group** subelements, and the string contents of these refer to **cable** groups already defined in the cell. In this way, a file can specify that a **mechanism** (e.g. with **name** FastSodiumConductance and **type** *Channel Mechanism*) can have a parameter with **name** *gmax* and a certain **value** and specify that it is present on **group** *soma_group*.

Element: <variable_parameter>

This describes the value of a parameter related to a **mechanism** which varies over the part of the cell it is present on. It references one of the parameterizations defined in an **inhomogeneous_param** subelement of **cablegroup**. There is a required **name** attribute, which corresponds to the **name** attribute in **parameter** (could be *gmax* or *erev*). There will be a single **group** subelement, together with a **inhomogeneous_value** subelement, which contains two

required attributes: **param_name** (references the **name** used in the **inhomogeneous_param** element in the **cablegroup** element) and value (contains an equation showing how parameter changes as function of **variable** attribute in **inhomogeneous_param** element).

> An example showing the usage of the **inhomogeneous_value** and **variable_parameter** elements in a cell with nonuniform electrical properties is available http://neuroml.org/view.php?file=ChannelML/InhomogeneousBiophys.xml.

Element: **<spec_capacitance>**

This describes the value of the specific capacitance (capacitance per unit area) over the cell membrane. As the specific capacitance may not be uniform over the cell (or may be set non-uniform for the purposes of simulating spine density), different regions of the cell can have different values for this. The values are set with either a **parameter** subelement (of type **UnamedParameter**, similar to the **parameter** for a mechanism, but without the **name** attribute), or a **variable_parameter** subelement. See Table 3 for details of units.

Element: **<spec_axial_resistance>**

This describes the value of the specific axial resistance (or cytoplasmic resistivity) along the segments of the cell. As this may not be uniform over the cell, different regions of the cell can have different values. The values are set with either a **parameter** subelement (of type **UnamedParameter**, similar to the **parameter** for a mechanism, but without the **name** attribute), or a **variable_parameter** subelement. See Table 3 for details of units.

Element: **<init_memb_potential>**

This optional element is used to associate a default initial membrane potential with the cell. While this is not an inherent electrophysiological property of the cell (the cell, unless spontaneously firing, will have its own resting potential due to the conductances present and will most likely differ from this value), this value can be useful when storing cell models and porting them between simulators. The values are set with either a **parameter** subelement (similar to the **parameter** for a mechanism, but without the **name** attribute), or a **variable_parameter** subelement.

Element: **<ion_props>**

This optional element is used to give information on the ions which will influence the behavior of the cell. It can be used to give (initial) internal (parameter name *conc_i*) and extracellular concentrations (*conc_e*) of the ions, or to provide information on fixed ionic reversal potentials (*e*). The values are set with either a **parameter** or a **variable_parameter** subelement.

7. Level 2: Channel & synaptic mechanisms (ChannelML)

Descriptions of channel and synaptic mechanisms can be present together with cells in a Level 2 NeuroML file (with **neuroml** as root element). However, for greater modularity, and ease of use with tools for analyzing and simulating the channels, one mechanism (e.g. channel or synaptic mechanism) per file (with root element **channelml**) is recommended.

Note that ChannelML descriptions of synaptic mechanisms would normally be used as part of a Level 3 network description (as opposed to being used in Level 2 single cell models).

The **channelml** element (and the **channels** element under a **neuroml** root) has required attribute **units** (with value *SI Units* or *Physiological Units*), which will set the unit system for all physiological parameters of the mechanism. The permitted subelements for the (**channelml** or **channels**) element are: **channel_type**, **synapse_type** and **ion_concentration**.

See Figure 5 in the main NeuroML paper for an overview of the elements used in ChannelML.

Element: **<channel_type>**

This represents a membrane conductance distributed across a region of a cell which is voltage and/or an ligand gated (as opposed to channels concentrated at a synapse and triggered by a presynaptic neurotransmitter release). This element has a required attribute **name**, which will be used when placing the channel mechanism on cells. The **status** element, **metadata** and **referencedata** groups of elements and the **parameters** element are permitted subelements. The required **current_voltage_relation** element will contain the details of the behavior of the channel with varying membrane potential.

> An example of the Na^+ channel from the Hodgkin Huxley model (Hodgkin and Huxley, 1952) expressed in using the **channel_type, current_voltage_relation, gate** & **transition** elements is available in Section 10.2 and also online here: http://neuroml.org/view.php?file=ChannelML/NaChannel_HH.xml.
>
> The equivalent code for the channel in the NMODL language (http://www.neuron.yale.edu/neuron/static/papers/nc2000/nmodl.htm) of NEURON is here: http://neuroml.org/view.php?file=ChannelML/NaChannel_HH.xml&map=NEURON, for the GENESIS script using the tabchannel object (http://www.genesis-sim.org/GENESIS/Hyperdoc/Manual-26.html#ss26.62) is here: http://neuroml.org/view.php?file=ChannelML/NaChannel_HH.xml&map=GENESIS. The expression of this channel in PSICS format (http://www.psics.org/formats/channels.html) is here: http://neuroml.org/view.php?file=ChannelML/NaChannel_HH.xml&map=PSICS.

Element: **<parameters>**

This contains a list of **parameter** subelements, each of which has **name** and **value** attributes. These named parameters can be used in generic expressions (e.g. for gating variables), and implementations of the channel ideally should allow these parameters to be subsequently changed (i.e. be publicly accessible fields). Note however that pre-calculated tables of rate information may have to be updated if these parameters are changed.

> A ChannelML example containing **parameter** elements is available here: http://neuroml.org/view.php?file=ChannelML/NaF2_Chan.xml. The equivalent code in the NMODL language of the NEURON simulator is here: http://neuroml.org/view.php?file=ChannelML/NaF2_Chan.xml&map=NEURON and the channel in GENESIS script is here: http://neuroml.org/view.php?file=ChannelML/NaF2_Chan.xml&map=GENESIS.

Element: **<current_voltage_relation>**

This element holds the information on how the current through the channel changes depending on the membrane potential (and possibly on the concentration of some ion). The attribute **cond_law** describes the relationship between the voltage and the current. The currently permitted values for this are *ohmic* and *integrate_and_fire* (note more complex ionic current descriptions based on the Goldman-Hodgkin-Katz equation (Hille, 2001) are not currently supported). The reason **cond_law** is an optional element is for backwards compatibility with an earlier version of ChannelML (when the **ohmic** and **conductance** elements were used), but it is most likely that **cond_law** will be a required attribute in future versions. If its value is *integrate_and_fire*, the **integrate_and_fire** element below, and no other attributes should be used. If its value is *ohmic*, the **ion**, **default_gmax** and **default_erev** attributes at least should be used (see Table 3 for details on units). These represent, respectively, the ion which flows through the channel (which for a leak current should be set to *non_specific*), the default value for the maximum conductance density of the channels, and the default value for the reversal

potential of the ion. Note that these last two attributes are not actually properties of the channel mechanism, but are completely dependent on the density of the channels on the cell and the intracellular and extracellular concentrations of the ion in question. However, almost all implementations of the channel will use these concepts, and so the option to specify default values is given. These values will normally be overwritten when the channel is placed on a cell.

The **charge** attribute is used if the ion has a valence greater than one. The **fixed_erev** attribute is used in the case where the ion in question flows through the channel (and so influences the internal concentration of the ion) but experiences a fixed reversal potential (e.g. Ca^{2+} channels in (Traub et al., 2005) and the granule cell model of (Maex and De Schutter, 1998). For *ohmic* channels, the main subelement is the **gate** element for describing the main gating complex (note if this is absent it implies that it is a passive conductance), but preceding that, the optional **conc_dependence**, **conc_factor**, **q10_settings** and **offset** elements are allowed.

Element: **<integrate_and_fire>**

This element describes a membrane mechanism which will cause the cell to behave like an Integrate and Fire neuron. There are many ways to describe Integrate and Fire mechanisms; this one is based on the implementation in NEURON of the COBA IandF cell as described in (Brette et al., 2007). The mechanism is specified by the attributes **threshold**, **t_refrac**, **v_reset** and **g_refrac**. This mechanism will normally be placed on a cell which also has a passive/leak conductance specified by a separate ChannelML mechanism file, whose reversal potential will set the cell's resting potential. The cell will depolarize on current injection or excitatory synaptic input, with current leaking out through a passive conductance. If the cell passes the **threshold**, the mechanism will reset the cell's potential to **v_reset** (a spiking event will be sent to any postsynaptic synapse at this point). For a period given by **t_refrac**, the mechanism will provide a current via a conductance of **g_refrac**, with reversal potential **v_reset**. Note that if **g_refrac** is high, this will effectively clamp the cell at **v_reset** for a time **t_refrac**. After this time the cell will integrate all inputs as normal with a time constant given by the passive properties of the cell.

> An example of a ChannelML file using the **integrate_and_fire** element is here: http://neuroml.org/view.php?file=ChannelML/IntFireMechanism.xml and the equivalent representation in a NEURON mod file is here: http://neuroml.org/view.php?file=ChannelML/IntFireMechanism.xml&map=NEURON.
>
> A neuroConstruct project which includes a cell using the **integrate_and_fire** mechanism which can be mapped to simulators supporting PyNN (Davison et al., 2008) can be found here: http://www.neuroconstruct.org/samples/index.html#Ex8_PyNNDemo.

Element: **<conc_dependence>**

This element is used to describe a parameter for intracellular concentration which can be used in the gating expressions for channels whose rates depend on a particular ion's concentration. An example would be the dependence of some potassium channels on calcium concentration as well as membrane potential, e.g. the forward rate for the activation variable is given by 2500/(1 + ((0.0015 *(exp (-85*v))) / ca_conc)). The **name** attribute in this element gives a unique reference for this concentration parameter, the **ion** attribute names the ion whose concentration is varying, and an optional **charge** attribute is used if the ion has a valence greater than one. The **variable_name** attribute (e.g. *ca_conc*) specifies the name of the variable which will be used in expressions. The **min_conc** and **max_conc** attributes are very useful for the implementation of the channel mechanism, as 2D tables are often generated before simulation for the rates at various voltages and concentrations.

An example of a file using the **conc_dependence** element is here: http://neuroml.org/view.php?file=ChannelML/KCa_Channel.xml and the equivalent representation in a NEURON mod file is here: http://neuroml.org/view.php?file=ChannelML/KCa_Channel.xml&map=NEURON, and as a GENESIS script using the tab2Dchannel object is here: http://neuroml.org/view.php?file=ChannelML/KCa_Channel.xml&map=GENESIS.

Element: **<conc_factor>**

This element is used to describe a concentration dependent multiplicative factor for a channel's conductance which is independent of the membrane potential. The **ion** attribute names the ion whose concentration is varying, and an optional **charge** attribute is used if the ion has a valence greater than one. The **variable_name** attribute (e.g. *ca_conc*) specifies the name of the variable which will be used in the expression contained in **expr**. This expression should be evaluated at each time step and used to scale the conductance of the channels. The **min_conc** and **max_conc** attributes are very useful for the implementation of the channel mechanism, as 2D tables are often generated before simulation for the rates at various voltages and concentrations.

An example of a file using the **conc_factor** element is here: http://neuroml.org/view.php?file=ChannelML/Kc_fast_Chan.xml and the equivalent representation in a NEURON mod file is here: http://neuroml.org/view.php?file=ChannelML/Kc_fast_Chan.xml&map=NEURON, and as a GENESIS script using the tabchannel object is here: http://neuroml.org/view.php?file=ChannelML/Kc_fast_Chan.xml&map=GENESIS.

Element: **<q10_settings>**

Q_{10} scaling affects the rates of the activation and inactivation variables. It allows rate equations determined at one temperature to be used at a different temperature. If tauExp is the experimentally measured tau, the rate at temperature T is given by tau(T) = tauExp / **q10_factor** ^ ((T - **experimental_temp**)/10). NOTE: if **fixed_q10** is specified the expression will be tau(T) = tauExp / **fixed_q10**, and the **experimental_temp** can be used to check that a simulation is being run at the desired temperature. The optional **gate** attribute can be used to specify which of the gates this rate adjustment refers to. If it is absent, the scaling applies to all gates present.

Element: **<offset>**

Experimental data for the activation and inactivation rates for channels are often not available in the cell type/species being modeled, and modelers reuse channels of a similar type from a different system. One adjustment which is often made in such reused channels is to introduce a fixed change in the voltage dependence of the rate expressions (e.g. the Golgi cell channels in (Maex and De Schutter, 1998) reuse the granule cell channels with a voltage shift of -10 which causes the Golgi cells to fire spontaneously). Such a voltage offset can be added in a ChannelML file with the **offset** element, the **value** attribute containing the offset amount.

An example of a file using the **q10_setting** and **offset** elements is here: http://neuroml.org/view.php?file=ChannelML/NaF_Chan.xml and the equivalent representation in a NEURON mod file is here: http://neuroml.org/view.php?file=ChannelML/NaF_Chan.xml&map=NEURON, and as a GENESIS representation of the channel is here: http://neuroml.org/view.php?file=ChannelML/NaF_Chan.xml&map=GENESIS.

Element: **<gate>**

This element describes a gating complex for the channel. It specifies a number of states in which the gating complex can be found, along with expressions for the rates of transitions between these. There is a required **name** attribute which gives a unique reference for the gate, e.g. m, h or n. There can be a single gating complex (with potentially many open and closed states) for a channel, or a set of gating complexes (e.g. activating or inactivating). If there are multiple copies of the same gating complex present, this can be expressed by setting the optional **instances** attribute to greater than one (e.g. 3 instances of the activating component of a Na^+ channel). For individual channels, all gating complexes must be in an open state for the channel to conduct ions. However, most implementations of these types of channels represent the mean behavior of many channels as a conductance per unit area of the cell membrane. In this case, the expressions for the rates of transitions between open and closed states are used to determine the values of a number of gating variables, and the overall conductance density is given by the product of these quantities (e.g. $G_{Na} = G_{max} * m^3 * h$).

This element will have at least one **open_state** and one **closed_state** subelements. For each transition between these, up to two **transition** elements and one **time_course** and **steady_state** element will be present.

Element: **<closed_state>**

This element describes a state in which the gating complex does not contribute to the channel conductance, i.e. a non-permissive state. The attribute **id** is used to reference this state in the **transition**, **time_course** and **steady_state** elements.

Element: **<open_state>**

This element describes a state in which the gating complex does contribute to the channel conductance, i.e. is in a permissive state. The attribute **id** is used to reference this state in the **transition** elements, etc. There is an optional **fraction** attribute which can be used to give a fractional contribution of the gate in this state to the overall conductance of the channel.

Element: **<transition>**

This element describes a single transition between two defined states. In a channel defined according to the Hodgkin Huxley formalism, there will normally only be one open and one closed state in a gating complex (with possibly **instances** >1 for the **gate** describing the activating component), and forward (closed -> open) and backward (open -> closed) transition rates described. This element has a required **name** attribute (e.g. *alpha/beta* for forward/ backward transition rates), **from** and **to** attributes specifying the pre and post states related to the transition, and an **expr_form** attribute giving details on the form of the expression for the rates. There are 3 "built in" options[1] for the expression form: *exponential*, *sigmoid* and *exp_linear*, and if one of these is used the following attributes should be included: **rate**, **scale** and **midpoint**. The full expression for each of these forms as a function of the attribute values and the membrane potential v is given in Table 5.

The other option for the **expr_form** attribute is *generic*, indicating that a freeform expression for the rates will be provided in the **expr** attribute in terms of the membrane potential v, and possibly any concentration parameters defined in a **conc_dependence** element. This option to allow a freeform expression (e.g. 2500/(1 + ((0.0015 *(exp (-85*v))) / ca_conc))) <u>is a compromise</u> which sacrifices the possibility to force an unambiguous declarative definition of the expression (e.g. in MathML) to allow an easily hand editable (and readable) expression to be used. The limiting condition on the expression is that supporting simulators should be able to parse the mapped expression in their native scripts. Note that the mapping to GENESIS replaces the round brackets with curly brackets which are required when evaluating some functions, and that spaces after some functions are necessary, so exp (v) (mapped to exp {v}) is preferred to

[1] Note these forms of rate expressions are supported for historical reasons; they are commonly used in published models but no inference should be made as to their biophysical plausibility.

exp(v). Expressions in this format have been successfully tested on NEURON, GENESIS and PSICS. See http://www.neuroml.org/NeuroMLValidator/Samples for examples of such files.

exponential	$\text{rate} \times e^{\frac{v - \text{midpoint}}{\text{scale}}}$
sigmoid	$\dfrac{\text{rate}}{1 + e^{\frac{v - \text{midpoint}}{\text{scale}}}}$
exp_linear	$\dfrac{\text{rate} \times \dfrac{v - \text{midpoint}}{\text{scale}}}{1 - e^{-1 \times \frac{v - \text{midpoint}}{\text{scale}}}}$

Table 5: In built expression types for **transition** element

Elements: **<time_course>, <steady_state>**

An alternative method for describing the transitions between open and closed states is to describe the time course for coming to a steady state of transition at a given voltage. The **time_course** element coupled with the **steady_state** element provide a description of the gating variables which is attractive to experimentalists, as these quantities are more experimentally accessible. The normal relations between the forward (alpha) and backward (beta) rates and the time course (tau) and steady state (inf) descriptions are:

$$\text{tau} = \frac{1}{\text{alpha} + \text{beta}} \qquad \text{inf} = \frac{\text{alpha}}{\text{alpha} + \text{beta}}$$

and these are the assumed expressions for the time course and steady states if **time_course** or **steady_state** are not present. Note that in some channels, forward and backward transitions can be present, but also one of **time_course** or **steady_state**, potentially expressing their values in terms of alpha and beta. Both of these elements have a similar formats to **transition**, with a required **name** attribute, **from** and **to** attributes specifying the direction of the transition, and an **expr_form** which can have value *exponential*, *sigmoid*, *exp_linear*, or *generic*.

> An example of a file using the **time_course** and **steady_state** elements (and expressing the rates using **expr_form** = *generic*) is here: http://neuroml.org/view.php?file=ChannelML/KA_Channel.xml and the equivalent representation in a NEURON mod file is here: http://neuroml.org/view.php?file=ChannelML/KA_Channel.xml&map=NEURON, and as a GENESIS representation of the channel is here: http://neuroml.org/view.php?file=ChannelML/KA_Channel.xml&map=GENESIS.

Element: **<synapse_type>**

This element is used for describing (mainly phenomenological as opposed to biophysical) models of synaptic conductance changes. As such they are point processes (as opposed to mechanisms distributed over the membrane) which specify the time varying conductance through a synapse when a presynaptic event happens (e.g. neurotransmitter release due to a presynaptic connection point reaching threshold). The one exception to the above is when the subelement is **electrical_syn**, for a conductance at an electrical synapses between two cells.

This element has a required attribute **name**, giving a unique reference for this synapse type, and which is used in NetworkML files when describing synaptic connections between cell populations. The **status** element, **metadata** and **referencedata** groups of elements are optional

subelements to give more information on the background of the model. One of the following subelements is required, which specifies the type of synapse: **doub_exp_syn**, **blocking_syn**, **multi_decay_syn**, **fac_dep_syn**, **stdp_syn, electrical_syn**.

Chemical synapses (i.e. all elements except **electrical_syn**) can be used in two ways: at a **connection** instance of a **projection** between pre and postsynaptic **population** instances, and at a **site** instance of an **input** into a cell which is of type **random_stim** (see Section 9 for more details). In the former case the postsynaptic conductance change event occurs when the presynaptic site passes the **threshold** (as specified in the **synapse_type** element, with a possible **internal_delay**, etc.). In the latter case a **frequency** is given for the triggering of the synaptic event. An electrical synapses can be specified in a **projection**, and this results in a two continuous current which have opposite signs at the pre & post synaptic sites.

> An introduction to the modelling of synaptic conductances can be found in chapter 6 of the GENESIS book (Bower and Beeman, 1997).
>
> Chapter 10 of the NEURON Book (Carnevale and Hines, 2006) also discusses modeling of synaptic transmission.
>
> Section 10.4 gives an example of a network in NeuroML which contains both external synaptic input and transmission of a synaptic event between two cells.

Element: **<doub_exp_syn>**

A basic synaptic mechanism describing a conductance change with exponentially rising and decaying time courses. The **max_conductance** attribute gives the peak conductance which is reached, **rise_time** is used for the time course of the rising exponential, **decay_time** gives the time course for the decaying exponential, and the **reversal_potential** attribute is used to define the zero current potential, which is used to calculate the driving force for the synaptic conductance. A single exponential synapse can be modeled by setting **rise_time** to zero, and an alpha synapse can be created if **rise_time** = **decay_time**.

> An example showing the usage of the **doub_exp_syn** element in a synapse model is presented in Section 10.3 along with the equations for the current through and conductance of the synapse.
>
> This example is also available online here: http://neuroml.org/view.php?file=ChannelMLSyns/DoubExpSyn.xml. The implementation of the synapse in the NMODL language of the NEURON simulator (based on the Exp2Syn inbuilt example) is here: http://neuroml.org/view.php?file=ChannelMLSyns/DoubExpSyn.xml&map=NEURON, and an implementation in GENESIS script using the synchan object is here: http://neuroml.org/view.php?file=ChannelMLSyns/DoubExpSyn.xml&map=GENESIS.

Element: **<blocking_syn>**

This synaptic mechanism scales the synaptic conductance in a voltage-dependent manner and inherits all of the attributes of **doub_exp_syn**, and is defined in a **block** subelement.

Element: **<block>**

This allows specification of the block of NMDA receptors by Mg^{2+} and is based on the mechanism outlined in (Gabbiani et al., 1994) and (Maex and De Schutter, 1998). See also (Jahr and Stevens, 1990). This element requires attribute **species** for the substance blocking the synapse, **conc** for the (currently fixed) external concentration of the species, and **eta** and **gamma** for the voltage and concentration dependence expression. The conductance of the synapse which would be determined by the attributes from **doub_exp_syn** will be scaled by a factor of 1/(1 + **eta** * [**conc**] * exp(-1 * **gamma** * V)) at each time step.

An example showing the usage of the **blocking_syn** and **block** elements in an NMDA synapse model is available here:

http://neuroml.org/view.php?file=ChannelMLSyns/NMDA.xml. The implementation of this synaptic mechanism in the NMODL language of the NEURON simulator is here: http://neuroml.org/view.php?file=ChannelMLSyns/NMDA.xml&map=NEURON, and an implementation in the GENESIS script using the Mg_block object is here: http://neuroml.org/view.php?file=ChannelMLSyns/NMDA.xml&map=GENESIS.

Note that due to GENESIS not currently supporting the more complex synapse types (e.g. **multi_decay_syn** or **fac_dep_syn**), **blocking_syn** was limited to being an extension of **doub_exp_syn** for wider simulator support of this element.

Element: **<multi_decay_syn>**

This also extends the basic **doub_exp_syn** to allow specification of up to two extra decay components. This can be used for modeling synapses which have one or more slow decay components to their EPSCs. The additional attributes **max_conductance_2**, **decay_time_2** and **max_conductance_3**, **decay_time_3** are used to define these. It is assumed that only the two attributes for the second decay component, or all four of these attributes will be present.

Element: **<fac_dep_syn>**

This element allows creation of a short term plasticity synaptic model based on the popular model from Tsodyks and Markram (Tsodyks et al., 2000; Tsodyks and Markram, 1997). In this model synapses can be depressing, facilitating or have both depressing and facilitating components. The model is defined by a recursion relation for the remaining synaptic resources after the arrival of n+1 spikes, given the previous arrival of n spikes. When the first spike arrives at the synapse a fraction of the synaptic resource (U_1) is used, such that the fraction remaining after the 1st spike is R_1=1-U_1. Set U_1 with attribute **init_release_prob**. After the (n+1)th spike the fraction of remaining resource is,

$$R_{(n+1)} = R_n(1-U_n)\exp(-\frac{\Delta t}{\tau_R}) + 1 - \exp(-\frac{\Delta t}{\tau_R})$$

where τ_R is the recovery time-constant set with attribute **tau_rec**. In between spikes (for the duration Δt) there is replenishment of synaptic resources with linear dynamics having time-constant τ_R**.** Facilitation is modeled by increasing the fraction of resources employed by the next spike $U_{(n+1)}$ such that upon the arrival of the (n+1)th spike,

$$U_{(n+1)} = U_n \exp(-\frac{\Delta t}{\tau_F}) + U_1(1 - U_n \exp(-\frac{\Delta t}{\tau_F}))$$

where τ_F is the facilitation time-constant set with attribute **tau_fac**. In between spikes there is a decay in the accumulated increase of synaptic resources utilised by each spike. Again, this decay is linear and has a single time-constant τ_F. The magnitude of the change in synaptic conductance due to the nth spike will be scaled by a factor R_nU_n, i.e. a maximal change in the event that all resources are available R_n=1 and all resources are utilised U_n=1. In the limit $\tau_F \rightarrow 0$ the synapse is depressing only, since no facilitation can accumulate. In the limit $\tau_R \rightarrow 0$ the synapse is facilitating only, since all resources are instantly replenished.

An example showing the usage of the **fac_dep_syn** element in a plastic synapse model is available here: http://neuroml.org/view.php?file=ChannelMLSyns/STPSynapse.xml. The implementation of the synapse in the NMODL language of NEURON is here: http://neuroml.org/view.php?file=ChannelMLSyns/STPSynapse.xml&map=NEURON.

Element: <stdp_syn>

This element allows implementation of the Spike Timing Dependent Plasticity (STDP) model of Song, Miller and Abbott (Song et al., 2000) with all to all spike pairing. In this model synaptic conductance is potentiated by a factor Δw_+ or depressed by a factor Δw_- based upon the relative timing between each pair of pre and post-synaptic spikes. The model is defined by the learning rule,

$$\Delta w_+(\Delta t) = A_+ \exp\left(-\frac{\Delta t}{\tau_+}\right)$$

for spikes with positive timing difference $\Delta t = t_{post} - t_{pre}$, (i.e. pre before post) and

$$\Delta w_-(\Delta t) = -A_- \exp\left(\frac{\Delta t}{\tau_-}\right)$$

for spikes with negative timing difference (i.e. post before pre). The resulting conductance scaling w(t) at time t is the sum of all of these changes for all previous spike pairs. The parameters $A_{+/-}$ control the maximum size of synaptic update as a fraction of the maximum synaptic conductance (g_{max}) for potentiation and depression respectively and are set with attributes **del_weight_ltp** and **del_weight_ltd**. The time constants $\tau_{+/-}$ determine the time lag for which positive and negative timing differences respectively lead to modification of the synaptic conductance factor and can be set with attributes **tau_ltp** and **tau_ltd**.

This STDP model truncates changes to the synaptic conductance factor that would lead to factors outside of the inclusive range between zero and an upper bound w_{max} (fraction of g_{max}) such that $w(t) \in [0, w_{max}]$ for all t. Set w_{max} with the attribute **max_syn_weight**.

An example showing the usage of the **stdp_syn** element in a plastic synapse model is available here: http://neuroml.org/view.php?file=ChannelMLSyns/STDPSynapse.xml. The implementation of the synapse in the NMODL language of the NEURON simulator is http://neuroml.org/view.php?file=ChannelMLSyns/STDPSynapse.xml&map=NEURON.

Element: <electrical_syn>

Describes electrical synaptic coupling as mediated by gap junctions, which is implemented with a simple model with just a single attribute **conductance**. The current flowing across this electrical synapse is the product of this conductance (g) and the difference in membrane potential between the segments where the connection is made, i.e.

$$I_{Gap} = g \cdot (V_{post} - V_{pre})$$

An example showing the usage of the **electrical_syn** element in a gap junction model is available here: http://neuroml.org/view.php?file=ChannelMLSyns/ElectSyn.xml. The implementation of the synapse in the NMODL language of the NEURON simulator is here: http://neuroml.org/view.php?file=ChannelMLSyns/ElectSyn.xml&map=NEURON and in GENESIS script is here: http://neuroml.org/view.php?file=ChannelMLSyns/ElectSyn.xml&map=GENESIS.

Element: <ion_concentration>

This is used to define the behavior of the concentration of an ion of a particular type, which varies over time. It has been used so far to model internal calcium, which has a resting concentration due to calcium pumps in the membrane. This internal concentration can be altered by active calcium conductances, and can in turn influence the behavior of other channels (e.g.

[Ca^{2+}] dependent K^+ channels, BK, SK, etc.). This element has a required attribute **name**, which will be used when placing the mechanism on cells. The **status** element and **metadata** group of elements are permitted subelements. The **ion_species** and **decaying_pool_model** elements are the only other permitted subelements at present.

> An example showing the usage of the **ion_concentration**, **decaying_pool_model** & **pool_volume_info** elements is available here: http://neuroml.org/view.php?file=ChannelML/CaPool.xml. The implementation in the NMODL language of the NEURON simulator is here: http://neuroml.org/view.php?file=ChannelML/CaPool.xml&map=NEURON, and the GENESIS script using the Ca_concen object is here: http://neuroml.org/view.php?file=ChannelML/CaPool.xml&map=GENESIS.

Element: **<ion_species>**

This has an attribute **name**, which identifies the ion whose concentration is being altered.

Element: **<decaying_pool_model>**

This signifies that the concentration decays towards a fixed value of **resting_conc** (required attribute) with a time course given by **decay_constant** (or **inv_decay_constant** when the constant is expressed in units 1/time). An optional attribute **ceiling** can be given for the maximal concentration which the ion can have. The amount of the modeled segment/compartment to fill with the ion needs to be specified. This is specified in either the **pool_volume_info** or **fixed_pool_info** element.

Element: **<pool_volume_info>**

This represents a thin shell just inside the membrane where the concentration of the ion builds up due to a current density on the segment surface. It has a required attribute **shell_thickness** which can be used to calculate the total volume in which the concentration is changing and hence the total amount of the substance and the change in concentration due to a given current density of ion flow.

Element: **<fixed_pool_info>**

In this case the multiplicative factor which determines how quickly the internal pool 'fills' is given as a fixed value (contained in string value of element **phi**), i.e. change in concentration = phi * ion current density. Note this is a far from ideal way to express this value (as it does not take into account the surface area of the compartment), but needed to be included as this was the parameter which was used in a number of models including the NEURON implementation of the cells in (Traub et al., 2005).

8. Level 3: Cell descriptions (MorphML & Biophysics & Connectivity)

Cells which have their morphologies (Level 1) and channel distribution properties (Level 2) defined can be extended with information on their potential synaptic connectivity to create Level 3 cells. This information is contained in the **connectivity** subelement of **cell** (see Figure 4 in the main NeuroML paper).

Element: **<connectivity>**

This currently can contain one or more of **potential_syn_loc** subelements which give the allowed locations of synapse types.

Element: **<potential_syn_loc>**

This element outlines the locations on a cell where synapses of a certain type are allowed (e.g. synapses of type *Interneuron_to_Pyramidal* are allowed post synaptically on the *soma_group*). As such it does not give explicit lists of synaptic locations (such information would be contained in a NetworkML file), but provides information about potential synaptic connectivity about the cell, e.g. to applications such as neuroConstruct (Gleeson et al., 2007) which may require this information to generate networks.

The **potential_syn_loc** element has a required attribute **synapse_type** which should reference the name of a synapse in a ChannelML file, and the optional attribute **synapse_direction** which can have value *pre* (indicated cell regions allow the synapse presynaptically), *post* (synapse is allowed postsynaptically) or *preAndOrPost* (when there are connections within a cell population, gives the locations where pre or post connections can be made). One or more subelements **group** are required referencing cable groups of the cell where the synapses are allowed.

9. Level 3: Cell positions & connectivity (NetworkML)

Level 3 is used for describing networks of cells (normally positioned in 3D space), connected with synapses, together with external electrical input to drive the network. Two different types of definition are supported: instance based and template based (see Figure 6 in the main NeuroML paper for an overview of the elements used in NetworkML).

In the instance based representation, all cell positions are explicitly listed, all synaptic connections are defined down to the cell and segment id of pre and post connection site (together with synaptic conductance scaling weights, synaptic delays, etc.) and the exact locations of inputs are defined.

In the template based representation, a “recipe” for creating the cell positions, connections and inputs is given. This latter representation is clearly much more compact, but the range of possible options for creating the connections etc. is vast, and the current specifications only cover a fraction of the possibilities. Also, different implementations will have different algorithms for creating the networks, e.g. a set of 100 cells randomly distributed in a rectangular region, and so it will not normally be possible to get an identical network on multiple simulators from this representation (without specifying the random seed and using the same algorithm for generation). Work is underway to extend the range of network descriptions supported in this part of the language through closer compatibility with the PyNN initiative (Davison et al., 2008), which is developing a Python based API for simulator independent procedural network descriptions (i.e. having common concepts of Populations, Projections, Connectors, etc.)

The instance based description provides an unambiguous description of the network which can be exchanged between applications. Use of compression technologies and binary formats such as HDF5 means that quite large networks can be stored in this format in reasonably sized files. There is currently no formal way to validate NetworkML HDF5 files (there is no equivalent of XML Schema documents defining the structure) but neuroConstruct (http://www.neuroconstruct.org/docs/Glossary_gen.html#HDF5) can import & export instance based NetworkML files in both XML and HDF5 formats.

The NetworkML elements described here can be present in a Level 3 NeuroML file (with root element **neuroml**) or in a standalone NetworkML file with root element **networkml**. Both of these have a required attribute **length_units**, which is normally set to *micrometer*, and sets the units for the positioning information of the cells in 3D.

A simple NetworkML example file using the **instance based representation** is available here: http://neuroml.org/view.php?file=NetworkML/SimpleNetworkInstance.xml.

A simple NetworkML example file using the **template based representation** is available here: http://neuroml.org/view.php?file=NetworkML/SimpleNetworkTemplate.xml.

An example of a NeuroML file containing elements from **all 3 Levels** including network structure in NetworkML is available here: http://neuroml.org/view.php?file=NetworkML/CompleteNetwork.xml.

An example of a NetworkML file containing **population**, **projection** and **input** elements can be found in Section 10.4, along with a discussion of the modeling of the network.

Element: **<populations>**

This element contains a set of one or more **population** elements, each referring to a set of cells of a specific cell type. Note that a particular cell type can be used in more than one **population**. The cells can be defined in the same or separate NeuroML files.

Element: **<population>**

A population of cells. Required attributes are: **name**: a string for referring to the population; **cell_type** for the type of cell present in the population. This can refer to the **name** attribute of a cell defined in the same file, or a cell in a (normally Level 3) morphology file which is also being/has been loaded into the parsing application. In the current version of the specification all cells in the population have identical morphological structure and electrical properties.

The **population** element can have any of the **metadata** elements as subelements.

In the instance based representation, an explicit list of cell positions are given in the **instances** element. In the template based version, the **pop_location** element is used to succinctly describe the arrangement of cells (e.g. **random_arrangement**, **grid_arrangement**).

Element: **<instances>**

This element contains a list of **instance** elements for each cell in the **population**. There is a **size** attribute which is required, even though the size can be determined by the number of **instance** elements. This is necessary since for a large network it is very useful for a parsing application to know in advance how many instances to expect for memory allocation purposes.

Element: **<instance>**

A specific **instance** in the **population** of a cell of a particular **cell_type**. Each cell will have a unique **id** attribute. There is also an optional **node_id** attribute, which refers to the computational node on which the cell is to be instantiated in a parallel computing environment. This attribute can clearly be ignored by parsing applications, but it can be convenient, e.g. for storing the network so that the exact parallel network configuration can be rerun, for visualizing the distribution of the cells on the nodes, or if the NetworkML file is to be used as a setup file when simulating the parallel network (each node can parse the file and not instantiate any cell with a **node_id** different from its own). How the distribution of cells on the nodes is actually generated is outside of the scope of NeuroML at present (and would in any case be highly dependent on the target simulator of the network). The location of the cell is given by the **location** element.

Element: **<location>**

Contains the **x**, **y**, **z** coordinates of the cell as attributes. Note these are usually expressed in micrometers, but this can be changed by the **length_units** attribute in the root element.

Element: **<pop_location>**

In the template based network representation, this is used for describing how a **population** should be created. Currently, **random_arrangement** and **grid_arrangement** are the two options for creating populations of cells.

Element: **<random_arrangement>**

A set of cells randomly placed in a 3D region. An attribute **population_size** defines the number of cell instances in the population. Currently there are two types of region defined in which the cells can be arranged. The **spherical_location** and **rectangular_location** elements are of type **Sphere** and **RectangularBox** (defined in Metadata) respectively. The parsing application must implement its own algorithm of filling these spaces, and as noted previously, it is not possible to ensure different applications will produce the same networks from these parameters. The generated networks can be stored in instance based format NetworkML though and compared directly.

Element: **<grid_arrangement>**

This element contains a subelement **non_spatial_grid** which has positive integer value attributes **x**, **y**, **z** for the number of cells in a grid in each dimension.

Element: **<projection>**

The **projections** element groups a number of individual **projection** elements. It has the attribute **units**, which will set the unit system for the synaptic delay and threshold attributes of the subelements.

Element: **<projection>**

The **projection** element defines a set of synaptic connections between a source and a target population. It has attributes **name** (a unique string), and **source** and **target**, each of which must correspond to the **name** attribute of a **population**. The source and target populations can be identical.

The **projection** element can have any of the **metadata** elements as subelements.

The **projection** element can have one or more **synapse_props** subelements. For an instance based **projection** specification, there will be a **connections** subelement with an explicit list of the synaptic connections, or for the template based case, there will be a **connectivity_pattern** subelement.

Element: **<synapse_props>**

This element is used to define the default properties for a synaptic connection of a particular type. A required attribute **synapse_type** should refer to the **name** attribute of a **synapse_type** synaptic mechanism description defined either in this Level 3 NeuroML file or in a ChannelML file. The **weight** optional attribute (1 by default) gives a multiplicative factor by which the maximal conductance of each synaptic connection should be increased. The **threshold** optional attribute (0 by default) gives the presynaptic membrane potential which when crossed causes the synaptic event. The optional attributes **internal_delay**, **pre_delay**, **post_delay** and **prop_delay** (all 0 by default) give the contribution to the delay in triggering the synaptic event due to the assumed intrinsic properties of the synapse, a delay component on the presynaptic side, a delay component on the postsynaptic side and a delay due to the propagation of an action potential along the axon when it is not explicitly simulated, respectively. All of these values can be overwritten for particular instances of the synaptic connections. Note: more than one **synapse_props** element is permitted per **projection**, as there may be channels of multiple types present at every physical synaptic connection (e.g. AMPA and NMDA receptors at glutamatergic synapses).

An example of a NetworkML file containing the **synapse_props** element can be found in Section 10.4, along with a discussion of the usage of these properties in a model of the network.

Element: **<connections>**

This contains a list of synaptic connections in **connection** elements. There is an optional **size** attribute, even though the size can be determined by the number of **connection** elements. This should be specified for large network files though, as it would be very useful for a parsing application to know in advance how many connections to expect for memory allocation purposes. This attribute is likely to be required in future versions.

Element: **<connection>**

The **connection** element refers to a single physical synaptic connection between a pre and post cell. The attribute id is a nonnegative integer unique for this **projection**. The required attribute **pre_cell_id** corresponds to the **id** attribute of an **instance** in the source population. The optional attributes **pre_segment_id** (default 0) and **pre_fraction_along** (default 0.5) refer to specific locations on the source cell. There are corresponding attributes for the postsynaptic location: **post_cell_id**, **post_segment_id** and **post_fraction_along**. For each **connection** element, there can be a number of properties elements, one for each of the **synapse_props** defined for this population. They have the same attributes as the **synapse_props** element and override the values set there, e.g. for a different weight, delay, etc. The **synapse_type** attribute is optional, but should be included if there are more than one **synapse_props** elements specified.

Element: **<connectivity_pattern>**

This element is used to define the pattern to use to set up synaptic connections between the source and target populations in a template based network specification. Currently there are three types of pattern defined: **all_to_all**, **fixed_probability** and **per_cell_connection**. Many more connection scenarios could be envisioned, and this list will be expanded in future in line with the connectivity algorithms defined by the PyNN initiative.

Element: **<all_to_all>**

This element has no attributes, and its presence signifies that every presynaptic cell should be connected to each postsynaptic cell.

Element: **<fixed_probability>**

This element has required attribute probability with value from 0 to 1, giving the probability that any specific presynaptic and postsynaptic cells will be connected.

Element: **<per_cell_connection>**

This element signals that the connections should be built iteratively from each pre (or post) cell based on a number of parameters. The **direction** attribute defines whether the connections should be built from *PreToPost* (default) or *PostToPre*. Required attribute **num_per_source** specifies the number of connections to create on each of the cells in the pre or post population from which the connections are being built. This is a positive double value, and if not an integer refers to the average number of connections in the source population. The **max_per_target** optional attribute is an integer value for the max number of connections permitted per target cell.

Element: **<inputs>**

The **inputs** element is used for the electrical stimulation to the network and contains a number of **input** elements. It has attribute **units**, which sets the unit system for the subelements.

Element: **<input>**

The **input** element defines a set of electrical inputs of a particular type on a population of cells. It has attributes **name** (a unique string), and can have any of the **metadata** elements as subelements. One of two types of electrical stimulation can be defined: **pulse_input** or **random_stim**. The **target** element defines the population to stimulate.

Element: **<pulse_input>**

This refers to an injected current pulse which is used to drive the cells. It has the following required attributes: **delay**, **duration**, **amplitude**, and the current is given by:

$$I(t) = \begin{cases} 0 & t < \text{delay} \\ \text{amplitude} & \text{delay} <= t <= \text{delay} + \text{duration} \\ 0 & t > \text{delay} + \text{duration} \end{cases}$$

Element: **<random_stim>**

This refers to a random train of synaptic inputs to be applied to locations on the cells. The required attribute **frequency** refers to the spike triggering frequency, and **synaptic_mechanism** refers to the **name** attribute of a synaptic mechanism defined either in this (NeuroML Level 3) file or in a ChannelML file, which is used to provide the conductance change with time. The spike events should be independent of one another, leading to a Poisson distribution for the number of spiking events over time. There is currently no refractory period specified.

Element: **<target>**

The target element has attribute **population**, linked to the **name** attribute of a defined **population**. The subelements here are either: **sites** in an instance based network description, or **site_pattern** for a template based network file.

Element: **<sites>**

This contains a list of sites to which to apply the defined electrical stimulations in **site** elements. There is an optional **size** attribute, and while the size can be determined by the number of **site** elements, this should be specified, as it may be useful for a parsing application to know in advance how many stimulation sites to expect for memory allocation purposes.

Element: **<site>**

This element has the required attribute **cell_id** for the **id** of the cell in the **population** to which to apply this instance of the stimulus. The optional attributes **segment_id** (default 0) and **fraction_along** (default 0.5) can be used for greater specificity in location of stimulation.

Element: **<site_pattern>**

This element for the template based specification of the input stimuli can have one of the following possible subelement: **all_cells** and **percentage_cells**.

Element: **<all_cells >**

The presence of this element signals that all of the cells in population should have the input applied to them.

Element: **<percentage_cells >**

This element has attribute **percentage** indicating the percentage of cells in the population to which to apply the input.

10. Example NeuroML files

10.1 Spiking single compartment cell model

The following Level 2 file contains a single compartment/segment cell with Na^+ and K^+ and leak conductances based on the Hodgkin and Huxley squid axon model (Hodgkin and Huxley, 1952).

```
<neuroml xmlns = "http://morphml.org/neuroml/schema"
    xmlns:meta = "http://morphml.org/metadata/schema"
    xmlns:mml = "http://morphml.org/morphml/schema"
    xmlns:bio = "http://morphml.org/biophysics/schema"
    xmlns:xsi = "http://www.w3.org/2001/XMLSchema-instance"
    xsi:schemaLocation = "http://morphml.org/neuroml/schema  NeuroML.xsd"
    lengthUnits = "micrometer">

    <cells>
        <cell name = "HH_Cell">

            <meta:notes>A Simple cell with HH channels</meta:notes>

            <mml:segments>
                <mml:segment id = "0" name = "Soma" cable = "0">
                    <mml:proximal x = "0.0" y = "0.0" z = "0.0" diameter = "16.0"/>
                    <mml:distal x = "0.0" y = "10.0" z = "0.0" diameter = "16.0"/>
                </mml:segment>
            </mml:segments>

            <mml:cables>
                <mml:cable id = "0" name = "Soma">
                    <meta:group>all</meta:group>
                    <meta:group>soma_group</meta:group>
                </mml:cable>
            </mml:cables>

            <!-- Adding the biophysical parameters -->

            <biophysics units = "Physiological Units">

                <bio:mechanism name = "KConductance" type = "Channel Mechanism">
                    <bio:parameter name = "gmax" value = "36.0">
                        <bio:group>all</bio:group>
                    </bio:parameter>
                </bio:mechanism>

                <bio:mechanism name = "NaConductance" type = "Channel Mechanism">
                    <bio:parameter name = "gmax" value = "120.0">
                        <bio:group>all</bio:group>
                    </bio:parameter>
                </bio:mechanism>

                <bio:mechanism name="LeakCond" type="Channel Mechanism" passive_conductance="true">
                    <bio:parameter name = "e" value = "-54.3">
                        <bio:group>all</bio:group>
                    </bio:parameter>
                    <bio:parameter name = "gmax" value = "0.3">
                        <bio:group>all</bio:group>
                    </bio:parameter>
                </bio:mechanism>

                <bio:spec_capacitance>
                    <bio:parameter value = "1.0">
                    <bio:group>all</bio:group>
                    </bio:parameter>
                </bio:spec_capacitance>

                <bio:spec_axial_resistance>
                    <bio:parameter value = "0.1">
                        <bio:group>all</bio:group>
                    </bio:parameter>
                </bio:spec_axial_resistance>

            </biophysics>

        </cell>
    </cells>

</neuroml>
```

The cell consists of a single cylinder (**segment** with **id** = *0*) of length 10μm and diameter 16μm giving a curved surface area of 502.65 μm² . One cable is present and this is used to indicate that the segment is part of the *soma_group*.

The **biophysics** element contains details of the passive and active electrical properties of the cell. The units of all quantities inside this element are set to be *Physiological Units*. Three channel mechanisms are present: *KConductance* (with conductance density of 36 mS cm^{-2} (360 S m^{-2})), *NaConductance* (120 mS cm^{-2} (1200 S m^{-2})) and *LeakCond* (0.3 mS cm^{-2} (3 S m^{-2})). While the description of *KConductance* and *NaConductance* will be contained in ChannelML files (see example 10.2), none is required for *LeakCond*, as this specifies **passive_conductance** = *true* and so the complete description of this conductance is determined by the parameters for reversal potential (*e* = -54.3 mV), and conductance density (*gmax*). Specific capacitance is set at 1 μF cm^{-2} (0.01 F m^{-2}) and specific axial resistance is set to 0.1 kohm cm (1 ohm m).

We will assume that given the small size of the cell, it is isopotential (and so specific axial resistance is not required in the subsequent discussion). The equation determining the behaviour of the membrane potential V_m of the cell is:

$$C\frac{dV_m}{dt} + G_{leak}(V_m,t)\cdot(V_m - E_{leak}) + G_{Na}(V_m,t)\cdot(V_m - E_{Na}) + G_K(V_m,t)\cdot(V_m - E_K) = I_{ext}(t)$$

where C is the total capacitance of the compartment (**spec_capacitance** * surface area), E_{leak} is the reversal potential of the leak conductance (parameter *e* in mechanism *LeakCond*), E_{Na} and E_K are the reversal potentials obtained from the ChannelML files describing the Na^+ and K^+ conductances respectively (see example 10.2; note that these default reversal potential values in the ChannelML file can be overwritten by using an **ion_props** element in **biophysics**).

$G_{leak}(V_m,t)$, $G_{Na}(V_m,t)$ and $G_K(V_m,t)$ are the total conductances of the leak, Na^+ and K^+ conductances respectively:

$$G_A(V_m,t) = g_A(V_m,t)\cdot(\text{membrane surface area})$$

Note that $g_{Leak}(V_m,t) = gmax_{Leak}$ i.e. does not vary with time or membrane potential. $g_{Na}(V_m,t)$ and $g_K(V_m,t)$ can be determined from the respective ChannelML files as outlined in section 10.2.

$I_{ext}(t)$ is added to the above equation to indicate how an external current (e.g. due to an current clamp electrode or synaptic input) would contribute to the behavior of the cell membrane potential (note that such a current would be added in an **input** element specifying a **pulse_input** in a Level 3 network file).

A good introduction to the cable theory of dendrites is contained in chapter 2 of (Koch and Segev, 1998), and chapter 3 gives a background on how such structures might be represented by compartmental models.

Chapters 5 and 6 of (Dayan and Abbott, 2001) also contain a thorough description of the modeling of single and multicompartment neurons and is well integrated with the rest of the book on theoretical approaches to computational neuroscience.

A detailed discussion on the physiological processes underlying single cell electrical behavior and the models used to describe these is contained in (Koch, 1999).

Simulator implementations:

Chapter 5 of the GENESIS book (Bower and Beeman, 1997) also gives an introduction to cable modeling (available here: http://www.genesis-sim.org/GENESIS/iBoG/iBoGpdf), and later chapters, including chapter 15 discuss the specifics of how GENESIS uses compartments to simulate such structures.

NEURON's use of the section object for cable modeling is explained in chapter 5 of the NEURON Book (Carnevale and Hines, 2006).

A different approach to modeling conductances spread across a membrane's surface is taken by PSICS. This simulator's use of detailed neuronal morphologies is explained here: http://www.psics.org/formats/morphology.html and its handling of cellular electrical properties is outlined here: http://www.psics.org/formats/properties.html.

10.2 Na^+ conductance in ChannelML

The following ChannelML file shown below contains a description of a Na^+ conductance based on (Hodgkin and Huxley, 1952). See Figure 3 in the main NeuroML paper for an example of the squid axon K^+ conductance.

```
<channelml
    xmlns = "http://morphml.org/channelml/schema"
    xmlns:xsi = "http://www.w3.org/2001/XMLSchema-instance"
    xmlns:meta = "http://morphml.org/metadata/schema"
    xsi:schemaLocation = "http://morphml.org/channelml/schema  ChannelML_v1.8.1.xsd"
    units = "Physiological Units">

    <meta:notes>ChannelML file containing a single Channel description</meta:notes>

    <channel_type name = "NaConductance">

        <status value = "stable"/>

        <meta:notes>Simple example of Na+ conductance in squid giant axon. Based on Hodgkin and
                    Huxley 1952</meta:notes>

        <current_voltage_relation cond_law="ohmic" ion="na" default_erev="50" default_gmax="120">

            <gate name = "m" instances = "3">
                <closed_state id = "m0"/>
                <open_state id = "m"/>

                <transition name = "alpha" from = "m0" to = "m" expr_form = "exp_linear"
                            rate = "1" scale = "10" midpoint = "-40"/>
                <transition name = "beta" from = "m" to = "m0" expr_form = "exponential"
                            rate = "4" scale = "-18" midpoint = "-65"/>
            </gate>

            <gate name = "h" instances = "1">
                <closed_state id = "h0"/>
                <open_state id = "h"/>

                <transition name = "alpha" from = "h0" to = "h" expr_form = "exponential"
                            rate = "0.07" scale = "-20" midpoint = "-65"/>
                <transition name = "beta" from = "h" to = "h0" expr_form = "sigmoid"
                            rate = "1" scale = "-10" midpoint = "-35"/>
            </gate>

        </current_voltage_relation>


    </channel_type>
</channelml>
```

In the root **channelml** element, the units of all subsequent elements are set to be *Physiological Units*. In the **current_voltage_relation** element, the conductance law is set to *ohmic*, the ion flowing through the channel is specified as *na*, the default reversal potential is 50 mV and the default conductance density is 120 mS cm^{-2}.

Given that there are 2 gate elements, *m* and *h* with 3 and 1 **instances** respectively, the equation for the conductance density of the channel on a compartment with membrane potential V_m will be:

$$g_{Na}(V_m,t) = gmax_{Na} \cdot m(V_m,t)^3 \cdot h(V_m,t)$$

and so the current per unit area will be:

$$I_{Na}(V_m,t) = g_{Na}(V_m,t) \cdot (V_m - E_{Na})$$

As mentioned in section 7, the **default_gmax** (for $gmax_{Na}$) and **default_erev** (E_{Na}) are optional attributes. The conductance density of a channel will be overwritten in element **mechanism** in **biophysics** when the channel is placed on a cell, and E_{Na} can be overwritten with the **ion_props** element.

The state variables of the channel, $m(V_m,t)$ and $h(V_m,t)$, will be determined by the forward (**closed_state** to **open_state**, $alpha_m$ & $alpha_h$) and reverse (**open_state** to **closed_state**, $beta_m$ & $beta_h$) transition rates:

$$\frac{dm(V_m,t)}{dt} = alpha_m(V_m)\cdot(1-m) - beta_m(V_m)\cdot m$$

$$\frac{dh(V_m,t)}{dt} = alpha_h(V_m)\cdot(1-h) - beta_h(V_m)\cdot h$$

The form of the equations for $alpha_m$, $beta_m$ etc. will be set by the value of the **expr_form** attribute, and have the following forms (see definition of **transition** element in section 7):

$$alpha_m(V_m) = \frac{rate \times \frac{V_m - midpoint}{scale}}{1-e^{-1\times\frac{V_m - midpoint}{scale}}} = \frac{1\cdot\frac{V_m-(-40)}{10}}{1-e^{-1\times\frac{V_m-(-40)}{10}}}$$

$$beta_m(V_m) = rate \times e^{\frac{V_m - midpoint}{scale}} = 4 \times e^{\frac{V_m-(-65)}{-18}}$$

$$alpha_h(V_m) = rate \times e^{\frac{V_m - midpoint}{scale}} = 0.07 \times e^{\frac{V_m-(-65)}{-20}}$$

$$beta_h(V_m) = \frac{rate}{1+e^{\frac{V_m - midpoint}{scale}}} = \frac{1}{1+e^{\frac{V_m-(-35)}{-10}}}$$

The 3 references given at the start of the box in section 10.1 are very useful for explanations of the theory behind the Hodgkin Huxley model. They also discuss how the basic model can be extended to incorporate the wide range of active Na^+, K^+ and Ca^{2+} conductances and interactions with subcellular $[Ca^{2+}]$.

Simulator implementations:

Chapter 4 of the GENESIS book (Bower and Beeman, 1997) also gives an introduction to the Hodgkin Huxley model, and chapter 7 discusses some of the other types of ion channel commonly used in conductance based models. Chapter 13 presents how these types of channels are modeled in GENESIS. The GENESIS script for the above example of a Na^+ channel using the tabchannel object (http://www.genesis-sim.org/GENESIS/Hyperdoc/Manual-26.html#ss26.62) is available here: http://neuroml.org/view.php?file=ChannelML/NaChannel_HH.xml&map=GENESIS.

Implementation of active membrane conductances using NEURON's NMODL language (http://www.neuron.yale.edu/neuron/static/papers/nc2000/nmodl.htm) is discussed in chapter 9 of the of the NEURON Book (Carnevale and Hines, 2006). The NMODL script for the Na^+ channel described above is here: http://neuroml.org/view.php?file=ChannelML/NaChannel_HH.xml&map=NEURON.

A discussion of the PSICS approach to channel modeling is available here: http://www.psics.org/formats/channels.html. The PSICS representation of the Na^+ channel is here: http://neuroml.org/view.php?file=ChannelML/NaChannel_HH.xml&map=PSICS.

10.3 Double exponential conductance waveform synapse

The following ChannelML file describes a synaptic mechanism with a double exponential waveform for the time varying conductance.

```
<channelml
    xmlns = "http://morphml.org/channelml/schema"
    xmlns:xsi = "http://www.w3.org/2001/XMLSchema-instance"
    xmlns:meta = "http://morphml.org/metadata/schema"
    xsi:schemaLocation = "http://morphml.org/channelml/schema  ChannelML_v1.8.1.xsd"
    units = "Physiological Units">

    <meta:notes>ChannelML file containing a single synapse description</meta:notes>

    <synapse_type name = "DoubleExpSynapse">

        <status value = "stable"/>

        <meta:notes>Simple double exponential waveform synapse</meta:notes>

        <doub_exp_syn max_conductance="1.0E-5" rise_time="1" decay_time="2" reversal_potential="0"/>

    </synapse_type>
</channelml>
```

In the root **channelml** element, the units of all subsequent elements are set to be *Physiological Units*. The synapse is ohmic and the current into a compartment with membrane potential V_m due to the conductance of the synapse ($G_{syn}(t)$) which has reversal potential E_{syn} (**reversal_potential** above, 0 mV) will be given by:

$$I_{syn}(V_m, t) = G_{syn}(t) \cdot (V_m - E_{syn})$$

The time varying conductance following a synaptic firing event at t =0 is given by:

$$G_{syn}(t) = G_{max} \cdot A \cdot \left(e^{\frac{-t}{decay\_time}} - e^{\frac{-t}{rise\_time}} \right) \qquad \text{for } t >= 0$$

where G_{max} is the maximal conductance the synapse reaches on a single synaptic event (**max_conductance** above, 1e-5 mS) and where the normalization factor A is given by

$$A = \frac{1}{e^{-\frac{peak\_time}{decay\_time}} - e^{-\frac{peak\_time}{rise\_time}}}$$

and peak_time, the time to reach maximum conductance, is:

$$peak\_time = \frac{(decay\_time \cdot rise\_time)}{(decay\_time - rise\_time)} \cdot \ln\left(\frac{decay\_time}{rise\_time}\right)$$

Note that if **rise_time** was zero this would simplify to a single exponential synapse:

$$G_{syn}(t) = max\_conductance \cdot e^{\frac{-t}{decay\_time}} \qquad \text{for } t >= 0$$

Note also if **decay_time** = **rise_time** = alpha_time, the waveform is for an alpha synapse with peak at alpha_time:

$$G_{syn}(t) = max\_conductance \cdot \left(\frac{t}{alpha\_time}\right) \cdot e^{\left(1 - \frac{t}{alpha\_time}\right)} \qquad \text{for } t >= 0$$

Models of synaptic conductances are discussed in chapters 5 of (Dayan and Abbott, 2001) and in chapter 4 of (Koch, 1999).

Simulator implementations:

An introduction to the modelling of synaptic conductances can be found in chapter 6 of the GENESIS book (Bower and Beeman, 1997), and chapter 14 discusses the specifics of how this is implemented in GENESIS.

Chapter 10 of the NEURON Book (Carnevale and Hines, 2006) also discusses modeling of synaptic transmission.

10.4 Simple Network

The NetworkML file below contains a simple network of consisting of 2 populations of cells, a network connection between them and an external synaptic input to the presynaptic population.

```
<networkml xmlns = "http://morphml.org/networkml/schema"
           xmlns:xsi = "http://www.w3.org/2001/XMLSchema-instance"
           xmlns:meta = "http://morphml.org/metadata/schema"
           xsi:schemaLocation = "http://morphml.org/networkml/schema NetworkML_v1.8.1.xsd"
           length_units = "micrometer">

    <meta:notes>A simple network where instances of cell populations, connections,
    etc. are specified. </meta:notes>

    <populations>
        <population name = "PopA" cell_type = "HH_Cell">
            <instances size = "2">  <!--  All instances listed -->
                <instance id = "0"><location x = "0" y = "0" z = "0"/></instance>
                <instance id = "1"><location x = "100" y = "0" z = "0"/></instance>
            </instances>
        </population>
        <population name = "PopB" cell_type = "HH_Cell">
            <instances size = "3">  <!--  All instances listed -->
                <instance id = "0"><location x = "0" y = "100" z = "0"/></instance>
                <instance id = "1"><location x = "100" y = "100" z = "0"/></instance>
                <instance id = "2"><location x = "200" y = "100" z = "0"/></instance>
            </instances>
        </population>
    </populations>


    <projections units = "Physiological Units">
        <projection name = "NetworkConnection" source = "PopA" target = "PopB">

            <synapse_props synapse_type = "DoubleExpSynapse" internal_delay = "5" threshold = "-20"/>

            <connections><!--  All connection instances specified -->
                <connection id = "0" pre_cell_id = "0" post_cell_id = "1"/>
                <connection id = "1" pre_cell_id = "1" post_cell_id = "0">
                    <properties weight = "0.5"/>        <!--  adjusted value  -->
                </connection>
            </connections>

        </projection>
    </projections>


    <inputs units = "SI Units">

        <input name = "RandomInput">
            <random_stim frequency = "50" synaptic_mechanism = "DoubleExpSynapse"/>
            <target population = "PopA">
                <sites>
                    <site cell_id = "1"/>
                    <site cell_id = "2"/>
                </sites>
            </target>
        </input>

    </inputs>

</networkml>
```

It uses the instance based form of NetworkML and so all of the positions, connections and input locations are explicitly listed.

The **populations**, *PopA* and *PopB* are both made up of instances of a cell of type *HH_Cell*. The populations consist of 2 and 3 cells respectively and the precise locations in 3D Cartesian coordinates are given.

The **projection** specifies *PopA* as the presynaptic population and *PopB* as the postsynaptic population. The type of the synaptic mechanism is specified in **synapse_props** as *DoubleExpSynapse*. As with the reference to *HH_Cell*, the implementation of *DoubleExpSynapse* will be in an external NeuroML file.

There is an **internal_delay** of 5 ms specified for all instances of connections in this projection (this could be used to model the delay associated with propagation of the action potential along an axon, or the time taken for vesicles to release and the neurotransmitter to cross the synapse). The **threshold** at which the presynaptic location should be considered spiking is set at -20 mV. Two **connection** instances are present. The second **connection** has a different **weight** associated with it, and a scaling factor of 0.5 will be applied to the conductance of the synapse as described in the original *DoubleExpSynapse.*

The logical sequence associated with a spiking event in this model is:

1) The membrane voltage of both the pre and postsynaptic cells evolve with time according to the electrical properties of their membranes (sections 10.1 and 10.2).

2) When a "listener" at the presynaptic connection location sees that the membrane potential passes **threshold**, a spiking event is registered with an object at the postsynaptic location which is responsible for adding the synaptic current. No further spiking events will be transmitted until the presynaptic membrane potential has again passed below the **threshold** value (note this time will be greater than zero in the continuous HH case). The behavior of the presynaptic cell is not otherwise affected by the spiking event.

3) The postsynaptic object for the synapse will wait for a time **internal_delay** before applying the conductance change (potentially scaled by a global or synapse specific factor **weight**). The time course of the conductance will be calculated as outlined in section 10.3. Note that the synaptic conductance may reach a value higher than **max_conductance**, if the conductance was already non zero due to a previous spiking event.

4) The membrane potential of the postsynaptic cell will then evolve according to the first equation in section 10.1, with $I_{ext}(t)$ being the sum of currents due to synaptic inputs.

The **input** on the presynaptic **population** specifies that a **random_stim** is applied, which consists of a Poisson process with an average **frequency** of 50 Hz (note SI Units specified in **inputs**) triggering a *DoubleExpSynapse* onto each of the 2 listed input sites. This current too should be incorporated into the $I_{ext}(t)$ term in the first equation in section 10.1 to determine the evolution of the voltage of the presynaptic cell.

Chapters 10 and 12 of (Koch and Segev, 1998) give detailed descriptions of network model containing conductance based cell models.

Simulator implementations:

Chapter 17 of the GENESIS book (Bower and Beeman, 1997), describes the setup of a network of cells using its inbuilt objects.

Chapter 11 of the NEURON Book (Carnevale and Hines, 2006) presents the commands and objects it supports for network modeling.

11. References

Bower, J.M., and Beeman, D. (1997). The Book of GENESIS: Exploring Realistic Neural Models with the GEneral NEural SImulation System (Springer, New York).

Brette, R., Rudolph, M., Carnevale, T., Hines, M., Beeman, D., Bower, J.M., Diesmann, M., Morrison, A., Goodman, P.H., Harris, F.C., Jr., *et al.* (2007). Simulation of networks of spiking neurons: a review of tools and strategies. J Comput Neurosci *23*, 349-398.

Carnevale, N.T., and Hines, M.L. (2006). The NEURON Book (Cambridge University Press).

Crook, S., Gleeson, P., Howell, F., Svitak, J., and Silver, R.A. (2007). MorphML: Level 1 of the NeuroML standards for neuronal morphology data and model specification. Neuroinformatics *5*, 96-104.

Davison, A.P., Bruderle, D., Eppler, J., Kremkow, J., Muller, E., Pecevski, D., Perrinet, L., and Yger, P. (2008). PyNN: A Common Interface for Neuronal Network Simulators. Front Neuroinformatics *2*, 11.

Dayan, P., and Abbott, L.F. (2001). Theoretical neuroscience : computational and mathematical modeling of neural systems (Cambridge, Mass., Massachusetts Institute of Technology Press).

Gabbiani, F., Midtgaard, J., and Knopfel, T. (1994). Synaptic integration in a model of cerebellar granule cells. J Neurophysiol *72*, 999-1009.

Gleeson, P., Steuber, V., and Silver, R.A. (2007). neuroConstruct: A Tool for Modeling Networks of Neurons in 3D Space. Neuron *54*, 219-235.

Hille, B. (2001). Ion channels of excitable membranes, 3rd ed. (Sunderland, MA, Sinauer Associates).

Hodgkin, A.L., and Huxley, A.F. (1952). A quantitative description of membrane current and its application to conduction and excitation in nerve. J Physiol *117*, 500-544.

Hucka, M., Finney, A., Sauro, H.M., Bolouri, H., Doyle, J.C., Kitano, H., Arkin, A.P., Bornstein, B.J., Bray, D., Cornish-Bowden, A., *et al.* (2003). The systems biology markup language (SBML): a medium for representation and exchange of biochemical network models. Bioinformatics *19*, 524-531.

Jahr, C.E., and Stevens, C.F. (1990). Voltage dependence of NMDA-activated macroscopic conductances predicted by single-channel kinetics. J Neurosci *10*, 3178-3182.

Koch, C. (1999). Biophysics of computation : information processing in single neurons (New York, Oxford University Press).

Koch, C., and Segev, I. (1998). Methods in neuronal modeling : from ions to networks, 2nd edn (Cambridge, Mass., MIT Press).

Maex, R., and De Schutter, E.D. (1998). Synchronization of golgi and granule cell firing in a detailed network model of the cerebellar granule cell layer. J Neurophysiol *80*, 2521-2537.

Song, S., Miller, K.D., and Abbott, L.F. (2000). Competitive Hebbian learning through spike-timing-dependent synaptic plasticity. Nat Neurosci *3*, 919-926.

Traub, R.D., Contreras, D., Cunningham, M.O., Murray, H., LeBeau, F.E., Roopun, A., Bibbig, A., Wilent, W.B., Higley, M.J., and Whittington, M.A. (2005). Single-column thalamocortical network model exhibiting gamma oscillations, sleep spindles, and epileptogenic bursts. J Neurophysiol *93*, 2194-2232.

Tsodyks, M., Uziel, A., and Markram, H. (2000). Synchrony generation in recurrent networks with frequency-dependent synapses. J Neurosci *20*, RC50.

Tsodyks, M.V., and Markram, H. (1997). The neural code between neocortical pyramidal neurons depends on neurotransmitter release probability. Proc Natl Acad Sci U S A *94*, 719-723.
