# Supplementary material for: NeuroML: A Language for Describing Data Driven Models of Neurons and Networks with a High Degree of Biological Detail
Source: PLoS Comput Biol. 2010 Jun 17;6(6):e1000815. doi: 10.1371/journal.pcbi.1000815 (PMC2887454; doi:10.1371/journal.pcbi.1000815)
Supplement: Figure S1 — Single compartment cell with 6 channels from CA1 pyramidal cell on NEURON, GENESIS, MOOSE and PSICS (0.08 MB PDF) [file pcbi.1000815.s002.pdf]

Supporting Figure S1: Single compartment cell model with channels from CA1 pyramidal cell

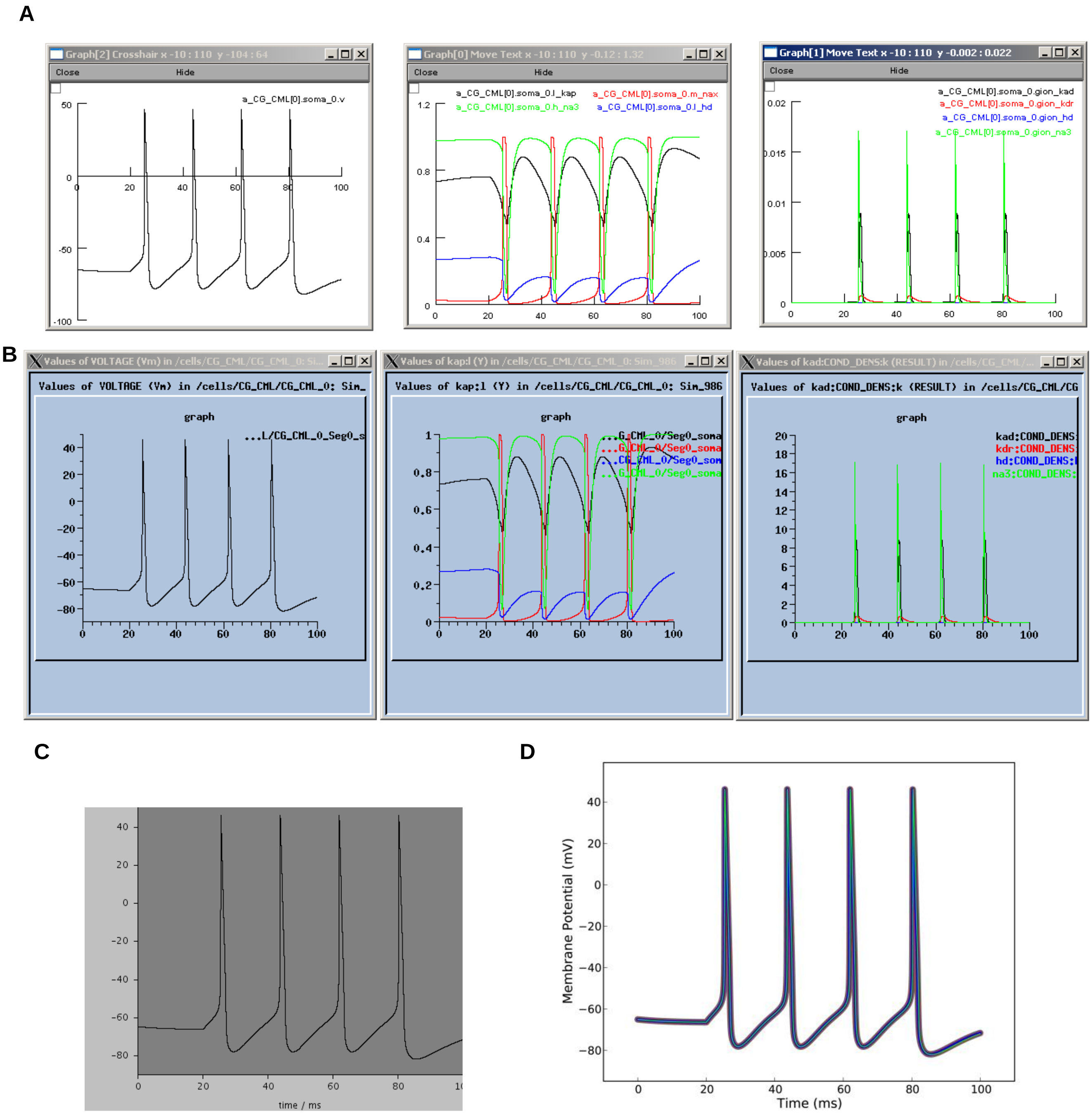

Figure S1: Single compartment cell with 6 channels from CA1 pyramidal cell on NEURON, GENESIS, MOOSE and PSICS. A) Screenshots of cell behavior on NEURON. Traces are shown of membrane potential (left), a number of internal state variables of the channels (inactivation variable l of kap (black), activation variable m of nax (red), inactivation variable l of hd (blue), inactivation variable h of na3 (green), middle plot) and conductances of kad (black), kdr (red), hd (blue) and na3 (green) on the right over 100 ms. B) Behavior of cell on GENESIS with equivalent traces to A. C) PSICS generated image of membrane potential after execution of the model on that simulator. D) Overlaid traces of membrane potential from NEURON (grey) , GENESIS (red) , MOOSE (blue) and PSICS (green).
