# Supplementary material for: NeuroML: A Language for Describing Data Driven Models of Neurons and Networks with a High Degree of Biological Detail
Source: PLoS Comput Biol. 2010 Jun 17;6(6):e1000815. doi: 10.1371/journal.pcbi.1000815 (PMC2887454; doi:10.1371/journal.pcbi.1000815)
Supplement: Figure S2 — Layer 2/3 Fast Rhythmic Bursting Pyramidal cell as exported from NEURON and converted to 3D in neuroConstruct (0.10 MB PDF) [file pcbi.1000815.s003.pdf]

Supporting Figure S2: Layer 2/3 Fast Rhythmic Bursting Pyramidal cell in 3D

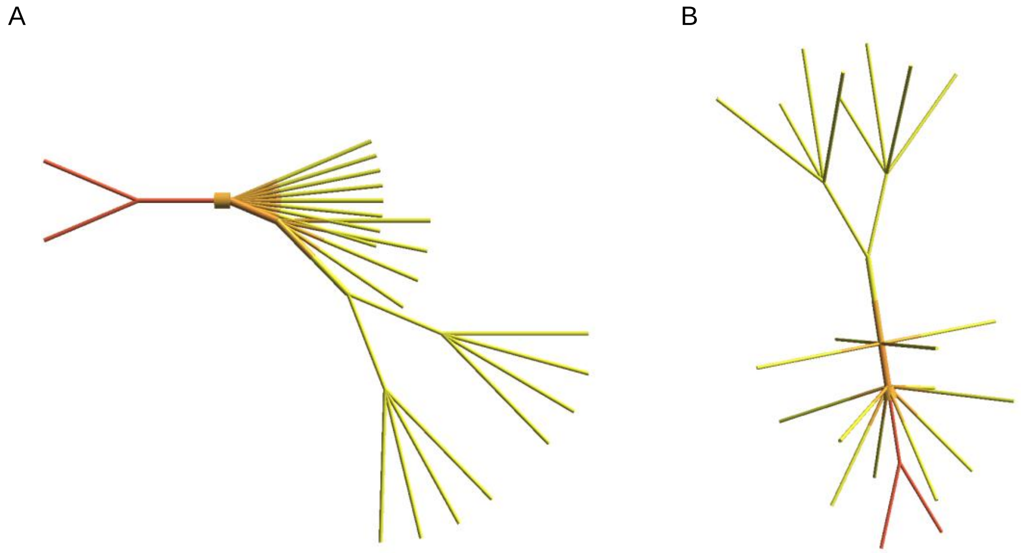

Figure S2: Layer 2/3 Fast Rhythmic Bursting Pyramidal cell as exported from NEURON and converted to 3D in neuroConstruct. A) Automatically generated 2D structure from NEURON model. Note that the original Traub et al 2005 model did not require full 3D positions for the dendrites, etc. (the network was a 1D representation of a cortical column) and so when the model was translated to NEURON, only information on the lengths and connections between sections was supplied. These 2D points were generated from this connectivity information. B) After conversion to 3D in neuroConstruct. Locations of channel naf are shown in both cases (4.0E-6 mS μm^{-2} (red), 2.5e-7 mS μm^{-2} (yellow)). Note the segments have been given a minimum radius of 2μm to aid visualizations.
