# Supplementary material for: NeuroML: A Language for Describing Data Driven Models of Neurons and Networks with a High Degree of Biological Detail
Source: PLoS Comput Biol. 2010 Jun 17;6(6):e1000815. doi: 10.1371/journal.pcbi.1000815 (PMC2887454; doi:10.1371/journal.pcbi.1000815)
Supplement: Figure S3 — Behavior of 10 cell models from Traub et al., 2005 on NEURON, GENESIS and MOOSE (0.55 MB PDF) [file pcbi.1000815.s004.pdf]

Supporting Figure S3: Behavior of 10 cell models from Traub et al., 2005 on NEURON, GENESIS and MOOSE

Cell 1: L23PyrRS

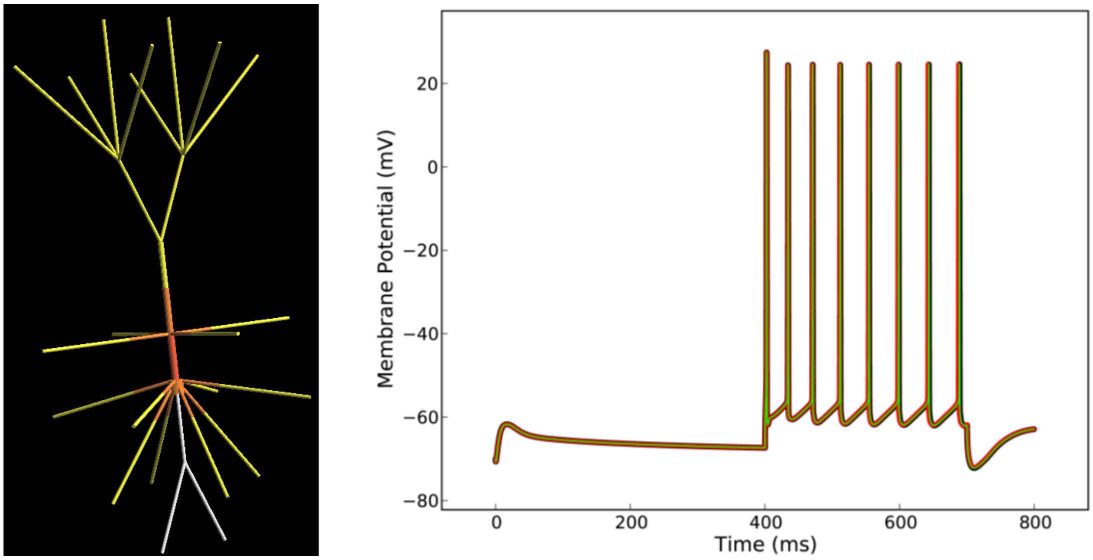

Figure S3i: Layer 2/3 Regular Spiking Pyramidal cell showing locations of persistent Na^+ conductances (left; low conductance: yellow, high conductance: red, none present: white). The plot on the right shows simulated behavior on NEURON (black, CVODE variable timestep method), GENESIS (red, 0.005 ms timestep) and MOOSE (green, 0.005 ms timestep) on application of hyperpolarizing current (-0.4 nA, pre 400 ms) followed by a depolarizing current (0.75 nA for 300 ms). The difference in time of the last spike is 1.8 ms (0.25% spike timing shift, i.e. the biggest difference in the final spike time between the 3 simulators as a percentage of the time to the first of these 3 spikes). There were 3755 numerical integration points in the NEURON simulation (total nseg).

Cell 2: L23PyrFRB

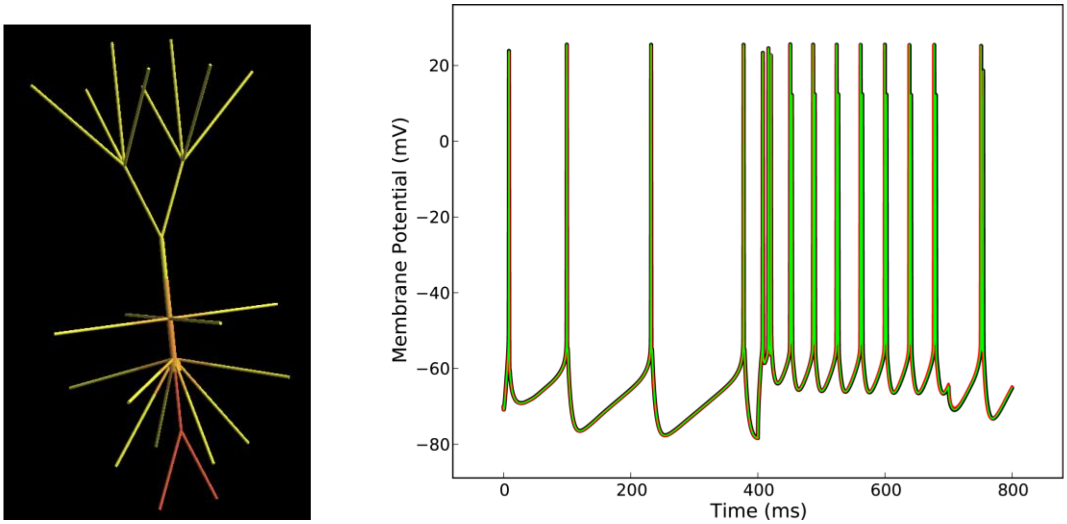

Figure S3ii: Layer 2/3 Fast Rhythmic Bursting Pyramidal cell showing locations of fast Na^+ conductances (left). Cell has same morphology as L23PyrRS, but a different set of conductances. Plot on the right shows simulated behavior on NEURON (red, CVODE variable timestep method), GENESIS (red, 0.005 ms timestep) and MOOSE (green, 0.005 ms timestep) on application of a hyperpolarizing current (-0.6 nA pre 400 ms) followed by a depolarizing current (0.4 nA for 300 ms). The difference in the timing of the last spike is 1.3 ms (0.18% spike timing shift). There were 758 numerical integration points in the NEURON simulation (total nseg).

Cell 3: SupBasket

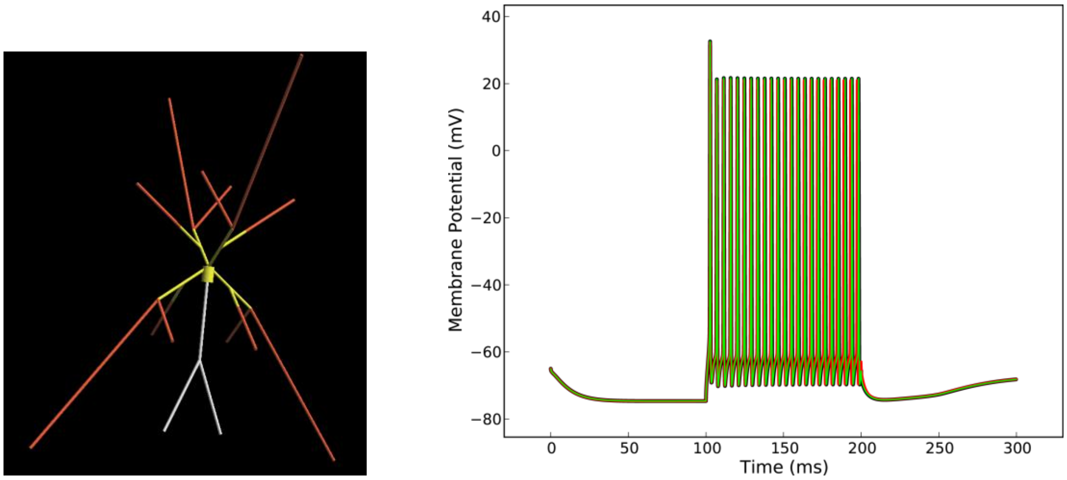

Figure S3iii: Superficial Basket interneuron showing locations of L-type Ca^{2+} conductances (left). Plot on the right shows simulated behavior on NEURON (black, CVODE variable timestep method), GENESIS (red, 0.005 ms timestep) and MOOSE (green, 0.005 ms timestep) on application of hyperpolarizing current (-0.1 nA pre 100ms) followed by a depolarizing current (0.4 nA for 100ms). The difference in the timing of the last spike is 0.97 ms (0.49% spike timing shift). There were 573 numerical integration points in the NEURON simulation (total nseg).

Note that **Cell 4: SupAxAx**, **Cell 10: DeepBasket** and **Cell 11: DeepAxAx** have identical morphologies and electrophysiological properties as this cell (the basket and axo-axonic interneurons are connected differently in the network).

Cell 5: SupLTSInter

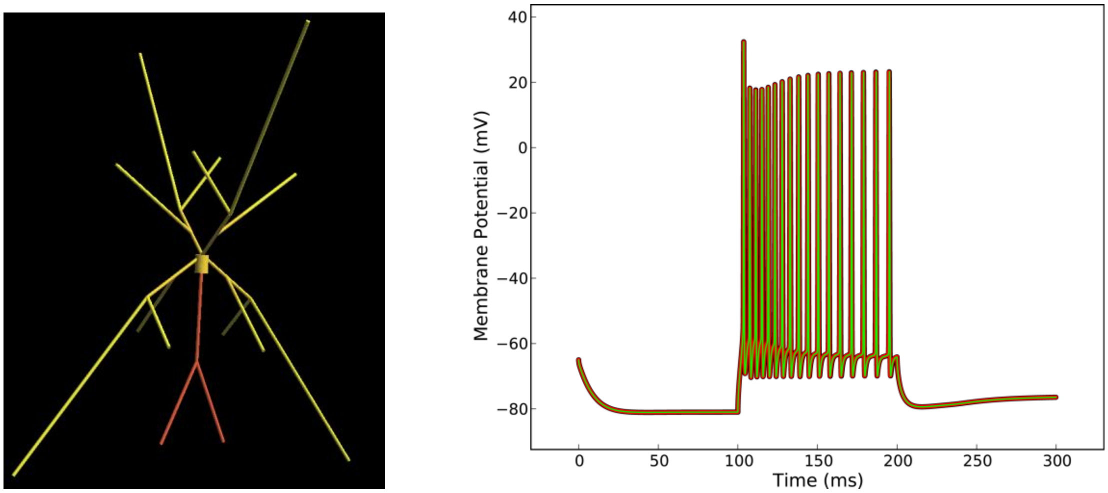

Figure S3iv: Superficial Low Threshold Spiking interneuron showing locations of delayed rectifier potassium conductances (left). The plot on the right shows simulated behavior on NEURON (black, CVODE variable timestep method), GENESIS (red, 0.002 ms timestep) and MOOSE (green, 0.002 ms timestep) on application of hyperpolarizing current (-0.15 nA pre 100ms) followed by a depolarizing current (0.4 nA for 100ms). The difference in the timing of the last spike is 0.29 ms (0.15% spike timing shift). There were 5555 numerical integration points in the NEURON simulation (total nseg).

Note: **Cell 12: DeepLTSInter** has identical morphology and electrophysiological properties as this cell.

Cell 6: L4SpinyStellate

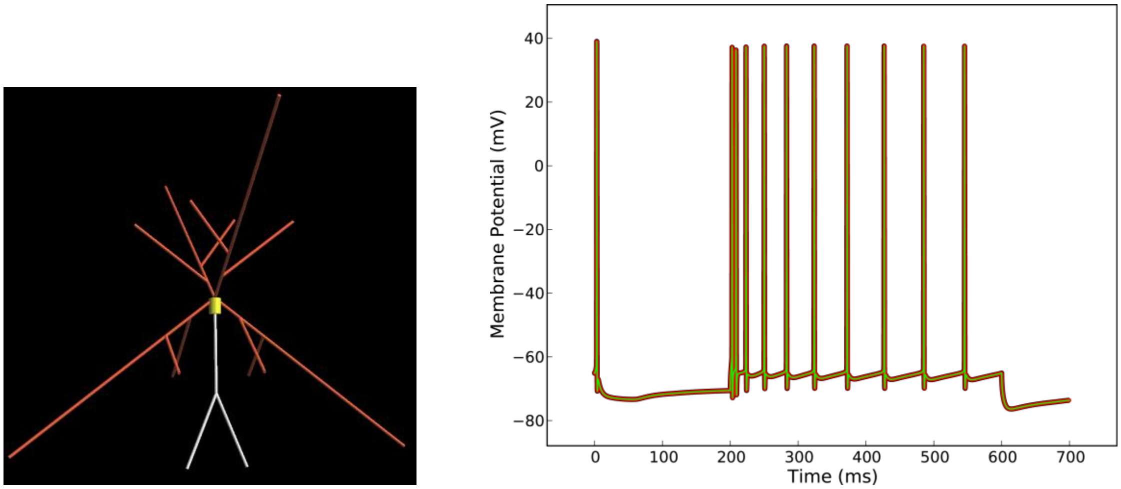

Figure S3v: Layer 4 Spiny Stellate cell showing locations of anomalous rectifier potassium conductance (left). Plot on the right shows simulated behavior on NEURON (black, CVODE variable timestep method), GENESIS (red, 0.0025 ms timestep) and MOOSE (green, 0.0025 ms timestep) on application of a hyperpolarizing current (-0.05 nA pre 200ms) followed by a depolarizing current (0.333 nA for 400ms). The difference in the timing of the last spike is 0.24 ms (0.04% spike timing shift). There were 11924 numerical integration points in the NEURON simulation (total nseg).

Cell 7: L5TuftedPyrIB

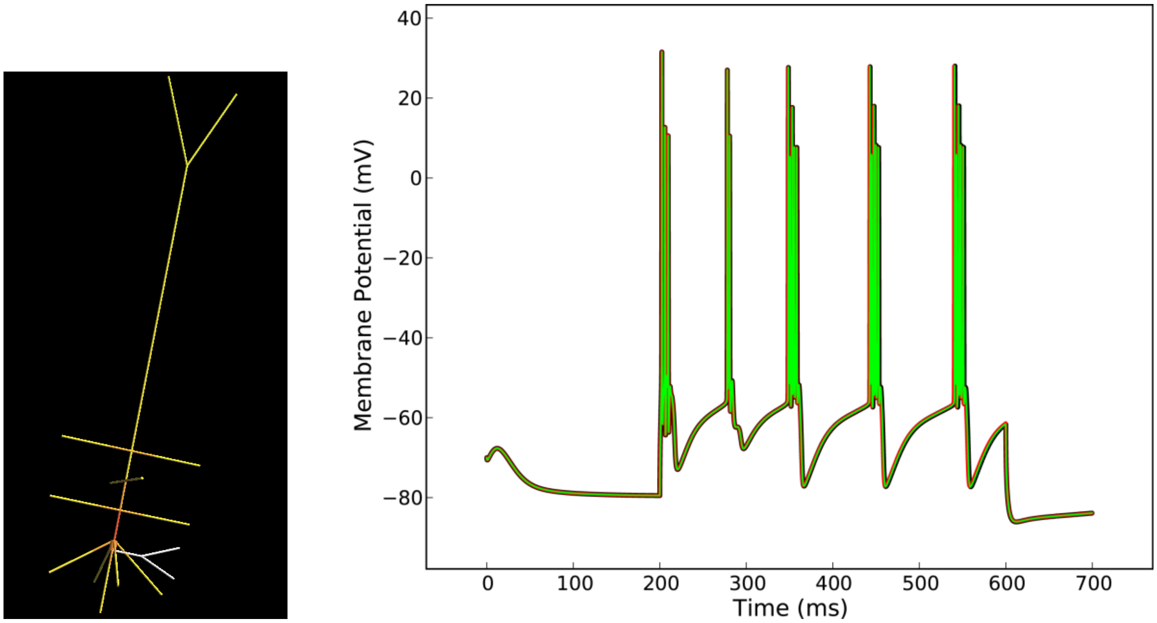

Figure S3vi: Layer 5 Intrinsically Bursting pyramidal cell showing locations of persistent sodium conductance (left). Plot on the right shows simulated behavior on NEURON (black, CVODE variable timestep method) , GENESIS (red, 0.005 ms timestep) and MOOSE (green, 0.005 ms timestep) on application of a hyperpolarizing current (-0.2 nA pre 200ms) followed by a depolarizing current (1.5 nA for 400ms). Time difference between the last spikes is 1.5 ms (0.27 % spike timing shift). There were 7178 numerical integration points in the NEURON simulation (total nseg).

Cell 8: L5TuftedPyrRS

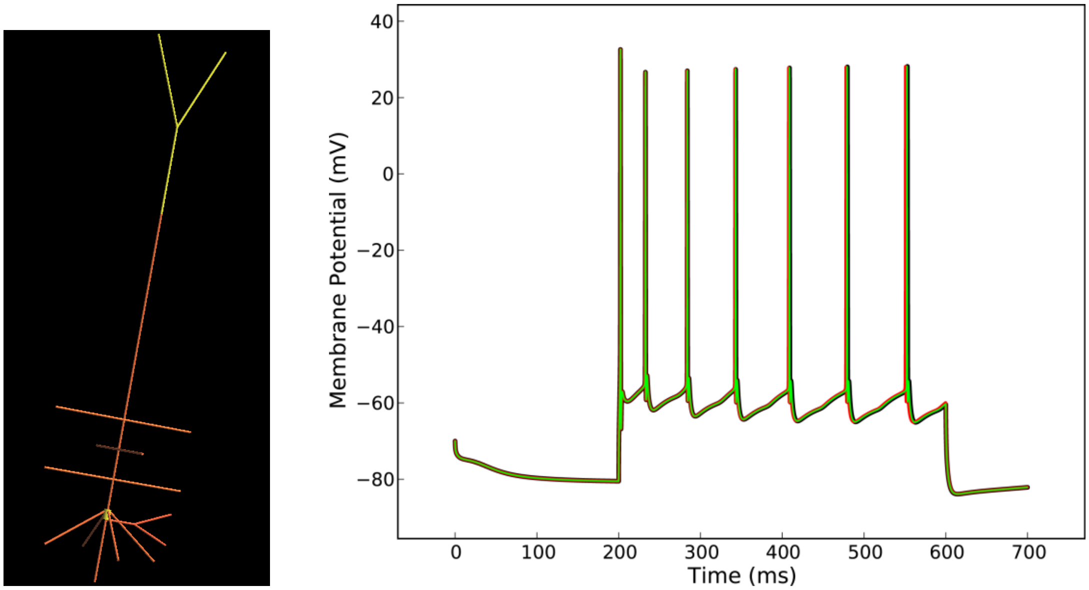

Figure S3vii: Layer 5 Regular Spiking pyramidal cell showing locations of M-type potassium conductance (left). Plot on the right shows simulated behavior on NEURON (black, CVODE variable timestep method) , GENESIS (red, 0.005 ms timestep) and MOOSE (green, 0.005 ms timestep) on application of a hyperpolarizing current (-0.4 nA pre 200ms) followed by a depolarizing current (1.4 nA for 400ms). Time difference between the last spikes is 2.6 ms (0.47 % spike timing shift). There were 7178 numerical integration points in the NEURON simulation (total nseg).

Cell 9: L6NonTuftedPyrRS

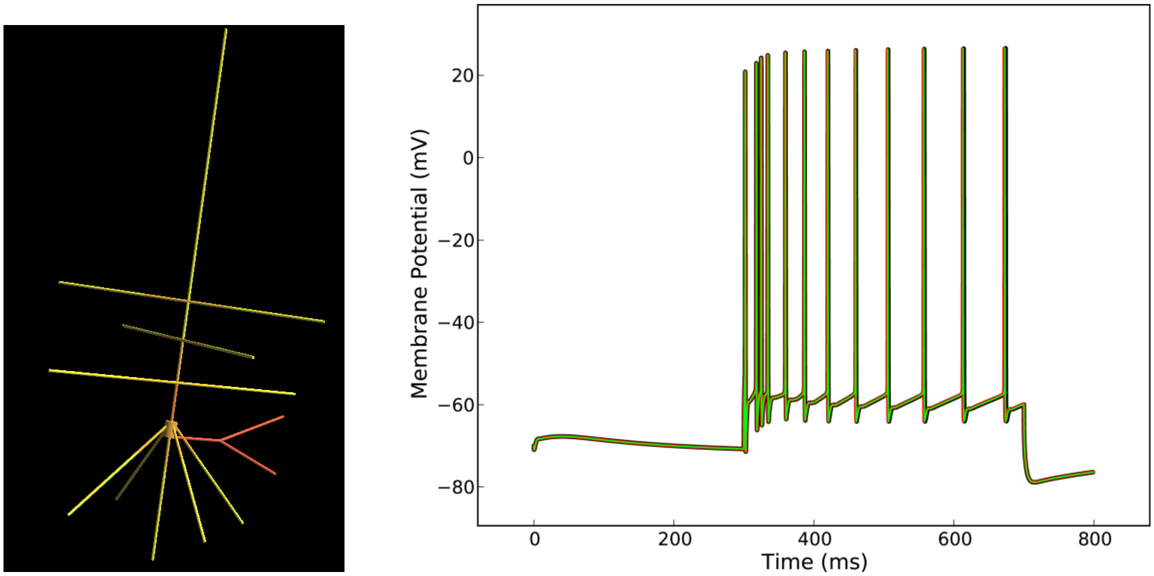

Figure S3viii: Layer 6 Non Tufted Regular Spiking pyramidal cell showing locations of fast Na^+ conductance (left). Plot on the right shows simulated behavior on NEURON (black, CVODE variable timestep method) , GENESIS (red, 0.005 ms timestep) and MOOSE (green, 0.005 ms timestep) on application of a hyperpolarizing current (-0.25 nA pre 300ms) followed by a depolarizing current (1.0 nA for 400ms). The difference in the timing of the last spike is 2.16 ms (0.32% spike timing shift). There were 11423 numerical integration points in the NEURON simulation (total nseg).

Cell 13: TCR

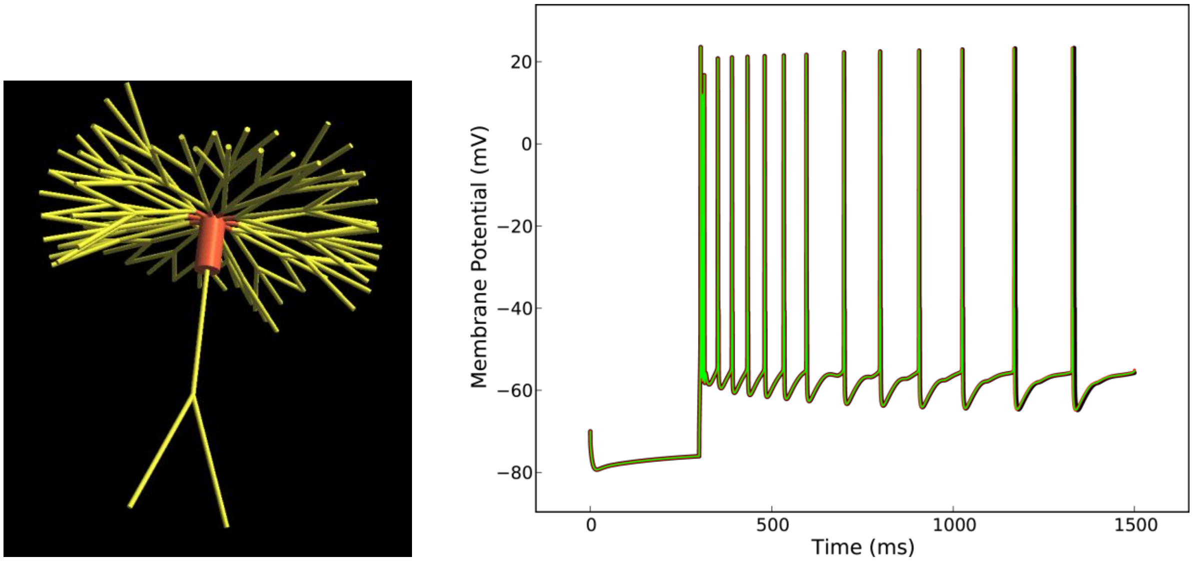

Figure S3ix: Thalamocortical Relay cell showing locations of A-type potassium conductances (left). Plot on the right shows simulated behavior on NEURON (black, CVODE variable timestep method), GENESIS (red, 0.005 ms timestep) and MOOSE (green, 0.005 ms timestep) on application of a hyperpolarizing current (-0.9 nA pre 300ms) followed by a depolarizing current (0.6 nA). Timing difference between last NEURON and GENESIS spike was 5.2 ms (0.39% spike timing shift). There were 27448 numerical integration points in the NEURON simulation (total nseg).

Cell 14: nRT

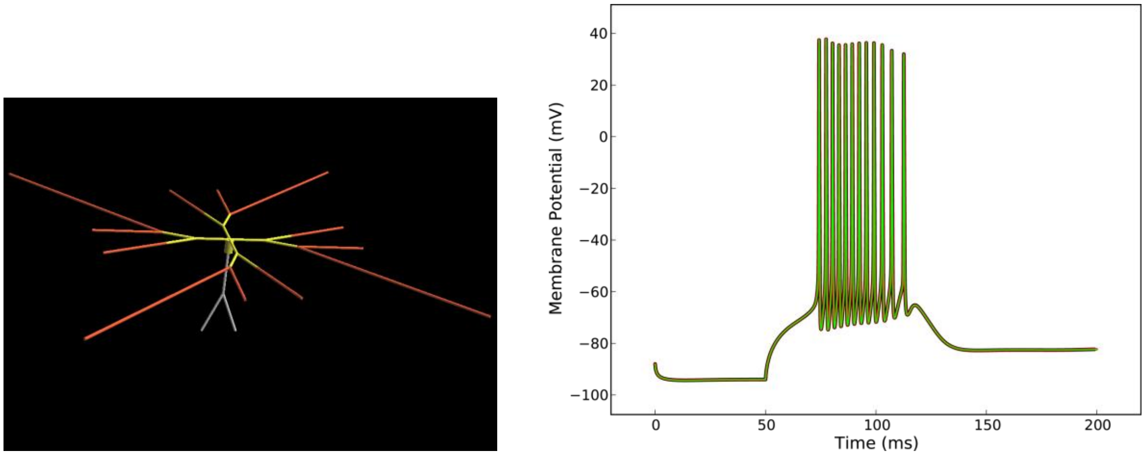

Figure S3x: Nucleus reticularis thalami cell showing locations of the T-type Ca^{2+} conductance (left). Plot on the right below shows simulated behavior on NEURON (black, CVODE variable timestep method) , GENESIS (red, 0.002 ms timestep) and MOOSE (green, 0.002 ms timestep) on application of a hyperpolarizing current (-0.4 nA pre 200ms) followed by a zero input current. Difference in timing of final spike was 0.018 ms (0.02% spike timing shift). There were 2124 numerical integration points in the NEURON simulation (total nseg).
