# Supplementary material for: NeuroML: A Language for Describing Data Driven Models of Neurons and Networks with a High Degree of Biological Detail
Source: PLoS Comput Biol. 2010 Jun 17;6(6):e1000815. doi: 10.1371/journal.pcbi.1000815 (PMC2887454; doi:10.1371/journal.pcbi.1000815)
Supplement: Figure S4 — Convergence of model behavior for fine spatial discretisation (1.56 MB PDF) [file pcbi.1000815.s005.pdf]

Supporting Figure S4: Convergence of model behavior for fine spatial discretisation

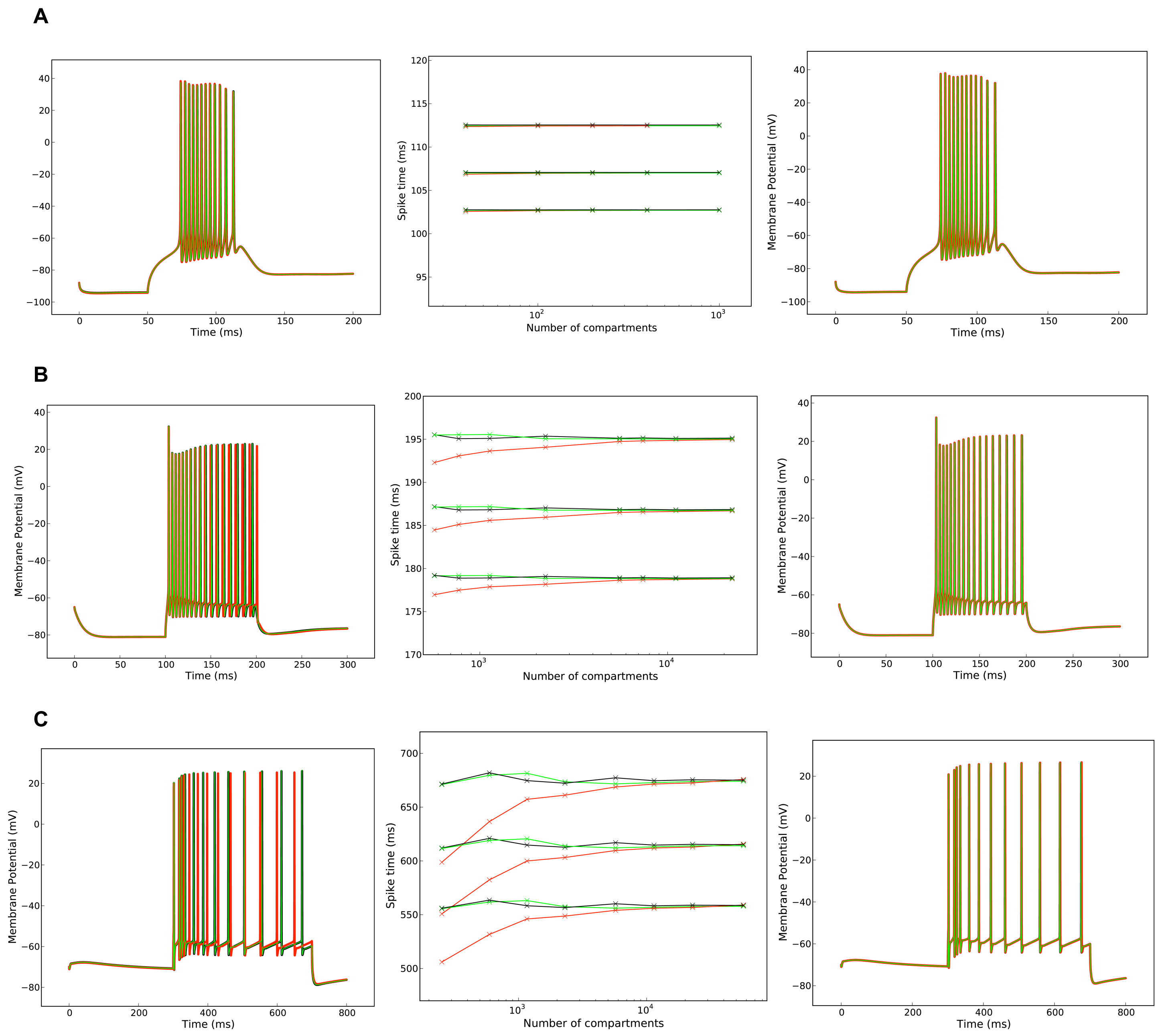

Figure S4: Comparison of cell models under two compartmentalization schemes, as spatial dicretisation is made finer. Results with symmetric compartments used NEURON (black) and MOOSE (green) and those with asymmetric compartments used GENESIS (red). A) Nucleus reticularis thalami (nRT) cell run with total number of numerical simulation points (total nseg) in NEURON between 446 (left) and 4222 (right). Note that the number of compartments in GENESIS/MOOSE corresponds roughly to this figure, see Materials and Methods. Center plot shows the times of the last 3 spikes. B) Superficial Low Threshold spiking (LTS) cell with plots as in A. The left traces are for a cell with 573 numerical simulation points, the right is for 22094. C) Layer 6 Non-tufted Regular Spiking pyramidal cell with plots as in A. The left traces are for a cell with 252 numerical simulation points, the right is for 56976. Note that the increased spatial discretisation is applied uniformly across the cell based on the passive properties. Better algorithms for deciding spatial discretisation on each region of the cell based on local densities of active conductances (possibly customized for each simulator) should lead to convergence of spike traces with smaller number of numerical simulation points/compartments.
