# Supplementary material for: NeuroML: A Language for Describing Data Driven Models of Neurons and Networks with a High Degree of Biological Detail
Source: PLoS Comput Biol. 2010 Jun 17;6(6):e1000815. doi: 10.1371/journal.pcbi.1000815 (PMC2887454; doi:10.1371/journal.pcbi.1000815)
Supplement: Figure S5 — Network model behavior with longer timestep and coarser spatial discretisation (3.51 MB PDF) [file pcbi.1000815.s006.pdf]

Supporting Figure S5: Network model behavior with longer timestep and coarser spatial discretisation

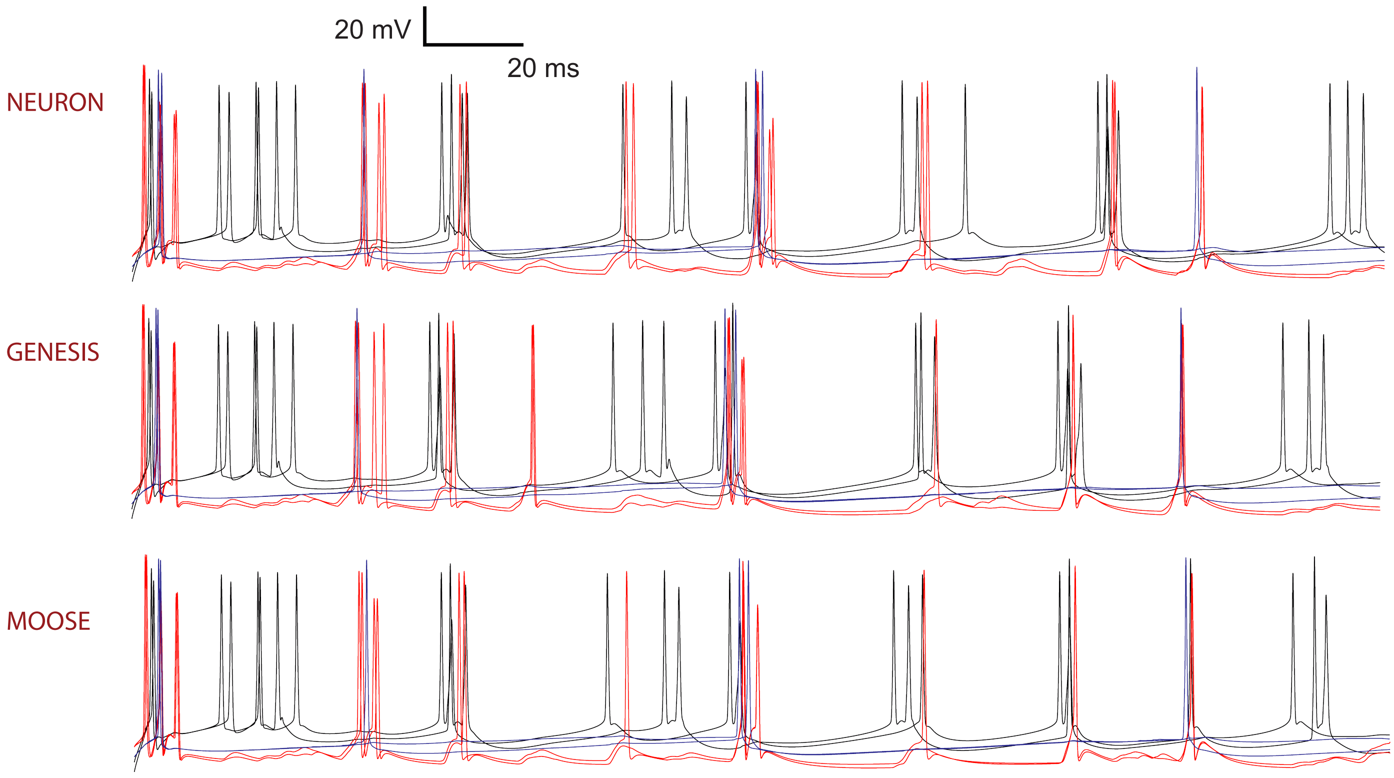

Figure S5: Network simulation as in Figure 10. Simulation time step was 0.01 ms, ten times higher than Fig. 10, but still a commonly used value for simulation timestep. The spatial discretisation was approximately halved: values for numbers of numerical integeration points in NEURON for RS cell, FRB cell, and interneurons respectively in Fig. 10 were 1300, 769 and 1130; the values used here were 758, 383 and 573.
