# Supplementary material for: NeuroML: A Language for Describing Data Driven Models of Neurons and Networks with a High Degree of Biological Detail
Source: PLoS Comput Biol. 2010 Jun 17;6(6):e1000815. doi: 10.1371/journal.pcbi.1000815 (PMC2887454; doi:10.1371/journal.pcbi.1000815)
Supplement: Table S1 — List of thalamocortical cell models from Traub et al., 2005 (0.01 MB PDF) [file pcbi.1000815.s007.pdf]

Supporting Table S1: List of thalamocortical cell models from Traub et al., 2005

	Cell description	Short cell name	
Cell 1	Layer 2/3 Regular Spiking pyramidal cell	L23PyrRS	
Cell 2	Layer 2/3 Fast Rhythmically Bursting pyramidal cell	L23PyrFRB	
Cell 3	Superficial Basket cell	SupBasket	*
Cell 4	Superficial Axo-axonic cell	SupAxAx	*
Cell 5	Superficial Low Threshold spiking cell	SupLTSInter	^
Cell 6	Layer 4 Spiny Stellate cell	L4SpinyStellate	
Cell 7	Layer 5 Tufted Intrinsically Bursting pyramidal cell	L5TuftedPyrIB	
Cell 8	Layer 5 Tufted Regular Spiking pyramidal cell	L5TuftedPyrRS	
Cell 9	Layer 6 Non-tufted Regular Spiking pyramidal cell	L6NonTuftedPyrRS	
Cell 10	Deep Basket cell	DeepBasket	*
Cell 11	Deep Axo-axonic cell	DeepAxAx	*
Cell 12	Deep Low Threshold spiking cell	DeepLTSInter	^
Cell 13	Thalamocortical relay cell	TCR	
Cell 14	Nucleus reticularis thalami cell	nRT	

Cells 3, 4, 10 & 11 (*) all have the same morphologies & channel distributions, as do cells 5 & 12 (^), making 10 cells with individual electrical behavior. These cells are distinguished by their connectivity in the full network model of (Traub et al., 2005).
