# Supplementary material for: NeuroML: A Language for Describing Data Driven Models of Neurons and Networks with a High Degree of Biological Detail
Source: PLoS Comput Biol. 2010 Jun 17;6(6):e1000815. doi: 10.1371/journal.pcbi.1000815 (PMC2887454; doi:10.1371/journal.pcbi.1000815)
Supplement: Table S2 — List of conductances used in thalamocortical cell models from Traub et al., 2005 (0.01 MB PDF) [file pcbi.1000815.s008.pdf]

Supporting Table S2: List of conductances used in thalamocortical cell models from Traub et al., 2005

Short name	Description
**ar**	Anomalous rectifier/H-conductance
**cal**	High threshold, long lasting Calcium L-type conductance
**cat**	Low threshold, inactivating Calcium T-type ("transient") conductance
**cat_a**	Slight modification of above conductance for LTS cells
**k2**	Potassium K2-type conductance (slowly activating and inactivating)
**ka**	Potassium A-type conductance (transient, inactivating)
**ka_ib**	Slight modification of above conductance for IB cells
**kahp**	[Ca^{2+}] dependent K^{+} AHP (afterhyperpolarizing) conductance
**kahp_deeppyr**	Slight modification of above conductance for deep pyramidal cells
**kahp_slower**	Slight modification of kap conductance used in a number of cells
**kc**	Fast voltage and [Ca2+] dependent K^{+} conductance (BK channel)
**kc_fast**	Slight modification of above conductance used in a number of cells
**kdr**	Delayed rectifier potassium conductance
**kdr_fs**	Slight modification of above conductance used in a number of cells
**km**	Potassium M type current (muscarinic receptor-suppressed)
**naf**	Fast sodium transient (inactivating) current
**naf2**	Slight modification of above conductance used in a number of cells
**naf_tcr**	Slight modification of naf conductance used in TCR cells
**nap**	Persistent (non-inactivating) sodium conductance
**napf**	Slight modification of above conductance used in a number of cells
**napf_spinstell**	Slight modification of above conductance used in L4SpinyStellate
**napf_tcr**	Slight modification of above conductance used in TCR
**pas**	Passive leak conductance
**cad**	Exponentially decaying pool of internal calcium
