# Supplementary material for: NeuroML: A Language for Describing Data Driven Models of Neurons and Networks with a High Degree of Biological Detail
Source: PLoS Comput Biol. 2010 Jun 17;6(6):e1000815. doi: 10.1371/journal.pcbi.1000815 (PMC2887454; doi:10.1371/journal.pcbi.1000815)
Supplement: Table S3 — List of cell populations in reduced Layer 2/3 network (0.01 MB PDF) [file pcbi.1000815.s009.pdf]

Table S3: List of cell populations in reduced Layer 2/3 network

Population	Cell type	Number in population
CG_C04_RS_sm	L23PyrRS	20
CG_C04_FRB_sm	L23PyrFRB	6
CG_C04_AxAx_sm	SupAxAx	10
CG_C04_Bask_sm	SupBasket	10
CG_C04_LTS_sm	SupLTSInter	10

Names of the populations/cell groups as used in the neuroConstruct project for the reduced L2/3 network model, the types of cell used in each (see Supporting Table S1) and the number of cells in each population.
