# Supplementary material for: NeuroML: A Language for Describing Data Driven Models of Neurons and Networks with a High Degree of Biological Detail
Source: PLoS Comput Biol. 2010 Jun 17;6(6):e1000815. doi: 10.1371/journal.pcbi.1000815 (PMC2887454; doi:10.1371/journal.pcbi.1000815)
Supplement: Table S4 — List of network connections in reduced Layer 2/3 network (0.01 MB PDF) [file pcbi.1000815.s010.pdf]

Table S4: List of network connections in reduced Layer 2/3 network

Presynaptic →	L23PyrRS	L23PyrFRB	SupAxAx	SupBasket	SupLTSInter
L23PyrRS	**26g, 900e**	**8g, 100e**	**400i**	**400i**	**1200i**
L23PyrFRB	**1g, 270e**	**1g, 30e**	**120i**	**120i**	**360i**
SupAxAx	**900e**	**100e**	**20g**	**200i**	**200i**
SupBasket	**900e**	**100e**		**20g, 200i**	**200i**
SupLTSInter	**900e**	**100e**		**200i**	**20g, 200i**

Total numbers of synaptic connections between pre and post synaptic populations. Excitatory connections are suffixed by **e**, inhibitory connections by **i**, electrical synaptic connections at gap junctions by **g**.
